# Highly Aligned Graphene Aerogels for Multifunctional Composites

**DOI:** 10.1007/s40820-024-01357-w

**Published:** 2024-02-15

**Authors:** Ying Wu, Chao An, Yaru Guo, Yangyang Zong, Naisheng Jiang, Qingbin Zheng, Zhong-Zhen Yu

**Affiliations:** 1 Beijing Advanced Innovation Center for Materials Genome Engineering, School of Materials Science and Engineering, University of Science and Technology Beijing, Beijing, 100083 People’s Republic of China; 2 Institute of Materials Intelligent Technology, Liaoning Academy of Materials, Shenyang, 110004 People’s Republic of China; 3 School of Science and Engineering, The Chinese University of Hong Kong, Shenzhen, Shenzhen, Guangdong 518172 People’s Republic of China; 4 State Key Laboratory of Organic-Inorganic Composites, College of Materials Science and Engineering, Beijing University of Chemical Technology, Beijing, 100029 People’s Republic of China

**Keywords:** Highly aligned graphene aerogels, Quantitative characterization of alignment, Multifunctional composites, Anisotropic properties, Multifunctional applications

## Abstract

Stemming from the unique in-plane honeycomb lattice structure and the *sp*^*2*^ hybridized carbon atoms bonded by exceptionally strong carbon–carbon bonds, graphene exhibits remarkable anisotropic electrical, mechanical, and thermal properties. To maximize the utilization of graphene's in-plane properties, pre-constructed and aligned structures, such as oriented aerogels, films, and fibers, have been designed. The unique combination of aligned structure, high surface area, excellent electrical conductivity, mechanical stability, thermal conductivity, and porous nature of highly aligned graphene aerogels allows for tailored and enhanced performance in specific directions, enabling advancements in diverse fields. This review provides a comprehensive overview of recent advances in highly aligned graphene aerogels and their composites. It highlights the fabrication methods of aligned graphene aerogels and the optimization of alignment which can be estimated both qualitatively and quantitatively. The oriented scaffolds endow graphene aerogels and their composites with anisotropic properties, showing enhanced electrical, mechanical, and thermal properties along the alignment at the sacrifice of the perpendicular direction. This review showcases remarkable properties and applications of aligned graphene aerogels and their composites, such as their suitability for electronics, environmental applications, thermal management, and energy storage. Challenges and potential opportunities are proposed to offer new insights into prospects of this material.

## Highlights

- Aligned graphene building blocks take full advantages of the outstanding properties of graphene.
- Comprehensive review of recent advancements in the utilization of highly aligned graphene aerogels for multifunctional applications.
- By systematically summarizing the controlled assembly, aligned structural attributes, quantitative characterization methods, anisotropic properties, and multifunctional applications of graphene aerogels, this review enhances understanding of the material's potential for diverse applications, offering tailored properties and novel functionalities.

## Graphical abstract

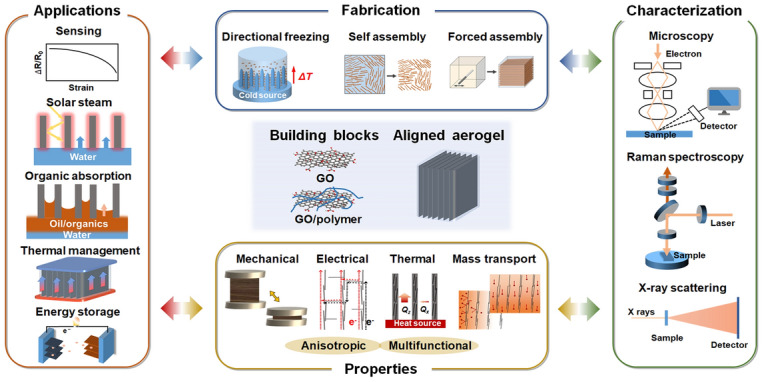

## Introduction

Graphene aerogels have emerged as a highly promising class of materials due to their exceptional properties and diverse applications. Their lightweight and highly porous structures which are composed of interconnected graphene sheets exhibit remarkable mechanical [1], electrical [2], thermal [3], and photo-thermal [4] characteristics. However, the random orientation of graphene sheets in conventional graphene aerogels limits their full potential and hinders the exploitation of their intrinsic properties [5, 6]. In response to this challenge, recent research efforts have focused on developing highly aligned graphene aerogels, where the preferential alignment of graphene sheets within a three-dimensional (3D) network structure offers unique advantages and paves the way for novel functionalities.

Alignment plays a pivotal role in enhancing the properties and performance of graphene aerogels [7–9]. Highly aligned graphene aerogels are characterized by the preferential orientation of individual graphene sheets [10], resulting in a high aspect ratio and a common directionality. This controlled alignment imparts several notable benefits over their randomly oriented counterparts. Firstly, the aligned structure of graphene aerogels significantly enhances their mechanical strength and stiffness, making them capable of withstanding higher loads and exhibiting remarkable structural integrity [11, 12]. This enhanced mechanical robustness is particularly advantageous for applications requiring materials with high strength-to-weight ratios, such as lightweight structural components, flexible electronics, and aerospace materials. Secondly, alignment greatly influences the electrical conductivity of graphene aerogels [13]. The ordered arrangement of graphene sheets allows for efficient charge transport pathways along the aligned direction, resulting in anisotropic electrical conductivity. This can be applied in electronic devices, such as field-effect transistors, where aligned graphene aerogels enable improved charge mobility and device performance. Moreover, the anisotropic conductivity of aligned graphene aerogels facilitates the development of directional electronic devices and sensors [14], opening up new avenues for electronic and sensing applications. Thirdly, alignment plays a vital role in facilitating efficient heat and mass transfer within graphene aerogels [15]. The highly ordered structure enables preferential pathways for thermal conduction, enabling rapid heat dissipation and effective thermal management. Furthermore, the aligned channels within the aerogel structure provide efficient transport pathways for gases and liquids, making aligned graphene aerogels suitable for applications such as thermal steam and organic absorption.

Significant progress has been made in the synthesis and characterization of highly aligned graphene aerogels. Researchers have employed various techniques, including directional freeze casting [16], self-assembly technique [17], and shear-induced alignment to achieve controlled alignment of graphene sheets. These methods allow precise tuning of the alignment degree and directionality, offering the ability to tailor properties of graphene aerogels for specific applications. Given the multitude of advantages offered by highly aligned graphene aerogels, their applications span across diverse fields. The highly aligned graphene aerogels and their composites have been explored as electrodes in energy storage devices, such as supercapacitors and lithium-ion batteries, to enhance their power and energy density. The anisotropic conductivity and efficient transport properties of aligned graphene aerogels have also been leveraged in sensors, oil spill cleanup, and templates for the growth of other nanomaterials.

Though many reviews on graphene aerogels and their composites have been published, the role and contribution of alignment in achieving exceptional mechanical, electrical, thermal, and mass transport properties as well as their multifunctional applications of graphene aerogels has not been comprehensively illustrated. While some reviews touch upon the fabrication and multifunctional applications of graphene aerogels and their polymer composites [18, 19], their focus on alignment in graphene aerogels is cursory. Directional freeze-casted graphene aerogel and their applications in energy storage, energy conversion, and environmental protection have been discussed [20], but a comprehensive review on aligning techniques, morphologies, properties, and multifunctional applications is notably absent. Graphene aerogels for electromagnetic interference (EMI) shielding [21, 22], energy conversion and storage applications [23] have been addressed, but the emphasis on alignment is limited. Reviews on 3D-structured thermally conductive composites [24] and oriented fillers for high thermal conductivity nanocomposites [25] delve into aligned graphene aerogels for thermally conductive applications, yet the contributions of alignment to other multifunctional properties and applications are conspicuously absent. Although there is a review covering comprehensive summary on 2D materials, including graphene, hexagonal boron nitride, transition metal dichalcogenides, and transition metal carbides and nitrides (MXene), and their composites for multifunctional applications [26], readers may struggle to construct a full view of aligned graphene aerogels within the broad 2D-material and diverse structure family. Details and comparisons of different aligning techniques, along with strategies for enhancing alignment, which are instructive to material design and fabrication, are also limited [26] and can be further explored. Furthermore, published reviews have not to summarize quantitative characterization techniques for graphene alignment, a crucial aspect for the precise control and optimization of alignment to take full advantages of graphene.

**Fig. 1:**
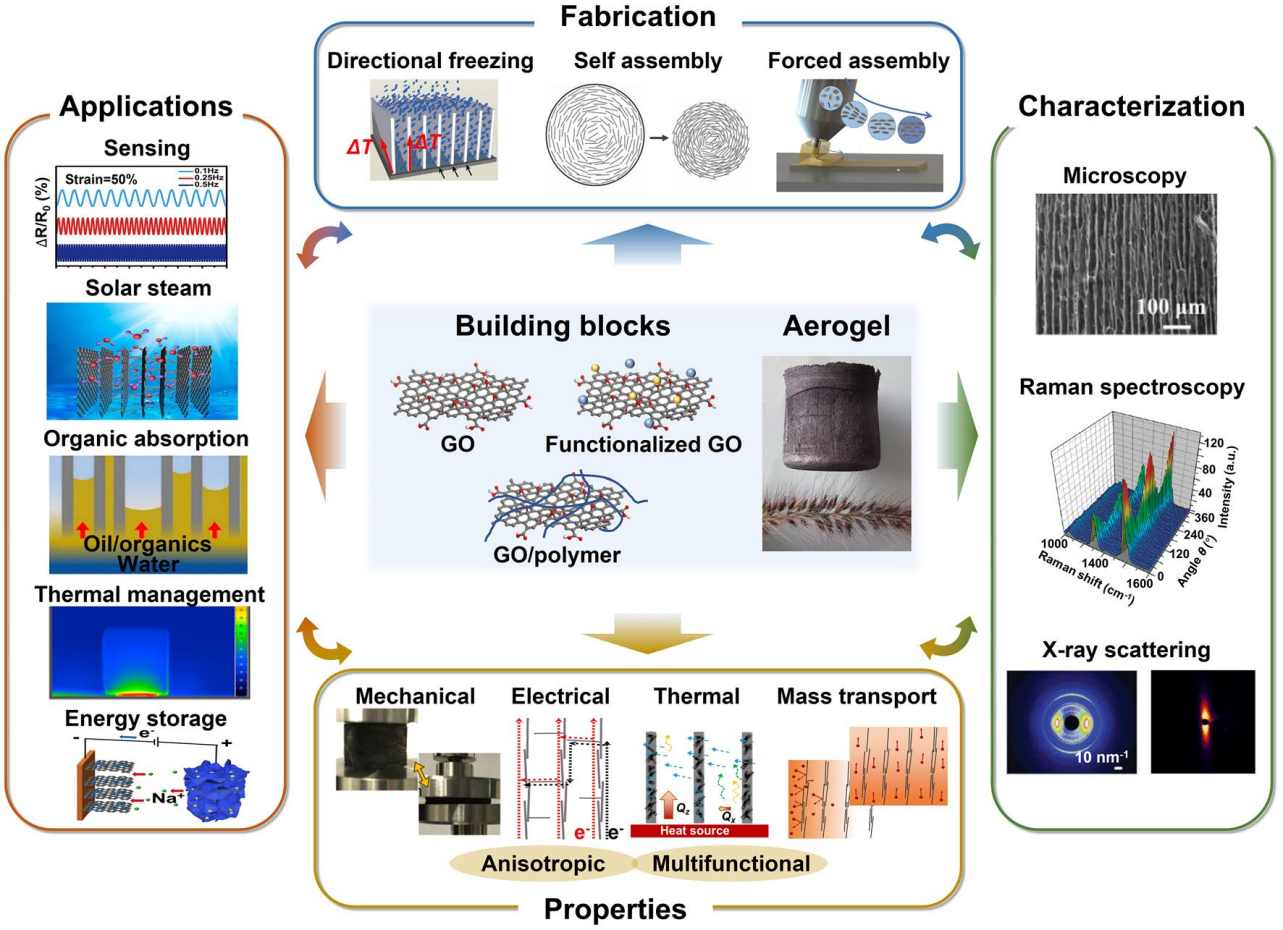
Overview of the highly aligned graphene aerogels for multifunctional composites, from multiscale assembly of aerogel precursors to anisotropic structures, multifunctional properties, and applications. Directional freeze drying. Reproduced under the terms of the Creative Commons CC BY-NC license [27]. Copyright 2018, American Chemical Society. Self-assembly. Reproduced with permission [28]. Copyright 2015, Wiley–VCH. Forced assembly. Reproduced with permission [29]. Copyright 2022, Wiley–VCH. Microscopy, Solar steam. Reproduced with permission [30]. Copyright 2017, American Chemical Society. Raman spectroscopy. Reproduced with permission [31]. Copyright 2011, American Chemical Society. X-ray scattering. Reproduced with permission [32]. Copyright 2020, Wiley–VCH. Mechanical. Reproduced with permission [33]. Copyright 2018, Elsevier. Thermal. Reproduced with permission [34]. Copyright 2022, American Chemical Society. Sensing. Reproduced with permission [35]. Copyright 2021, Elsevier. Organic absorption. Reproduced with permission [36]. Copyright 2021, American Chemical Society. Thermal management. Reproduced with permission [37]. Copyright 2017, American Chemical Society. Energy storage. Reproduced with permission [38]. Copyright 2020, American Chemical Society

This review is dedicated to provide a comprehensive overview of the state-of-art development of highly aligned graphene aerogels for multifunctional composites, with main focuses on controlled assembly, aligned structural characteristics and characterizations, anisotropic properties and multifunctional applications, as shown in Fig. 1. Typical and commonly applied techniques for aligned graphene aerogels are discussed, together with the graphene orientation characteristics of corresponding strategies. Quantitative analysis of the alignment is summarized for more accurate characterizations. Anisotropic multifunctional properties resulted from nano- and microscopically oriented graphene sheets and the contribution of alignment to multifunctional properties are then reviewed. Understanding the significance of alignment in graphene aerogels, researchers have endowed highly aligned graphene aerogel-based composites with multifunctionalities in various fields, such as sensing, thermal management, energy conversion and storage. Through systematic discussions covering aligning techniques, morphologies, quantitative characterizations, properties, and multifunctional applications, this review establishes, for the first time, a comprehensive “processing–structure–property–application” relationship for highly aligned graphene aerogels and composites, drawing a full and insightful picture of this field. By highlighting the recent advancements and discussing the challenges and future directions in this fast-growing research area, this review aims to contribute to broader understanding and utilization of highly aligned graphene aerogels as multifunctional materials with tailored properties and novel functionalities.

## Preparation of Aligned Graphene Aerogels

While pristine graphene possesses outstanding electrical, thermal, and mechanical properties, there are challenges associated with its use in fabricating graphene aerogels. The limited dispersibility of pristine graphene in solvents makes it challenging to achieve uniform gelation and subsequently leads to inhomogeneous aerogel structures [39]. Pristine graphene also lacks surface functional groups, which are crucial for enhancing interfacial interactions and ensuring the compatibility of the graphene sheets during the gelation process. Despite attempts to address these challenges by incorporating surface modifications, surfactants, or polymer binders to prevent the aggregation and to promote the gelation of pristine graphene sheets, aerogels fabricated using pristine graphene building blocks are susceptible to structural shrinkage and inhomogeneity [40–42]. Consequently, the popularity of pristine graphene for aerogel fabrication is diminished.

Alternatively, graphene oxide (GO), a derivative of graphene fabricated via oxidation and exfoliation of natural graphite [43–45], is widely used as a crucial building block in the preparation of graphene aerogels due to its unique properties and solution processability. GO possesses oxygen-containing functional groups, such as hydroxyl, carboxyl and epoxy groups [46, 47], making GO sheets hydrophilic. The hydrophilicity endows GO sheets with an excellent dispersibility in polar solvents and a strong affinity for water molecules, facilitating the formation of stable colloidal suspensions [48]. The presence of oxygen-containing groups disrupts the perfect hexagonal lattice of graphene, leading to the formation of defects and structural disorder [49], which offers increased accessibility for chemical reactions and the absorption of other molecules or nanoparticles. Thanks to the excellent dispersibility, processability, and designability of GO, graphene aerogels fabricated using GO dispersions are remarkably more multifunctional and high-performance than those using graphene building blocks. Therefore, we will focus on recent advances of graphene aerogels prepared using GO dispersions in this review.

**Fig. 2:**
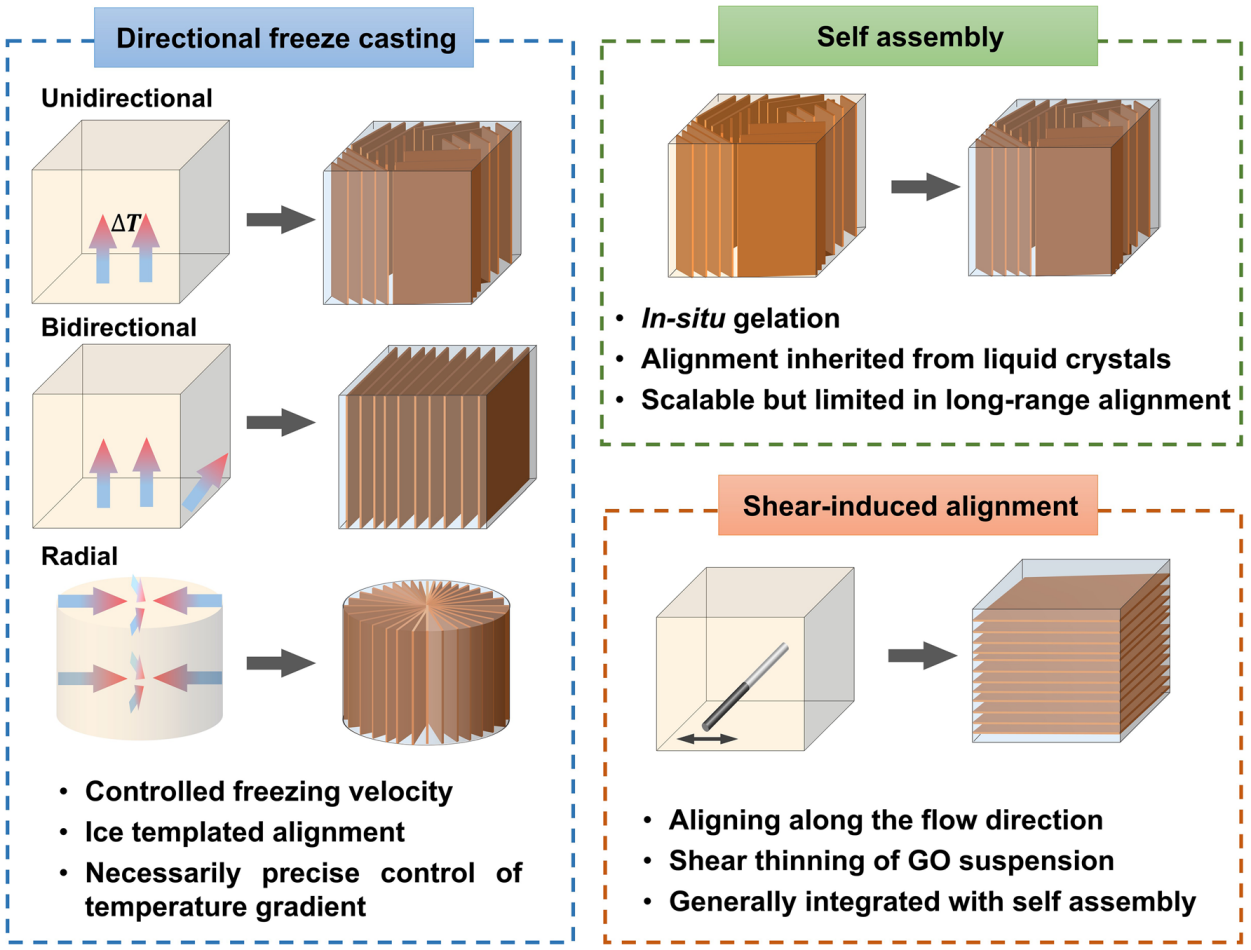
Summary of the most applied techniques for the fabrication of highly aligned graphene aerogels

In order to fabricate highly aligned graphene aerogels, researchers have developed several synthesis methods, as shown in Fig. 2. (i) Directional freeze casting methods involve freezing the graphene dispersion in a controlled manner [50–52]. Ice crystals act as templates, forcing alignment during freeze-casting. Directional freezing enables precise control over alignment direction and complex architectures, while it requires careful control of freezing conditions [53]. Morphologies of graphene aerogels aligned by directional freeze casting are highly dependent on the direction and strength of the temperature gradient. (ii) Self-assembly technique harnesses the inherent oriented characteristics of GO liquid crystals (LCs) which are formed when GO sheets are suspended in a solvent and aligned themselves spontaneously due to their anisotropic properties [54]. This is a facile method for possible large-scale fabrication of graphene aerogels while long-range alignment is difficult. (iii) Shear-induced alignment relies on mechanical forces to align graphene sheets [55]. By subjecting the GO dispersion to shear forces, such as rotational or oscillatory shear, the sheets align along the flow direction. The forced assembly is generally combined with hydrothermal treatment to maintain the alignment. Shear-induced alignment is scalable and straightforward, as it does not require specialized templates, while it is challenging to achieve precise control over alignment degree and direction.

### Directional Freeze Casting Techniques

**Fig. 3:**
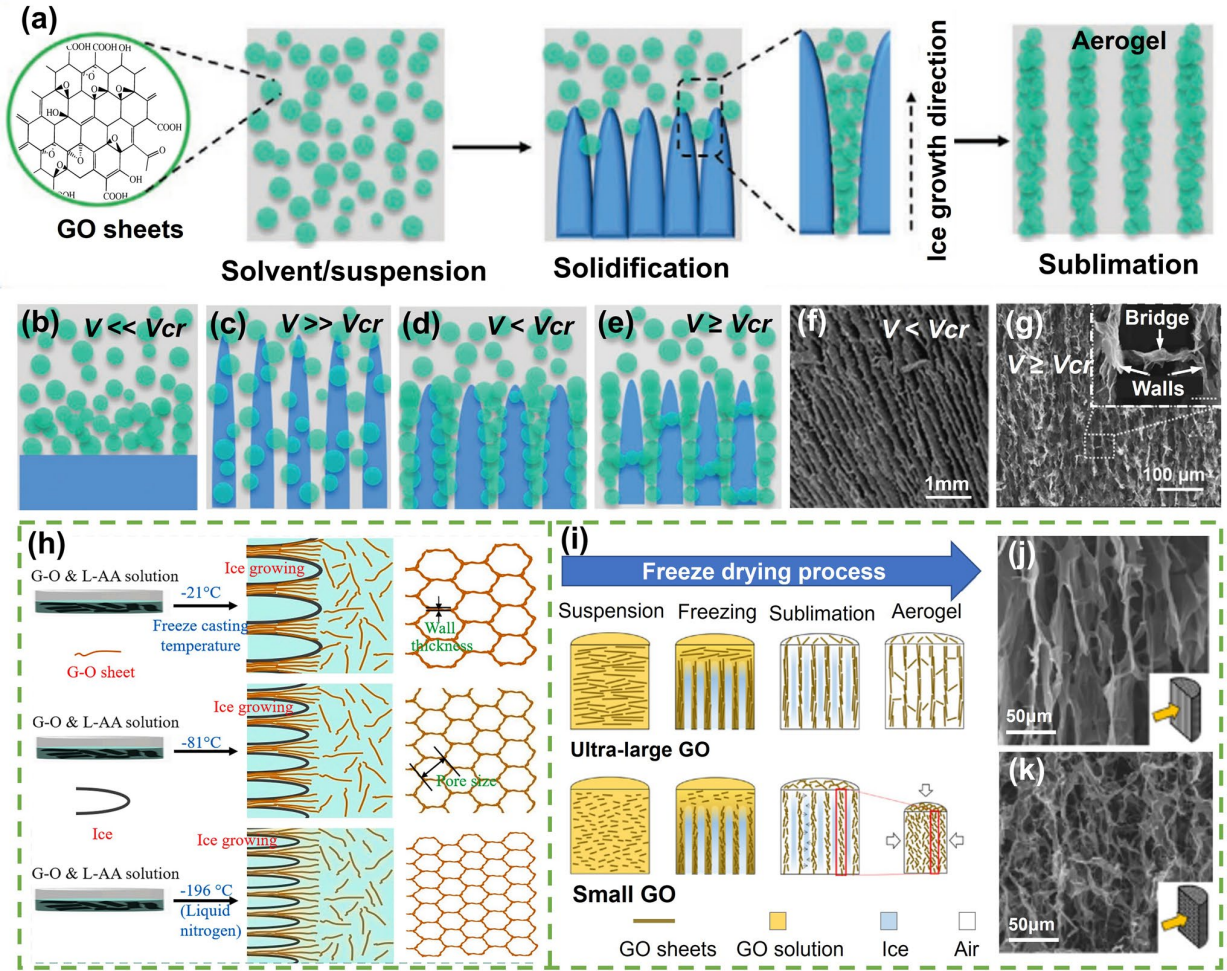
Freeze casting process, mechanism, and morphologies. **a** Schematic process of freeze casting for the fabrication of aligned graphene aerogel; **b–e** The rearrangement of particles in suspensions during the freezing process in different relative values of the freezing velocity (*v*) and the critical freezing front velocity (*v*_*cr*_). Reproduced with permission [57]. Copyright 2020, Wiley–VCH. **f** SEM image of aligned aerogels with lamellar walls at *v* < *v*_*cr*_. Reproduced with permission [61]. Copyright 2021, Elsevier. **g** SEM images of fine-scale interconnected porous alignments at *v* ≥ *v*_*cr*_. Reproduced with permission [42]. Copyright 2018, American Chemical Society. **h** Effects of the freezing temperature (or the freezing speed) on the alignment of graphene aerogels. Reproduced with permission [62]. Copyright 2021, Elsevier. **i–k** Effects of the lateral size of GO building blocks on the alignment of graphene aerogels. Reproduced with permission [65]. Copyright 2018, American Chemical Society

Directional freezing is the most widely employed method for fabricating highly aligned graphene aerogels [56]. This technique involves controlled freezing of a well-dispersed graphene oxide suspension or solution to induce the formation of ice crystals that act as templates for the alignment of graphene sheets (Fig. 3a) [57, 58]. During the directional freezing process, ice crystals start to form and grow along the freezing direction. These ice crystals act as physical templates, imposing directional constraints on the surrounding graphene sheets [59]. The graphene sheets align parallel to the growing ice crystals, following their orientations. This alignment is facilitated by the preferential adsorption of graphene sheets on the ice-water interface during freezing. As the ice crystals gradually increase in size and complexity, they entrap the aligned graphene sheets within their structures. By carefully manipulating the freezing conditions, such as cooling rate, temperature gradient, and freezing direction, the alignment of graphene sheets can be precisely controlled, resulting in highly aligned structures within the final aerogel.

The freeze casting processes rely on the rejection of solid particles by the solidification front to produce aligned or customized porous structures [60]. The morphology of aligned graphene oxide prepared by freeze casting technique is highly related to the freezing front velocity (*v*), as shown in Fig. 3b–e [57]. When the solidification velocity (*v*) is far less than the critical freezing front velocity (*v*_*cr*_), the growth of ice occurs as a planar front, displacing particles and causing an increase in the concentration of solids in the unfrozen region (Fig. 3b). When *v* ≫ *v*_*cr*_, particles are not given sufficient time to segregate from the suspension, resulting in uniformly distributed particles that are embedded throughout the frozen structure (Fig. 3c). Therefore, the freezing front velocity at either *v* ≪ *v*_*cr*_ or *v* ≫ *v*_*cr*_ is not contributive to the construction of highly aligned microscopic structure. When *v* < *v*_*cr*_, particles are generally rejected by the advancing ice front (Fig. 3d) and form lamellar walls within the aerogel, as shown in the scanning electron microscopic (SEM) image in (Fig. 3f) [61]. In the case of *v* ≥ *v*_*cr*_, a certain fraction of particles is typically entrapped by the ice, which leads to the formation of bridges among the lamellar walls (Fig. 3e). A fine-scale porous structure which exhibits interconnected pores due to the presence of particle bridges (Fig. 3g) [42].

Beyond the alignment (in the cases of *v* < *v*_*cr*_ or *v* ≥ *v*_*cr*_), the pore size in graphene aerogels is significantly influenced by the freezing velocity during the fabrication process. Generally, higher freezing velocities result in smaller pore sizes, while lower freezing velocities lead to larger pore sizes (Fig. 3h) [62, 63]. At higher freezing velocities, the freezing process is accelerated, and the liquid precursor or dispersion solidifies rapidly. As a result, smaller ice crystals are formed, which leads to the formation of smaller pores in the graphene aerogel [64]. The rapid freezing does not provide enough time for GO sheets to segregate and move away from the advancing ice front. Consequently, the GO sheets become encapsulated within the ice matrix, resulting in a more uniform distribution of GO and smaller pore sizes in the aerogel structure. Conversely, the lower freezing velocity allows for the growth of larger ice crystals and the more sufficient rearrangement of GO sheets around ice crystals, leading to the formation of larger pores in the graphene aerogel. This is demonstrated by the remarkably larger pore sizes in graphene aerogels frozen at − 21 °C than those at − 196 °C in directions both along and perpendicular to the ice growth direction (Fig. 3h) [63].

Besides the influence of freeze casting parameters on alignment of graphene aerogels, the lateral size of GO precursors is also related to the alignment during the directional freeze casting process [66]. Han et al. fabricated graphene aerogels by directional freeze casting of GO suspensions containing GO sheets with different lateral sizes [65]. Graphene aerogels fabricated by GO dispersions with an average area of 1595.8 μm^2^ show aligned graphene walls (Fig. 3i, j) while those prepared using GO sheets with an average area of 1.1 μm^2^ exhibit random scaffolds (Fig. 3i, k) [65]. The increased inter-sheet spacing and higher aspect ratios of larger GO sheets facilitate a more ordered alignment during the assembly process.

#### Unidirectional Freeze Casting

**Fig. 4:**
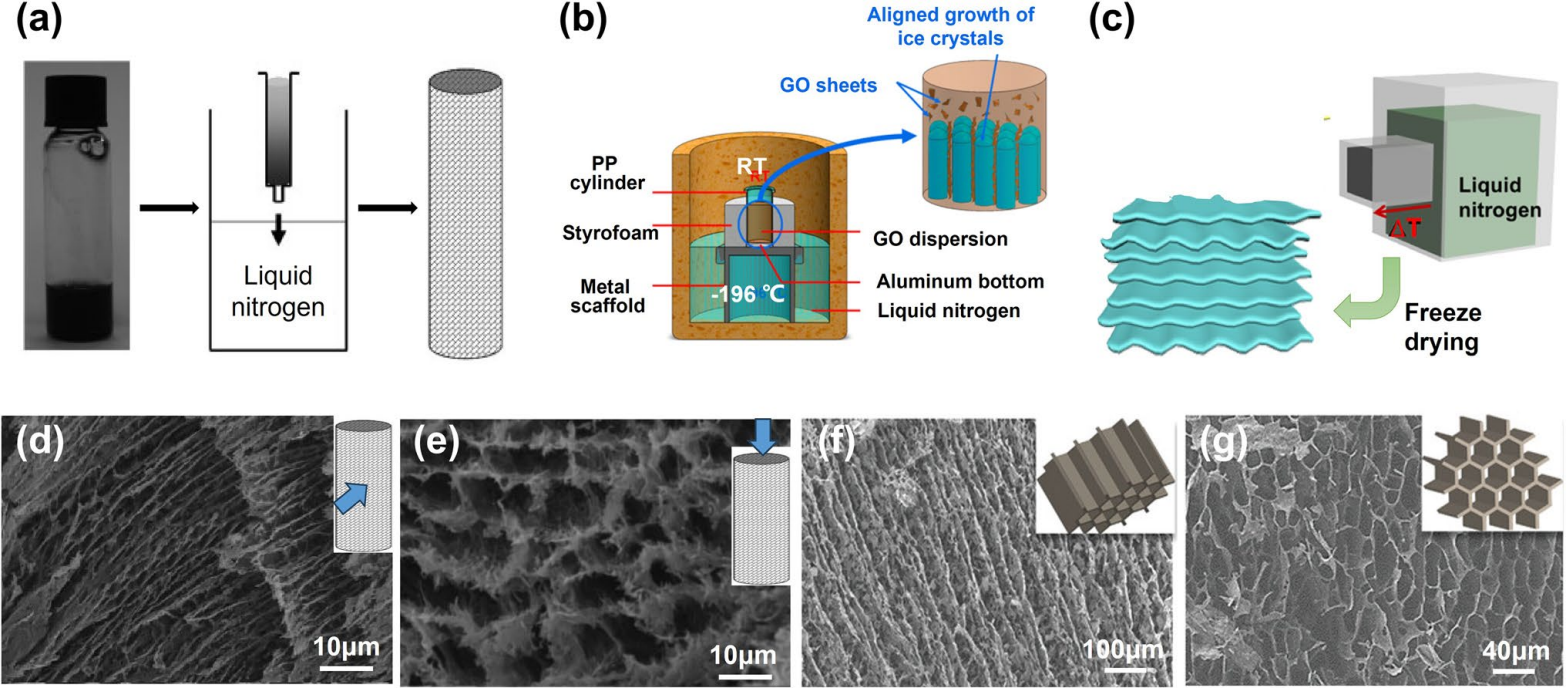
Unidirectional freeze casting techniques and corresponding morphologies. **a** Gradual immersion of GO suspensions in cooling bath. Reproduced with permission [70]. Copyright 2009, Wiley–VCH. **b** A schematic apparatus for unidirectional freeze casting of GO suspensions and the formation mechanism of the aligned GO walls in aerogel. Reproduced with permission [5]. Copyright 2016, American Chemical Society. **c** Schematics of lateral unidirectional freeze casting. Reproduced with permission [85]. Copyright 2022, The Authors, published by Springer Nature. **d–g** Typical SEM images of unidirectionally freeze-cast graphene aerogels observed from different directions. The left two reproduced with permission [70]. Copyright 2009, Wiley–VCH. The right two reproduced with permission [83]. Copyright 2022, Wiley–VCH

Unidirectional freeze casting involves the controlled freezing of a liquid precursor or suspension in a single direction, resulting in the formation of a solid with aligned structures [67, 68]. It can be achieved by immersing GO suspensions into a cold bath in a controlled velocity (Fig. 4a) [69, 70], putting suspensions on a cold platform (such as dry ice or metals that are partially immersed in liquid N_2_) (Fig. 4b) [68, 71] or tying dispersions to the side of a lidless metallic box filled with liquid nitrogen (Fig. 4c) [72]. As the temperature decreases, ice crystals start to form and propagate, growing in the direction of the temperature gradient [62, 73]. During the freezing process, the solidification front advances from one end of the mold to the other due to the unidirectional temperature gradient, causing the aligned structures to form parallel to the freezing direction [74, 75]. In the subsequent freeze drying process, ice crystals are sublimated under vacuum at low temperatures and the aligned pores are left in the aerogel [76].

Morphologies of graphene aerogels fabricated via unidirectional freeze casting method are highly anisotropic due to the intrinsic temperature gradient, exhibiting a distinct tubular structure along the freezing direction [77, 78]. Ice crystals nucleate at the interfaces of GO dispersions and containers and grow in a particular direction along the unidirectional temperature gradient. GO sheets are expelled by the in-situ grown ice crystals, resulting in aligned GO sheets at interfaces among ice crystals [79–81]. Consequently, stacked layers or lamellae are formed along the freezing direction. In the plane perpendicular to the freezing direction, the aerogels display a porous structure with interconnected pores [82].

Specifically, in the immersing process, the liquid suspension is typically poured into a mold or container, followed by a controlled cooling procedure that involves a carefully orchestrated immersion into a cryogenic bath at a predetermined velocity. Jamma and colleagues engineered an aligned aerogel composite by immersing a syringe loaded with aqueous GO/polymer suspensions into liquid nitrogen at dipping rates of 2–5.9 mm min^−1^ before the freeze drying and the removal of syringe (Fig. 4a) [70]. The resultant freeze-dried monolith exhibited well-aligned frameworks along the immersing direction (Fig. 4d) and cellular channel-like architectures in the cross-section view (Fig. 4e). The aligned graphene aerogels was achieved by immersing GO suspensions through a continuous injection into liquid nitrogen [55]. This inventive technique combined spinning technology with ice-templating alignment, facilitating the generation of aligned pore structures. By situating a container filled with a GO suspension atop a thermally conductive base and encompassing it with thermally insulating surroundings on a chilled platform, a unidirectional temperature gradient is generated, emanating from the bottom towards the top (as depicted in Fig. 4b). This thermal configuration prompts the vertical growth of ice crystals. Consequently, the resultant graphene aerogel exhibits vertically aligned graphene walls (Fig. 4f), alongside tubular structures displaying comparatively less alignment within the horizontal plane (Fig. 4g) [74, 83]. Affixing the mold to the side of a metallic box filled with liquid nitrogen generates a horizontal temperature gradient (Fig. 4c), fostering the lateral growth of ice crystals and corresponding alignment [84].

#### Bidirectional Freeze Casting

**Fig. 5:**
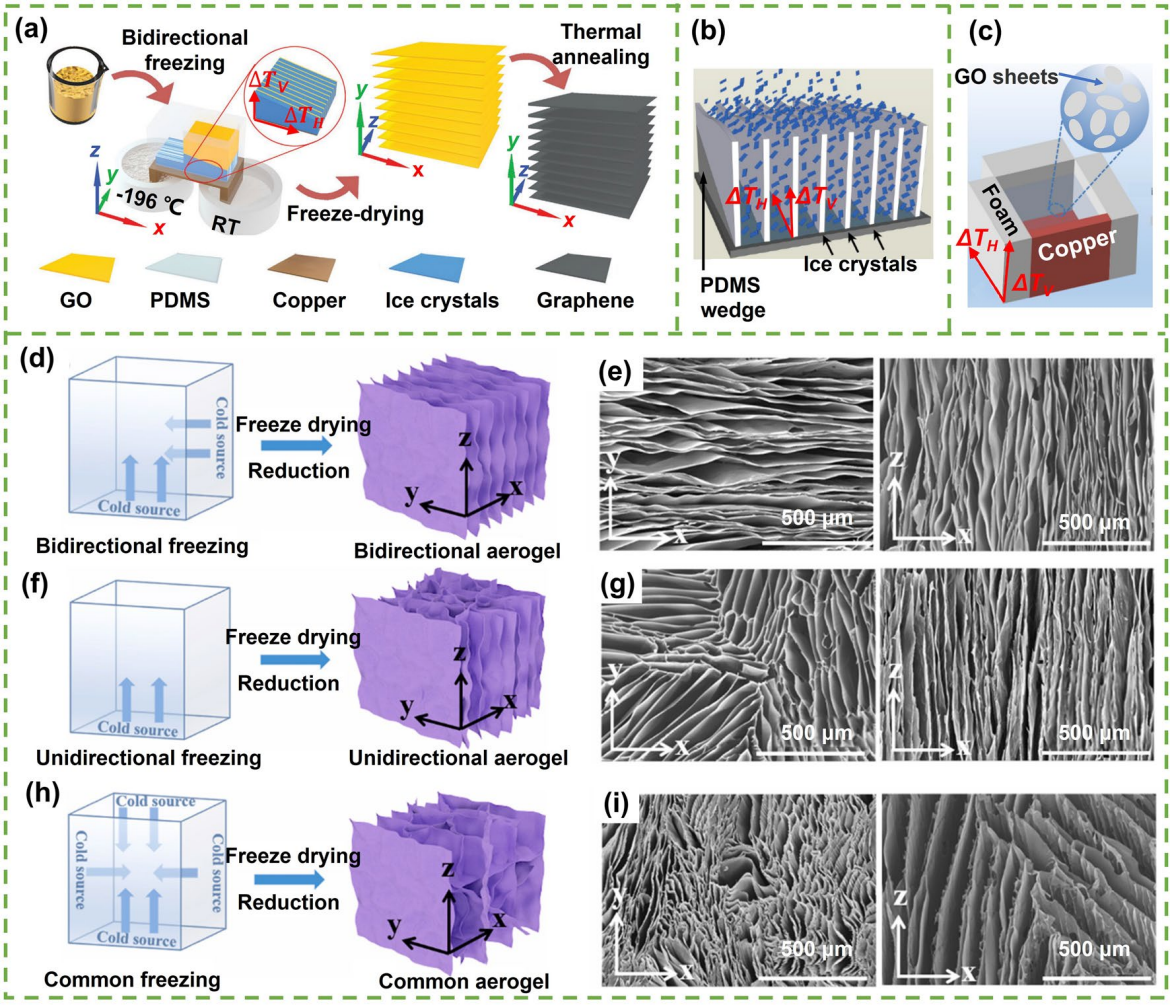
**a–c** Schematic illustrations of three typical bidirectional freeze casting apparatus and processes for the fabrication of highly aligned graphene aerogels. Reproduced with permission [27, 87, 88]. Copyright 2018, American Chemical Society. Copyright 2021, Wiley–VCH. Copyright 2020, Elsevier. Schematic and morphology comparisons of aerogels prepared using **d, e** bidirectional freezing, **f, g** unidirectional freezing and **h, i** common freezing. Reproduced with permission [88]. Copyright 2020, Elsevier

Bidirectional freeze casting is a versatile and promising fabrication technique employed for the production of materials with aligned and ordered microstructures in two perpendicular directions, possible in constructing centimeter-scale long-range ordered 3D structures [27, 86]. Different from conventional or unidirectional freeze casting, bidirectional freeze casting involves temperature gradients simultaneously along horizontal (*ΔT*_*H*_) and vertical (*ΔT*_*V*_) directions (Fig. 5a–c) [27, 87, 88]. During the freezing process, ice crystals gradually grow in both horizontal and vertical orientations, resulting in the formation of microstructures exhibiting aligned features in the two orthogonal directions [27, 89, 90].

An approach involves the use of a thermally conductive copper bridge, partially submerged in liquid nitrogen at one end and exposed to a higher-temperature environment at the other, effectively generating a dual-temperature bridge. Placing GO suspensions onto this bridge results in the creation of a horizontal temperature gradient extending from the liquid-nitrogen side to the other, along with a vertical gradient from the copper bridge to the upper suspension, as shown in Fig. 5a [87]. This thermal configuration leads to the initiation of ice crystal nucleation at the edge adjacent to the liquid nitrogen, followed by growth in both horizontal and vertical orientations. The low-temperature end of the metal plate can be effectively cooled by utilizing substances like liquid nitrogen, dry ice, or chilled ethanol, whereas the high-temperature end can be exposed to air or water for efficient temperature control [59, 89, 91]. The temperature difference between the high- and low-temperature ends, as well as the placement of the mold, determine the temperature gradients in both horizontal and vertical orientations, influencing the inter-lamella spacing within aerogels. After freeze drying and reduction, graphene aerogels with aligned lamella are resulted.

The incorporation of a polymer wedge at the bottom of the mold containing GO suspensions has been proven to be an effective method to precisely adjust the temperature gradient [92, 93]. This bottom-wedged container is situated typically on a metallic platform that is partially immersed in liquid nitrogen or other cold media to attain the necessary freezing temperatures, as illustrated in Fig. 5b [27, 94]. Ice crystals initiate at the lowest edge of the wedge, growing along both the wedge and the vertical direction. The rate of cooling and the slope angle of the wedge collaboratively determine the alignment observed in the final structures. For instance, Bai and colleagues achieved a monodomain structure—a consistent single orientation across the entire sample—with a wedge slope angle of 20° and a cooling rate of 5 or 10 °C min^−1^ [95]. At cooling rates of 5 and 10 °C min^−1^, the alignment was enhanced with increasing wedge angle up to 20°. However, when the cooling rate was low (1 °C min^−1^), no long-range alignment was obtained, regardless of the wedge angle. An open-top cubic mold, comprising a thermally conductive copper bottom, a copper side, and three thermally insulating polymeric foam sides, can also be employed to create a dual-temperature gradient for bidirectional freeze casting, as shown in Fig. 5c [88]. When the mold is in contact with cold sources, the copper bottom and copper side experience notably rapid temperature reduction compared to the three polymeric foam sides, consequently establishing dual temperature gradients extending from the bottom to the top and from the copper to the polymeric foam side.

The comparison of schematic diagrams and morphologies of aerogels fabricated using bidirectional, unidirectional, and conventional freeze casting methods is illustrated in Fig. 5d–i [88]. Among these approaches, the bidirectional freeze casting method featuring dual temperature gradients (Fig. 5d) stands out for its capability to produce a highly organized large-size single-domain lamellar architecture. This architecture displays straight or undulating microstructures that are parallel to the plane of ice crystal growth (the *yz* plane) [96], as evidenced by the alignments observed in the cross section (the *xy* plane) and the longitudinal section (the *xz* plane) (Fig. 5e). Differently, unidirectional freeze casting yields organized microstructures solely along the direction of ice growth (the *xz* plane), while the plane perpendicular to the ice growth (the *xy* plane) exhibits multidomain alignments or honeycomb patterns, as shown in Fig. 5f, g. By comparison, the common freeze casting method (Fig. 5h) results in a less ordered microstructure in both the *xy* and *xz* planes (Fig. 5i). These distinct microstructural variations across the aerogels carry significant implications for their mechanical flexibility and multifunctional characteristics. The highly aligned graphene aerogel fabricated by bidirectional freeze casting holds the potential to possess outstanding properties, attributing to its precisely defined and well-ordered microstructure.

#### Radial Freeze Casting

Radial freeze casting involves a freezing process characterized by a radial temperature gradient typically extending from the outer-container to the center, which prompts ice crystals to grow radially inward [97]. This technique allows researchers to manipulate the freezing dynamics, leading to the creation of porous scaffolds with a distinct radial microstructure reminiscent of the natural cellular tracheid patterns. Drawing inspiration from the remarkably efficient capillary transport of water observed in trees, the concept of radial freeze casting has been harnessed to fabricate porous biomimetic structures that replicate the intricate cellular tracheid arrangements found in conifer trees [98]. These biomimetic structures remarkably exhibit efficient capillary transport properties, similar to the remarkable water-conducting abilities witnessed in actual trees [99].

**Fig. 6:**
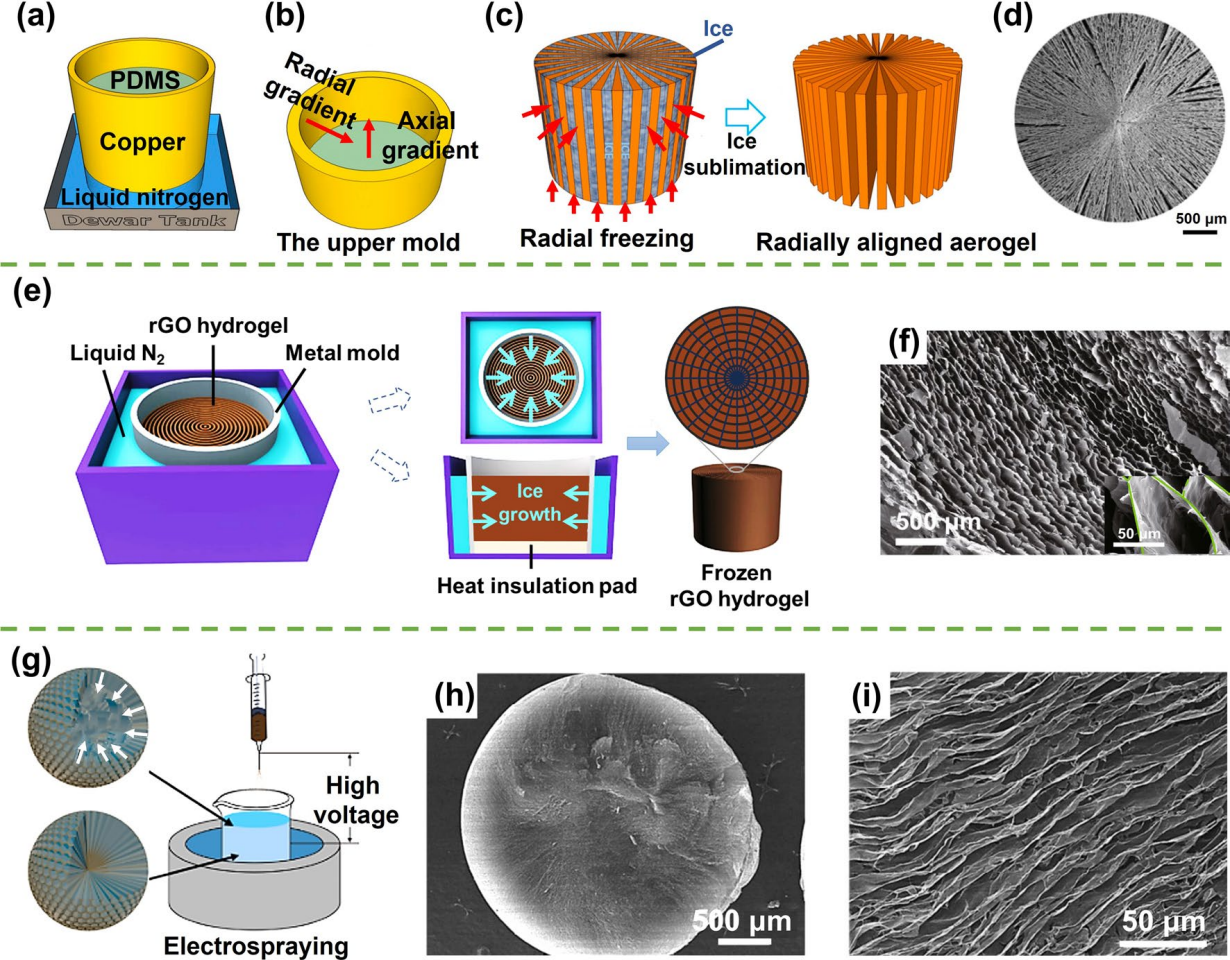
Illustration of **a** freezing setup for fabricating graphene aerogels with radial orientation, **b** the temperature gradient, **c** the freezing-drying process, and **d** SEM morphologies of a radiating graphene aerogel. Reproduced with permission [99]. Copyright 2018, American Chemical Society. **e** Schematic diagram of radial freeze casting of rGO hydrogel and **f** microstructure of corresponding aerogels. Reproduced under the terms of the Creative Commons CC BY license [102]. Copyright 2021, The Authors, published by Springer Nature. **g** Fabrication of radiating graphene aerogel spheres by immersing GO droplets into liquid nitrogen. Reproduced with permission [103]. Copyright 2017, American Chemical Society; and **h, i** their cross-sectional microstructure. Reproduced with permission [104]. Copyright 2016, American Chemical Society

The radial freeze casting technique has been explored to fabricate radially aligned graphene aerogels. This method involves a carefully orchestrated interplay between a thermally conductive, lidless metal mold with a polymer bottom and an immersive frigid environment (such as liquid nitrogen), setting the stage for the generation of a precise radial temperature gradient [100, 101]. The strategic positioning of liquid nitrogen enveloping the lower and surroundings of the thermally conductive metal mold (Fig. 6a) creates a dynamic thermal landscape characterized by dual temperature gradients along both the vertical and the radial directions (Fig. 6b). With a significantly lower thermal conductivity of the polymer bottom than the surrounding metallic container, the ice grows predominantly in the radial direction, while both longitudinal and radial growth of ice crystals happen (Fig. 6c) [99]. After ice sublimation, highly aligned graphene aerogels with radially aligned cross sections and vertically aligned walls are obtained (Fig. 6d).

The radial freezing can also be employed for the rearrangement of building blocks in hydrogels. Lin et al. applied a radial freeze casting approach to convert reduced graphene oxide (rGO) hydrogels characterized by concentric rings into a captivating 3D interconnected network, resembling a spider-web structure [102]. This transformative process unfolded within a specialized metal mold, featuring a thermally insulated polymeric base immersed in liquid nitrogen, as shown in Fig. 6e. During freeze casting, the outer circumference of the mold makes direct contact with the frigid liquid nitrogen, creating a radial temperature gradient that triggers the growth of ice crystals from the mold's periphery towards its center. This promotes the reconfiguration of the previously separate rGO concentric-ring walls, originally formed through hydrothermal self-assembly, into a complex spider-web-like network after freeze-drying, as shown in Fig. 6f.

As elucidated in the “Unidirectional freeze casting” section, the gradual immersion of the GO suspension in liquid nitrogen results in a unidirectional ice growth. However, a captivating shift occurs when plunging the suspension in a cylindrical mold into liquid nitrogen. This swift immersion engenders an instantaneous and uniform exposure of the entire suspension to the frigid milieu, instigating the inception of temperature gradients rippling from the peripheral edges to the core of the cylinder [69]. Consequently, ice crystals grow radially, resulting in radial alignment of graphene sheets in the resultant aerogels.

Similarly, the fabrication of freestanding porous graphene aerogel beads, characterized by radially oriented channels extending from the sphere's surface to its center, was developed through the high-voltage-assisted fast casting of spherical GO suspensions into liquid nitrogen, as shown in Fig. 6g [103–105]. As these GO dispersion droplets were immersed in the frigid liquid nitrogen environment, an immediate and substantial temperature gradient ensued, triggering the rapid nucleation of ice crystals at the sphere's surface. The ice crystals then grew progressively from the surface towards the center of the sphere, as shown in Fig. 6g. A cross-sectional analysis unveils the presence of these radial channels, characterized by meticulously aligned graphene walls that span from the sphere's center to its surface, as showcased in Fig. 6h, i [104]. This distinct microscopic architecture imparts remarkable supercapacitive properties and exceptional water contaminant absorption capabilities to these graphene aerogel spheres, signifying the far-reaching potential of this technique in tailoring advanced material functionalities [103, 104].

### Self-Assembly Induced Alignment

**Fig. 7:**
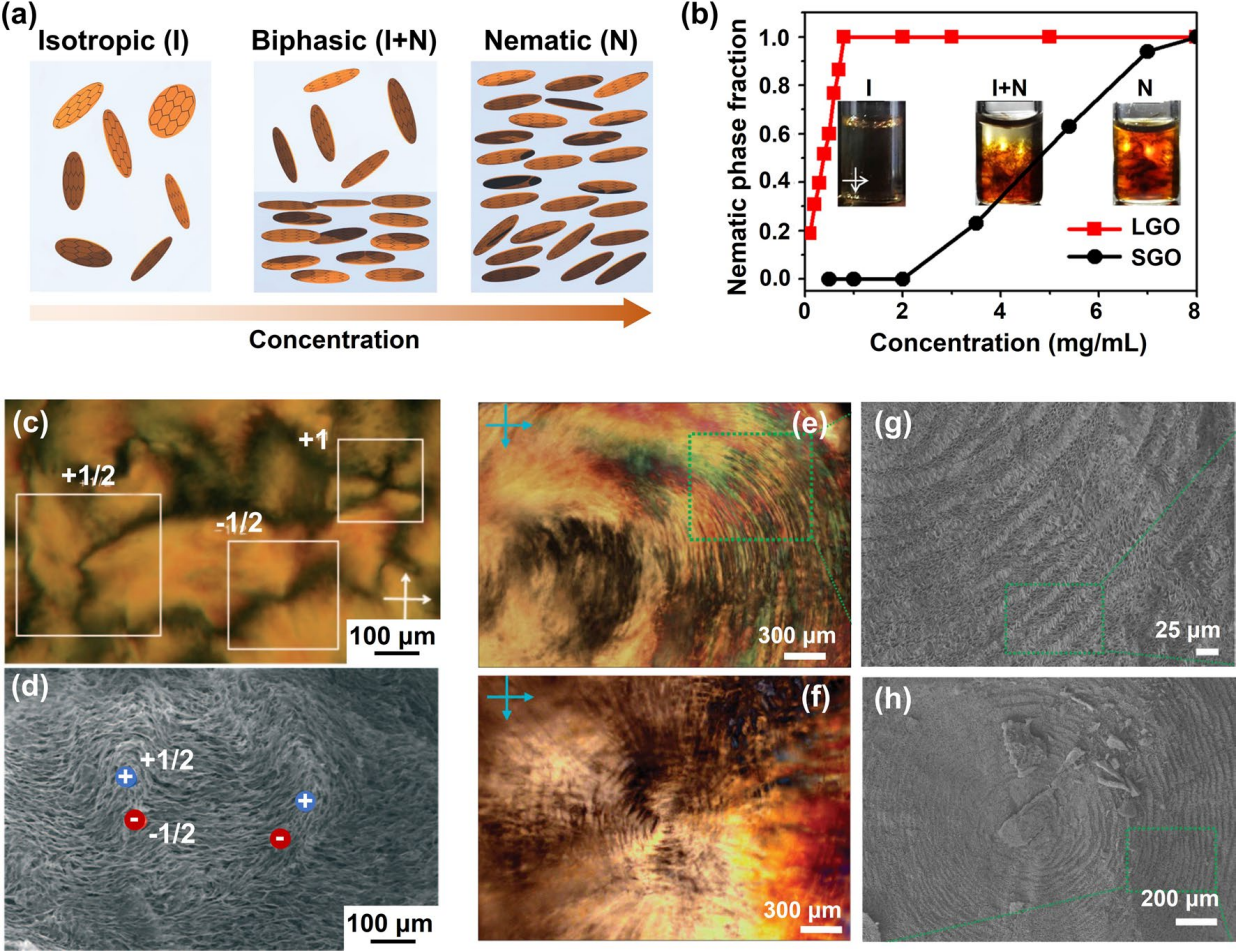
GO LC which is the prerequisite of self-assembly induced graphene alignment. **a** The transition of GO suspensions from isotropic to nematic LCs with increasing concentration. Reproduced with permission [107]. Copyright 2016, Wiley–VCH.** b** Nematic phase fraction in GO suspensions against the concentration. Reproduced with permission [109]. Copyright 2014, American Chemical Society. **c, d** Typical nematic texture and freeze-dried morphology of GO LCs. Reproduced with permission [110]. Copyright 2011, Wiley–VCH. Polarized optical microscopy and Cryo-SEM images of GO LCs in the **e, f** lateral and **g, h** center regions, showing helical structures. Reproduced with permission [113]. Copyright 2011, The Authors, published by Springer Nature

Self-assembly induced alignment in graphene aerogels refers to the process where GO sheets align themselves during the formation of aerogel structure, attributing to unique properties and the inherent ordering tendencies of GO LCs. At lower concentrations, colloids composed of anisotropic particles (here the GO sheets) exhibit an isotropic phase, yet they undergo a transition into a biphasic combination, characterized by the coexistence of both isotropic and nematic phases, as the particle concentration increases [106]. When the concentration of GO sheets reaches the critical transition concentration, a fully formed and distinct nematic phase becomes established, as shown in Fig. 7a [107]. The empirical critical LC concentration, *C*_*LC*_, which is deduced from the well-known Onsager's theory, is determined by [107]:

1 $${C}_{LC}\approx \frac{4T}{W}$$

where *T* and *W* are the thickness and width of GO sheets, respectively. According to the empirical equation, we can conclude that the critical transition concentration of the GO suspensions is highly related to the aspect ratio of GO sheets.

The influence of aspect ratio on the critical concentration for the transition to the nematic phase has been experimentally confirmed through an investigation of the nematic phase fraction in GO suspensions with different sizes of GO sheets [108–110]. At a sufficiently low concentration of 0.1 mg mL^−1^, the large GO (LGO) dispersion with lateral sizes ranging from 0–20 μm remained isotropic, while higher concentrations led to the coexistence of isotropic and nematic phases, resulting in observable macroscopic phase separation after standing for two weeks of self-size separation, as shown in Fig. 7b [109]. The critical concentration for LC transition was determined to be 1.0 mg mL^−1^ for LGO dispersions. In contrast, the nematic phase in small GO (SGO with lateral sizes ranging from 0–5 μm) suspensions emerged at 2.0 mg mL^−1^, with the complete transition to an all-liquid–crystal phase occurring at 8 mg mL^−1^, notably higher concentrations compared to those observed for LGO suspensions.

These distinct textures observed within GO LCs serve as the essential foundation for the fabrication of highly aligned graphene aerogels through self-assembly techniques. By varying concentrations above the *C*_*LC*_ and employing different aspect ratios of GO sheets, a diverse array of textures can be achieved within the LC, directly influencing the eventual microscopic orientation of the self-assembled building blocks in the resultant aerogel [111]. Notably, GO LCs with a mean diameter of 1.65 μm and concentrations ranging from 0.3 to 0.5 wt% exhibited Schlieren textures characterized by disclinations of varying signs and strengths (Fig. 7c), effectively reflecting the local orientation of the GO sheets [112]. Correspondingly, SEM images of freeze-dried textured GO LCs revealed a consistent alignment of graphene around a few ± 1/2 disclinations (Fig. 7d), which is consistent with those in LCs. An intriguing long-range helical structure was also observed by Xu et al. within GO LCs [113]. Specifically, at a GO concentration of 0.98 vol%, surpassing the *C*_*LC*_ of 0.23 vol%, the GO LC displayed a distinct fingerprint texture, as shown in Fig. 7e, f. This unique texture was further corroborated by freeze-fracture cross-sectional SEM morphologies, revealing a well-ordered circular arrangement of GO sheets (Fig. 7g, h).

**Fig. 8:**
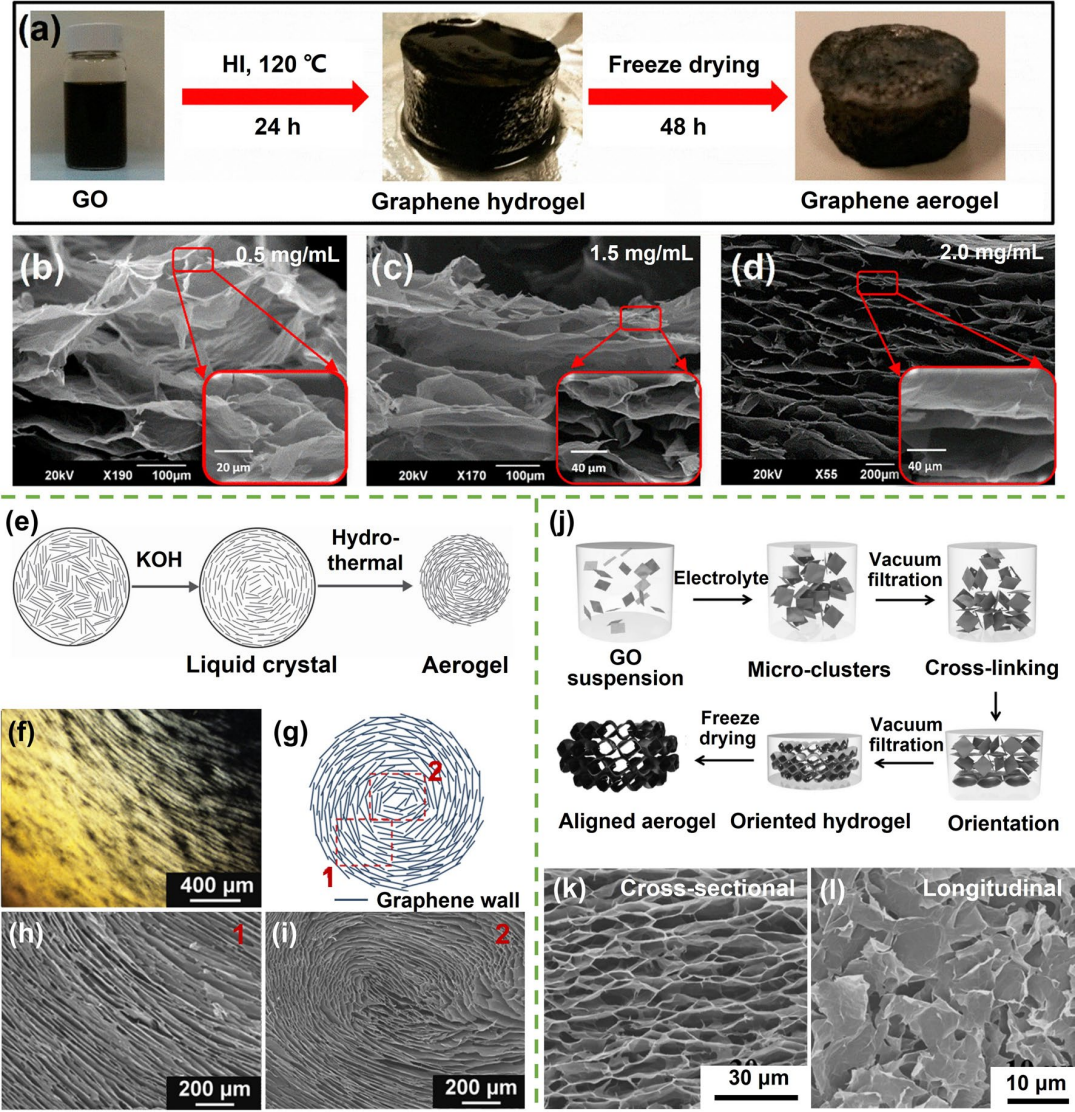
Typical self-assembly strategies for the fabrication of highly aligned graphene aerogels. **a** Hydrothermal treatment induced self-assembly of GO sheets for the fabrication of graphene aerogels and **b–d** corresponding aerogel morphologies fabricating using different GO concentrations. Reproduced with permission [11]. Copyright 2015, American Chemical Society. **e–i** Self-assembly of base-induced highly ordered GO LCs and microscopic structures of the LC and aerogel. Reproduced with permission [28]. Copyright 2015, Wiley–VCH. **j–l** Vacuum filtration- and crosslinking-assisted self-assembly. Reproduced with permission [123]. Copyright 2017, Wiley–VCH

Typical self-assembly process of utilizing the in-situ gelation of the ordered GO LCs by hydrothermal treatment to maintain the alignment characteristics. This step involves sealing the GO dispersion within a high-pressure vessel and heating it under controlled conditions, typically at temperatures ranging from 80 to 200 °C [114]. The elevated temperature and pressure create an environment that promotes the reconfiguration and reduction of oxygen functional groups, as well as partial restoration of the *sp*^*2*^ carbon–carbon bonding network in the GO sheets [115]. The hydrothermal treatment also facilitates the self-assembly of rGO sheets into three-dimensional structures due to the reformation of π-π stacking interactions, resulting in the formation of rGO hydrogels (Fig. 8a) [11, 116]. The inherent alignment of LCs remains preserved throughout the hydrothermal process due to the absence of any disruptive factors such as vibration or stirring during the self-assembly process [117, 118]. This natural preservation of order translates into graphene alignment within the resultant aerogels after freeze drying, as shown in Fig. 8b, c [119, 120].

The alignment achieved in self-assembled aerogels is intricately linked to the inherent orientation of GO LC precursors [116]. As such, factors influencing the LC order, such as the previously discussed concentration and lateral size of GO sheets, play a critical role in determining the eventual alignment of graphene sheets within the aerogels. Appropriate concentration of GO dispersion is crucial for fostering aligned microstructures during the hydrothermal self-assembly process. Insufficient concentrations lead to the absence of LC characteristics, while excessively high concentrations result in limited GO sheet mobility, hampering ordered arrangements (Fig. 8b–d) [11, 121]. Greater lateral dimensions or larger aspect ratios of GO sheets significantly enhance their anisotropic attributes, thereby being advantageous for achieving self-assembled alignment.

When employing conventional-sized GO sheets with an average size < 10 µm, the establishment of a greatly oriented LC needs a large concentration of ~ 10 mg mL^−1^ [112]. In the pursuit of lightweight materials, or more precisely, materials with elevated specific properties when normalized by density, the creation of ordered structures within GO suspensions at lower concentrations becomes imperative. A base-induced rearrangement of GO sheets within suspensions to form highly oriented LC structures at lower concentrations has been developed. Yao et al. potassium hydroxide (KOH)-induced evolution of GO liquid crystals (3.5 mg mL^−1^) with notably augmented ordering upon the addition of specific amounts of KOH, as shown in Fig. 8e [28]. Besides the KOH, the strong base sodium hydroxide was also proved to be effective in creating ordered laminar textures in GO LCs [122]. This evolution is a result of two synergistic factors: firstly, partial reduction of GO sheets by KOH extends their rigid domains, facilitating the creation of highly ordered microstructures; secondly, increased electrostatic repulsion between GO sheets enhances suspension fluidity, allowing GO sheets to migrate to regions of lowest Gibbs free energy [28, 122]. The impressive alignment achieved by KOH-induced ordered LCs (Fig. 8f) is seamlessly transferred to the self-assembly process, yielding graphene aerogels with analogous orientational morphologies (Fig. 8g–i).

The incorporation of electrolytes to disrupt the mutual balance of GO sheets within a solution has emerged as an effective strategy for achieving the self-assembly of graphene aerogels by overcoming electrostatic repulsion [124]. The introduction of specific amounts of electrolytes, such as acids [125], salts [126], and organics [124], triggers the formation of GO hydrogels. The electrolyte-induced assembly shares a common characteristic with other self-assembly methods, namely, that the alignment within the resulting graphene aerogels is profoundly influenced by the properties of GO LCs. In order to further enhance the alignment based on conventional GO LCs, the integration of vacuum filtration, which generates shear forces, with the electrolyte-induced assembly has been devised, as illustrated in Fig. 8j [123]. This innovative approach disrupts the electrostatic equilibrium between GO sheets through the introduction of electrolytes, leading to the formation of GO microclusters. Simultaneously, the application of vacuum filtration-driven external forces is harnessed to intensify alignment, thereby fostering the development of a finely tuned and ordered structure (Fig. 8k, l).

### Shear-Induced Alignment

**Fig. 9:**
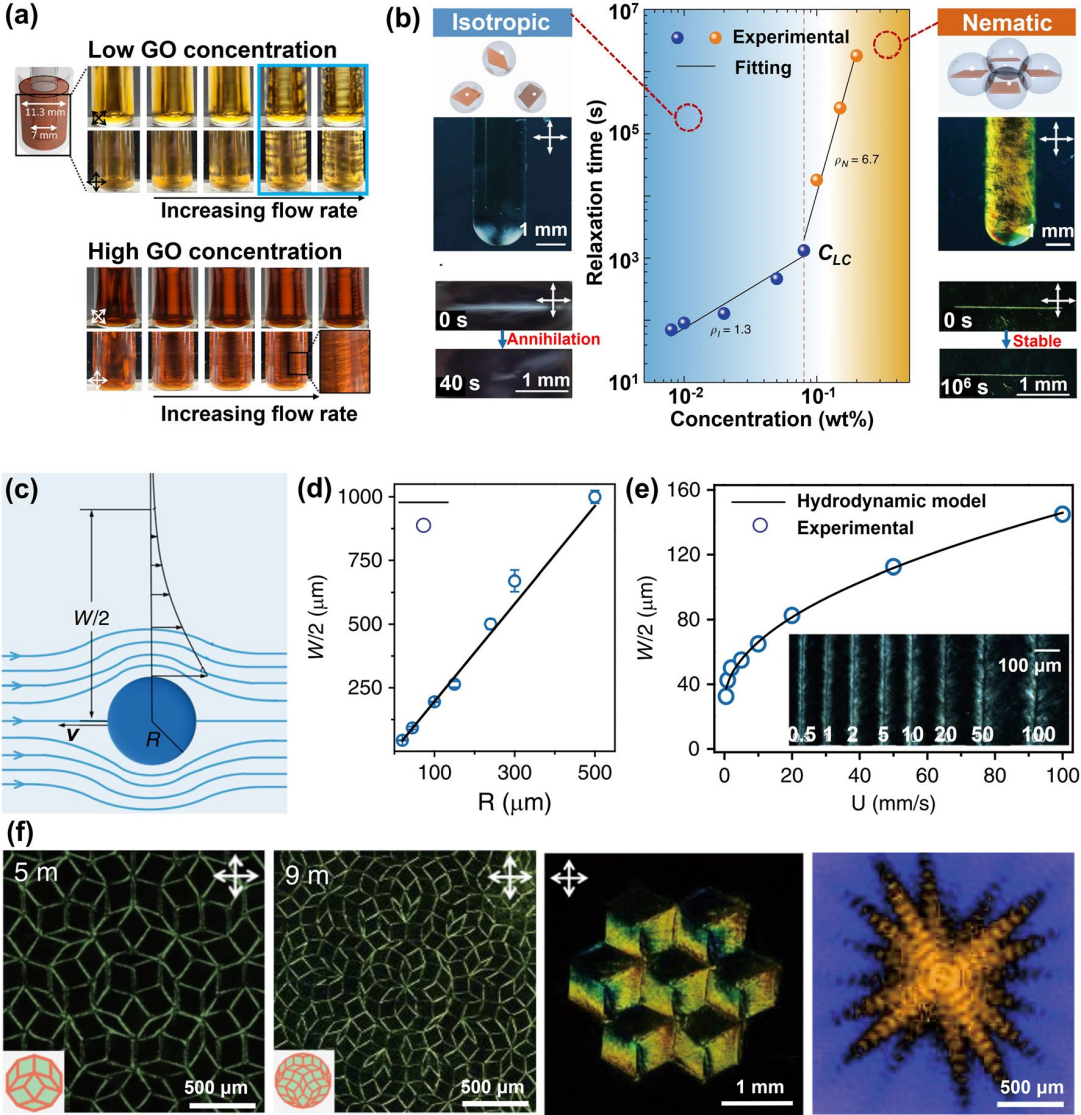
Shearing-induced GO rearrangement in LCs. **a** Macroscopic observation of GO suspensions under shearing. Reproduced with permission [129]. Copyright 2021, American Chemical Society. **b** Relaxation of GO LCs after scratching and **c-e** corresponding mechanisms revealing; and **f** patterned GO LCs constructed by shearing microlithography. Reproduced under the terms of the Creative Commons CC BY license [131]. Copyright 2019, The Authors, published by Springer Nature

GO dispersions exhibit shear-thinning behavior, characterized by a reduction in viscosity when subjected to shear stress [127]. This phenomenon arises from the realignment and reorientation of GO sheets, causing the disruption of interactions between the sheets. Initially, the presence of randomly dispersed GO sheets leads to a high viscosity due to their mutual interactions. However, the shear stress prompts the GO sheets to align and reorient, decreasing the resistance to flow and subsequently reducing viscosity [127]. The network-like structure formed by GO sheets, combined with their high aspect ratio and functional groups, contributes to the shear-thinning behavior. Upon shearing, GO suspensions can transition from a colloidal isotropic state to a nematic liquid crystal phase [128]. When the concentration of GO dispersion falls below the threshold for biphasic-to-nematic transition, the shear rate-dependent GO structure manifests non-Newtonian properties. At low shear rates, the low-concentration GO suspension displays weak shear-thinning behavior, while a shear-thickening behavior becomes apparent at high shear rates, as exemplified by the Taylor vortex flow in Fig. 9a [129]. In cases of high GO concentration, the more distinguished shear thinning results in the emergence of distinct stripe patterns as shear rates increase, ultimately leading to the formation of highly oriented GO liquid crystals (Fig. 9a).

The shear-thinning properties of GO liquid crystals enable the reconfiguration of GO sheets within the liquid crystal phase. Utilizing mechanical shearing by means of a rod or wire to scratch GO liquid crystals induces the reorientation of GO sheets in the direction of the shear [130]. Over a certain period, this reorientation may gradually subside, with the relaxation time directly correlated to the concentration of GO, as shown in Fig. 9b [131]. In the isotropic phase at lower GO concentrations, GO sheets exhibit a random distribution and engage in independent oscillations near their equilibrium positions, following a Brownian rotational motion. Following a single shearing event, relaxation occurs within tens of seconds. Conversely, the alignment of GO sheets achieved through shearing within the nematic liquid crystal phase remains stable for over 30 days, which suggests that a concentration larger than the *C*_*LC*_ is required for the fabrication of highly aligned graphene aerogels via shearing-induced alignment.

The mechanism underlying the shear-induced reorientation of GO sheets is elucidated in Fig. 9c–e [131]. As a rod moves within GO suspensions, it engenders a flow-around-pole phenomenon [132], giving rise to a shear field surrounding the in-motion rod, as shown in Fig. 9c. The width (*W*) of these localized Stokes flows is related to the rod’s radius and its moving velocity (*v*), and this relationship can be estimated using:

2 $$W=2\left(1+\sqrt{\frac{v}{{v}_{cr}}}\right)R$$

where the *v*_*cr*_ is the critical velocity to mobilize GO sheets. As the rod radius increases or the moving speed intensifies, the width of the reoriented GO suspensions expands (Fig. 9d, e). A GO LC possessing a viscosity of ~ 100 Pa·s and a density of ~ 10^3^ kg m^−3^ was employed to visualize the intriguing phenomenon of shear-induced GO sheet rearrangement. Through scratching the tape-casted GO LCs, where the GO sheets are predominantly aligned parallel to the plane, vertical alignment of GO sheets formed. This intriguing observation, depicted by the highlighted regions in Fig. 9f, holds the potential for practical applications in engineering the conformational aspects within LCs.

**Fig. 10:**
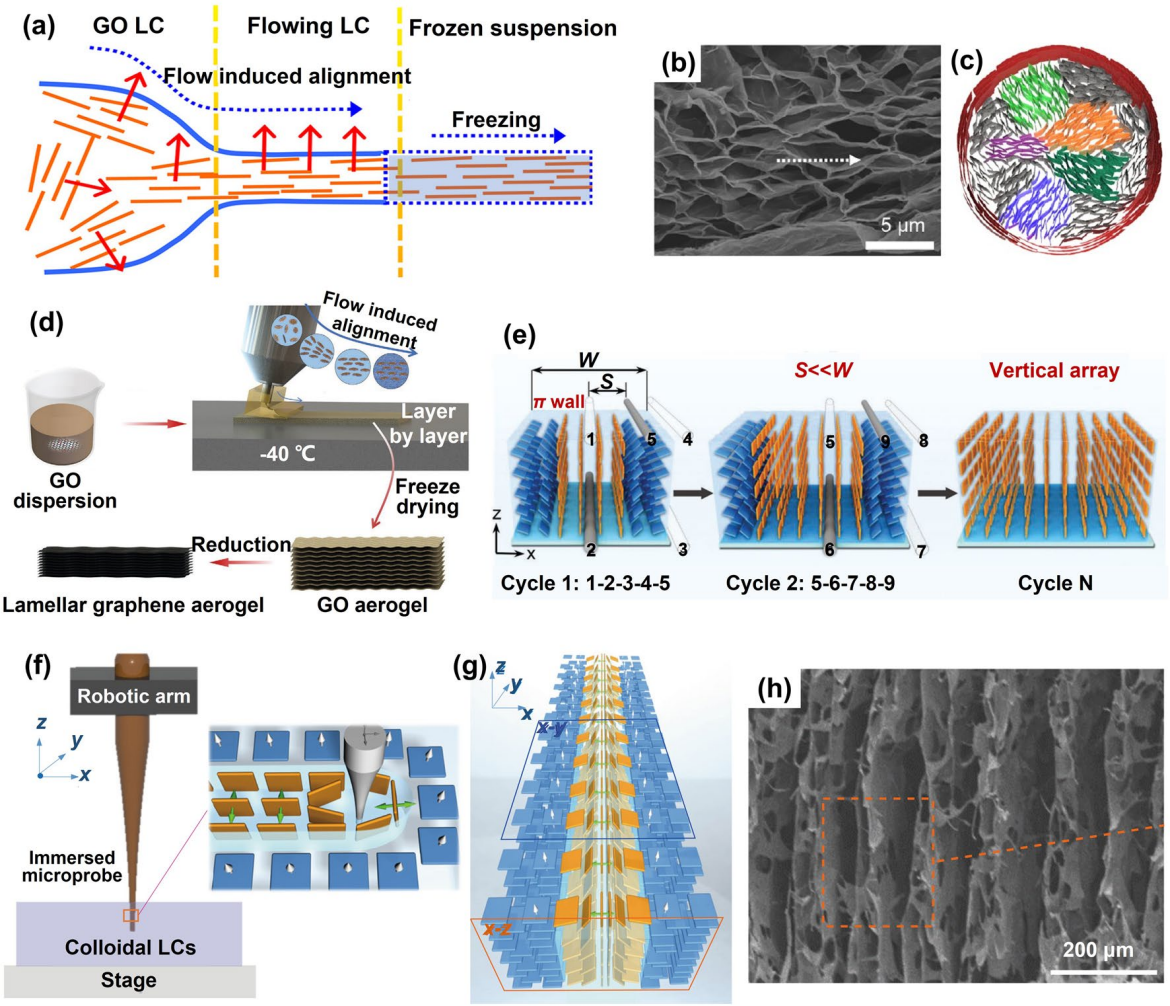
Typical shear-induced alignment for fabricating aligned graphene aerogels. Flow induced alignment: **a–c** Alignment during ejecting from a nozzle Reproduced with permission [55, 133]. Copyright 2012, 2014, American Chemical Society; and **d** Layer-by-layer 3D printing through a slit extrusion head Reproduced with permission [29]. Copyright 2022, Wiley–VCH. Shearing microlithography: **e** microwire shearing. Reproduced with permission [137]. Copyright 2023, Wiley–VCH; and **f–h** scratching the GO LC using an immersed microprobe Reproduced with permission [131]. Copyright 2019, The Authors, published by Springer Nature

On the basis of shear-thinning phenomenon and shear induced alignment of GO suspensions, two typical aligning techniques have been extended for the fabrication of highly aligned graphene aerogels, namely the flow induced alignment (Fig. 10a–d) and the shearing microlithography (Fig. 10e–h). The technique of uniaxial flow has been widely embraced to induce the alignment of GO sheets along a specific flow direction. This approach, when combined with a subsequent freeze-casting process, unveils a promising avenue for crafting graphene aerogels with exceptionally high alignment. The general procedure involves loading GO LCs into a syringe, followed by controlled extrusion through a nozzle at a carefully regulated speed into a frigid bath. Notably, the aligned GO sheets within the LCs come to the fore at the nozzle region and steadfastly maintain their alignment as they freeze in the cold bath, as vividly illustrated in Fig. 10a [133]. Following the subsequent freeze-drying step, this resulting aerogel manifests a morphology akin to that achieved through unidirectional freeze casting techniques. As shown in Fig. 10b, c [55], the material showcases aligned graphene sheets running parallel to the flow direction, while the cross-section reveals a multi-domain tubular structure.

The flow-induced alignment can also be combined with 3D printing to fabricate carefully designed hierarchical graphene aerogels. A case in point is the work by Wang et al., who achieved the fabrication of a high-density graphene aerogel microlattice with an ordered structure through the direct ink writing of glycerol-functionalized GO LCs [134]. They yielded a striking outcome with highly aligned filaments within the structure. Furthermore, when employing a slit extrusion head, the potential of producing planar graphene aerogels with horizontally aligned building blocks becomes feasible. This was achieved through a layer-by-layer deposition of GO dispersions on a cooled platform, followed by freeze-drying, as illustrated in Fig. 10d [29]. It's noteworthy that the specific morphology of the printed graphene aerogel is intricately intertwined with the printing speed and the diameter of the slit extrusion, imparting a tunable quality to the process.

The method of shearing microlithography entails the movement of a stick within the GO LCs, a process that facilitates the emergence of macroscopically aligned two-dimensional (2D) GO nanosheets. The outcome of this procedure is visually striking, revealing distinct dark and bright textures that represent the anisotropic optical responses of the meticulously ordered GO nanoflakes [135]. The larger GO sheets, boasting a greater aspect ratio, exhibit a more pronounced moment of force under the influence of the shearing field [108]. This engenders a larger driving force, enabling them to overcome the resistive hydrodynamic drag within the viscous suspension [136]. In a similar vein, the orientation of graphite oxide suspensions can also be meticulously controlled through shearing, serving as a valuable reference for aligning GO. A boundary-free vertically-moving microwire shearing technique has been implemented to achieve a precise vertical orientation of colossal graphite oxide sheets, as vividly depicted in Fig. 10e [137]. Large-scale vertical arrays of giant graphite oxide flakes were fabricated by narrowing the interval spacing (*S*) between adjacent shearing fields. The initial cycle of this process resulted in a π wall, signifying a distinct nonuniform structure marked by vertical alignment within the central domain transitioning to horizontal alignment at the boundaries, where the width (*W*) spanned 50 µm [137]. With a favorable *S*/*W* ratio of 0.1, a remarkably well-aligned porous microstructure emerged, displaying an intriguing anisotropic skeleton with bidirectional sheet ordering.

Vertical alignment of GO sheets can be effectively achieved through the horizontal sliding of a probe immersed within the GO suspension. This technique allows for the digital programming of GO LCs, offering the capability to program the alignment or texture of GO sheets. Different moving routines of the microprobe can be employed to achieve precisely programmed alignment patterns [138]. In cases where the probe is guided towards a specific direction within a sufficiently small interval, a uniform and distinctly vertical alignment of GO sheets is realized, as schematically illustrated in Fig. 10f, g [131]. Through a meticulous freeze-drying process, this vertical alignment phenomenon becomes an inherent trait of the resultant graphene aerogel, as exemplified in Fig. 10h.

### Further Enhancement of Alignment

**Fig. 11:**
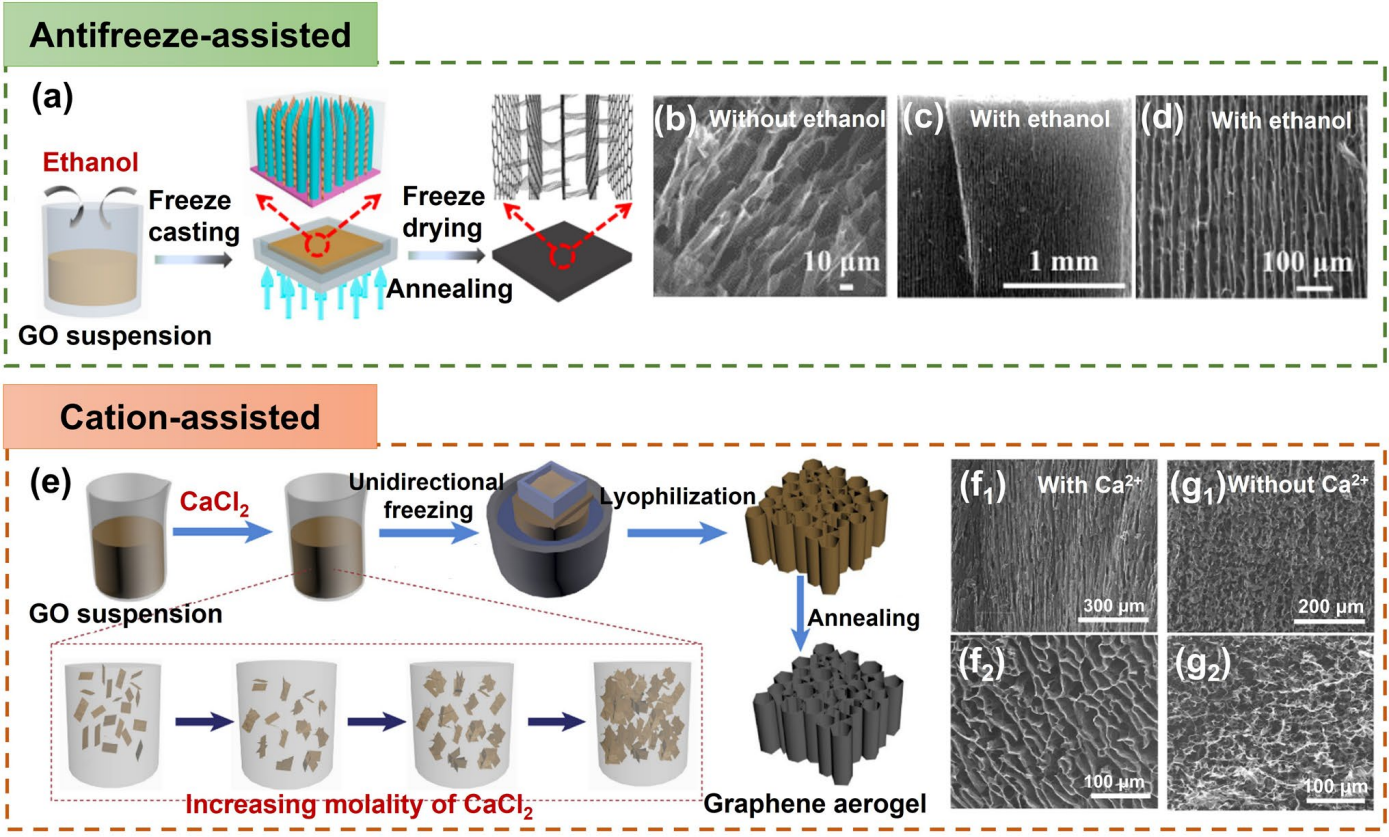
Further enhancement of alignment in graphene aerogels. **a–d** The antifreeze-assisted aligning. Reproduced with permission [30]. Copyright 2017, American Chemical Society. **e–g** Cation-facilitated alignment. Reproduced with permission [35]. Copyright 2021, Elsevier

While GO presents a promising foundation for the construction of aligned graphene aerogels, its extensive oxygen-containing groups, particularly hydroxy groups, situated on the basal plane of GO, lead to the creation of more hydrogen bonds with ice crystals than with liquid water. This intricate interaction inhibits the growth of ice crystals [139], consequently impeding the alignment in resulting aerogels even under the conditions of unidirectional or bidirectional freeze casting. Efforts have been made to mitigate the interactions between GO sheets and ice crystals that form in situ, aiming to enhance alignment within GO architectures, including, (i) antifreeze-assisted freezing [30], (ii) cation-assisted freezing [35], and (iii) additional thawing and freezing [140] (Fig. 11).

Organic solvents, widely recognized as potent antifreeze agents, exert a notable influence on the freezing dynamics, altering the crystallization patterns of ice crystals via their interaction with hydrogen bonds [141]. This phenomenon of antifreeze-induced ice growth inhibition, coupled with the orchestrated process of directional freeze casting, manifests in the emergence of vertically aligned graphene sheets. The antifreeze-assisted freezing technique was achieved through the judicious introduction of small quantities of solvents, such as ethanol, methanol, and acetone, into GO suspensions prior to unidirectional freeze casting (Fig. 11a) [30]. Among these candidates, ethanol is particularly effective, yielding long-range ordering throughout the sample. Evidently, the alignment of GO sheets under the influence of ethanol exhibited a remarkable enhancement in comparison to those prepared without ethanol (Fig. 11b–d) [30]. The ethylene glycol [142], tetrahydrofuran [143], and even NH_4_OH [144] have also been demonstrated as effective antifreezes for orchestrating ordered structural formations within GO scaffolds, ushering in a nacre-inspired lamellar arrangement.

Metallic cation ions, such as Ca^2+^ and Mg^2+^, demonstrate a remarkable capacity to engage in coordination with the hydroxyl and carboxyl groups presented on GO sheets [145], resulting in the interconnection of adjacent GO sheets and a subsequent increase in the viscosities of GO dispersions and culminating in the formation of robust gels [146, 147]. A Ca^2+^-assisted unidirectional freezing technique is effective for the fabrication of graphene aerogels characterized by a heightened degree of anisotropy (Fig. 11e) [35]. The reduced oxygen-containing groups in GO sheets after Ca^2+^ coordinating is proposed to attenuate the interactions between GO sheets and the ice crystals formed in situ during the freezing process, which contributes to the efficient expulsion of GO building blocks through ice pillars during the directional-freezing sequence. Graphene aerogels, cast unidirectionally utilizing crosslinked GO suspensions at an optimal Ca^2+^ concentration, exhibit an exceptional degree of order within their graphene architectures (Fig. 11f1, f2). This pronounced alignment stands in stark contrast to their counterparts fashioned from unaltered GO dispersions, devoid of crosslinking (Fig. 11g1, g2).

The freeze-thaw assembly strategy encompasses a sequence of chemical prereduction, freeze-thaw, further reduction, and freeze-drying processes [148, 149]. Within this routine, the prereduction step assumes a pivotal role in shaping the ultimate pore architecture of rGO aerogels. This influence emanates from the prereduction degree's impact on the abundance of oxygen-containing groups present in GO sheets, consequently dictating the interconnection and gelation tendencies of GO [150]. The architectures of rGO aerogels originating from partially reduced GO microgels are subsequently modulated during the ensuing freeze-thaw phase. Central to this is the fine-tuning of inter-sheet π-π interactions during the supplementary thawing and reduction phases. This optimization confers a robust structural framework that withstands the additional ice growth process [151]. Through subsequent cycles of thawing and freezing, the arrangement of rGO sheets is further refined, culminating in heightened alignment of constituent building blocks and the realization of highly oriented graphene aerogels [149].

### Comparison of the Aligning Techniques

Based on the above discussion regarding the fabrication of aligned graphene aerogels, a comparative overview is summarized in Table 1. Driving forces of the directional freeze casting, self-assembly and shear-induced alignment are the growth of ice crystals, reduction-induced gelation, and shearing, respectively. Morphologies of graphene aerogel fabricated by directional freeze casting is highly related to the direction of temperature gradient, whereas self-assembly and shearing induced alignment is dependent on the orientation of GO LCs and the shearing direction, respectively (Fig. 2). Lamellar structure can be obtained from the bidirectional freeze casting and shearing forced aligning. Long-range alignment is easy to be obtained by techniques except self-assembly induced aligning. Owing to their highly aligned lamellar structure and remarkable superelasticity, bidirectionally freeze-cast graphene aerogels find particular applicability in pressure sensing and thermal management applications. Meanwhile, the radial alignment in radially freeze-cast aerogels affords ample surrounding channels for the movement of organic solvents and oils, making them advantageous for organic absorption applications.

**Table 1:** Comparisons of different aligning techniques for graphene aerogels

Aligning techniques	Directional freeze casting			Self-assembly	Shearing forced alignment
	Unidirectional	Bidirectional	Radial		
Driving force	Growth of ice templates			Reduction-induced gelation	Shear force
Alignment	Unidirectional	Bidirectional	Radial	Inherent from GO LCs	Along shearing direction
Morphologies	Tubular	Lamellar	Radially lamellar	Multi-domain alignment	Tubular or lamellar
Advantages	Long-range aligned; precise control over alignment; scalable			Easy-processing; versatile	Scalable; large-size; patternable alignment
Shortcomings	Careful optimization of freezing parameters is required			Limit in long-range alignment; less anisotropic	High-concentration GO LC is required
Advantageous applications	Electrochemical; Organic absorption	Superelasticity related (*i.e.* pressure sensing); thermal management	Organic absorption	Electrochemical	Thermal management
Relative fabrication cost	Relatively low			Medium	High

The costs of different fabrication techniques provide important information for industries manufacturing. The relative fabrication cost of the aligning techniques discussed in this review are summarized in Table 1. The directional freeze casting method involves a freezing process using cold sources, such as liquid nitrogen or chilly ethanol. The freezing period is relatively short because of the ultralow-temperature cold source, taking about tens of minutes. The design of the freezing mold, generally made by polymers and metals, and the temperature gradient are the key to control the direction of ice growth. With the mold and cold source being main expenses of the directional freeze-casting (no complicated equipment), the easy-processing freeze casting technique are relatively cheap and time-efficient, being the most promising aligning technique for large-scale applications [26, 152]. However, careful optimization of freezing parameters is required for long-range alignment. The self-assembly aligning technique involves reduction induced gelation of GO LCs, generally a hydrothermal process at temperature ranges of 60–200 °C for about a few hours to over 1 day [28, 53, 126]. Taking the electronic energy and time cost into account, the cost for self-assembly method lies in-between the directional freeze casting and shear induced aligning. The shear-induced aligning techniques, including flow induced aligning and shearing microlithography of GO LCs, are promising for large-scale fabrication of graphene aerogels with long-range alignment, but the cost of equipment for large aerogels are relatively high. Besides, large-size, high-concentration GO LCs are required, increasing the cost of raw material and make this technique the most expensive among the three aligning techniques [29, 137, 153]. While the freeze casting method demonstrates scalability and a cost advantage compared to other aligning techniques, the overall cost of the graphene aerogel fabrication process remains relatively high for large-scale industrial applications. This mainly includes expenses associated with the costly GO suspension, freeze casting, freeze drying, and post-treatments (such as chemical reduction and high-temperature annealing), limiting practical applications of graphene aerogels.

### Fabrication of Highly Aligned Graphene Aerogel/Polymer Composites

**Fig. 12:**
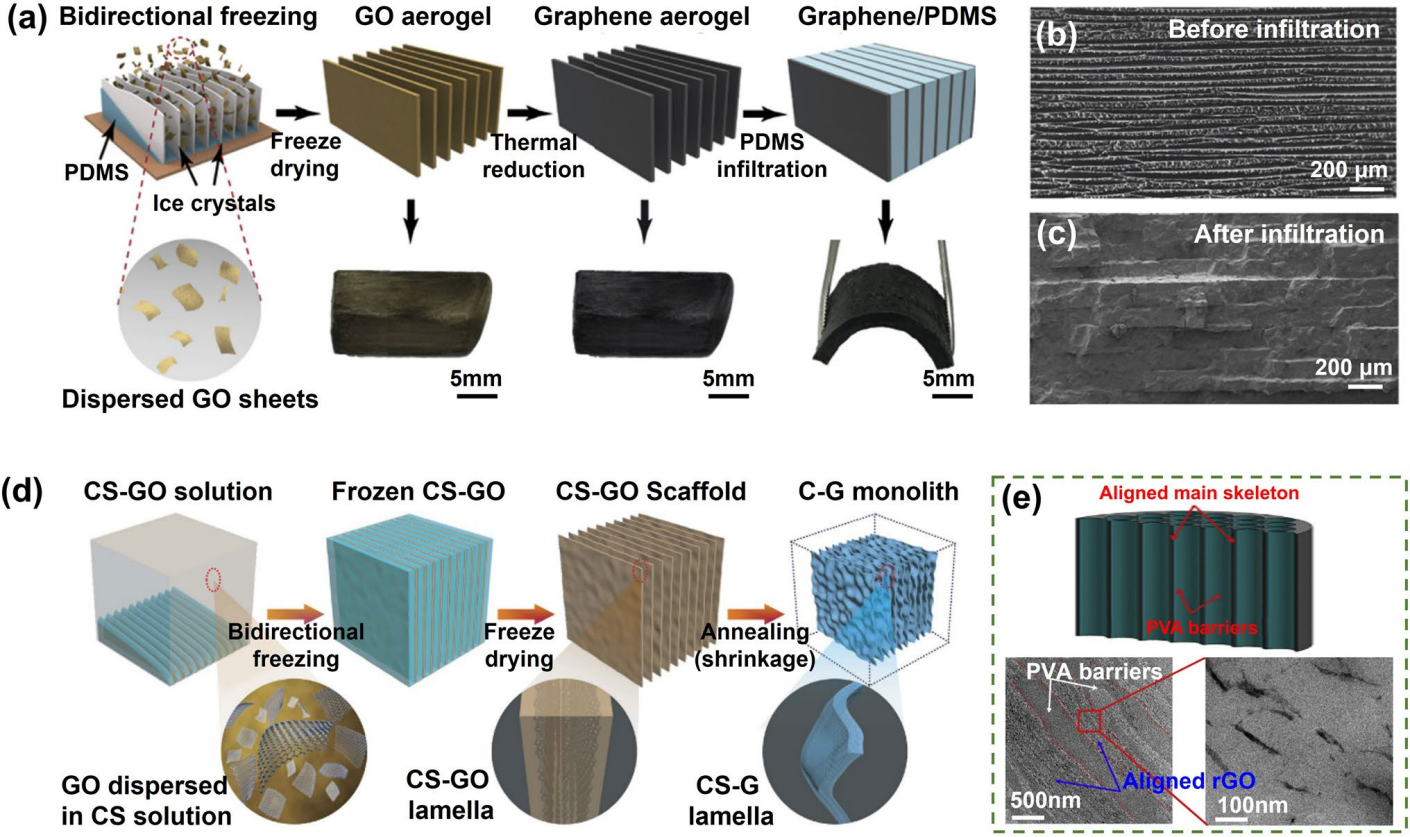
Fabrication of aligned graphene aerogel-based composites via **a–c** post-infiltration of polymer matrix in the preconstructed aligned graphene aerogels. Reproduced with permission [154]. Copyright 2019, Elsevier; and **d** directional freeze-drying of GO/polymer suspensions. Reproduced under the terms of the Creative Commons CC BY license [156]. Copyright 2016, The Authors, published by Springer Nature. **e** Nanoscopic alignment of graphene sheets in the aligned composite skeleton. Reproduced with permission [157]. Copyright 2017, Elsevier

One approach for fabricating aligned graphene aerogel/polymer composites involves constructing highly aligned graphene aerogels followed by polymer infiltration, as shown in Fig. 12a [154]. In this methodology, the foundation lies in the fabrication of highly aligned graphene aerogels, a task achieved through diverse means such as template-based methodologies, self-assembly, or precision alignment techniques like shearing. These strategies confer meticulous command over the spatial orientation of graphene within the aerogel's intricate architecture. Once the coveted highly aligned graphene aerogels are realized, they are subsequently infused with a polymer solution or precursor, facilitating the complete impregnation of the polymer matrix into the porous aerogel framework. This meticulous infiltration ensures an intimate interlocking between the polymer and the pre-existing aligned graphene scaffold. Subsequently, the solvent is extracted or the polymer is cured, obtaining an aligned graphene/polymer composite [143]. Importantly, post-polymer infiltration, the profound orientation of graphene walls within the aerogel remains impeccably preserved, as shown in Fig. 12b, c. The resultant composite material showcases significantly enhanced mechanical, electrical, and thermal attributes, rendering it a versatile contender for a spectrum of applications [155].

An alternative avenue for crafting aligned graphene /polymer composites aerogel involves the technique of directional casting of graphene/polymer solutions, as shown in Fig. 12d. This method entails the preparation of a graphene/polymer solution, achieved by uniformly dispersing graphene within a polymer solution to obtain a homogenous solution or suspension, typically an aqueous medium. The ensuing step encompasses the casting or pouring of this solution into a designated mold, subsequently subjecting it to the aligning procedures elucidated earlier. Following the freeze-drying phase, the resultant composite aerogel embodies the framework of aligned graphene/polymer composite walls [156]. Notably, these aligned graphene/polymer composite aerogels can also be subjected to external compressive forces for the fabrication of composites, ultimately culminating in the formation of solid composites furnished with oriented graphene fillers (Fig. 12e) [157].

Overall, both methodologies present potent avenues for the creation of aligned graphene aerogel/polymer composites, each proffering distinct merit. The first approach centers on the meticulous fabrication of highly aligned graphene aerogels, followed by polymer impregnation, obtaining aligned 3D interconnected graphene aerogel networks within composites [143]. Conversely, the second technique employs the directed casting of graphene/polymer solutions, promoting the simplicity and scalability. Uniformly dispersed and aligned graphene sheets in the aligned skeletons are generally obtained. The ultimate choice between these methods relies on factors such as the desired level of alignment control, scalability, and the specific requisites of the intended application [158].

## Fundamental Properties

### Anisotropic Properties

**Fig. 13:**
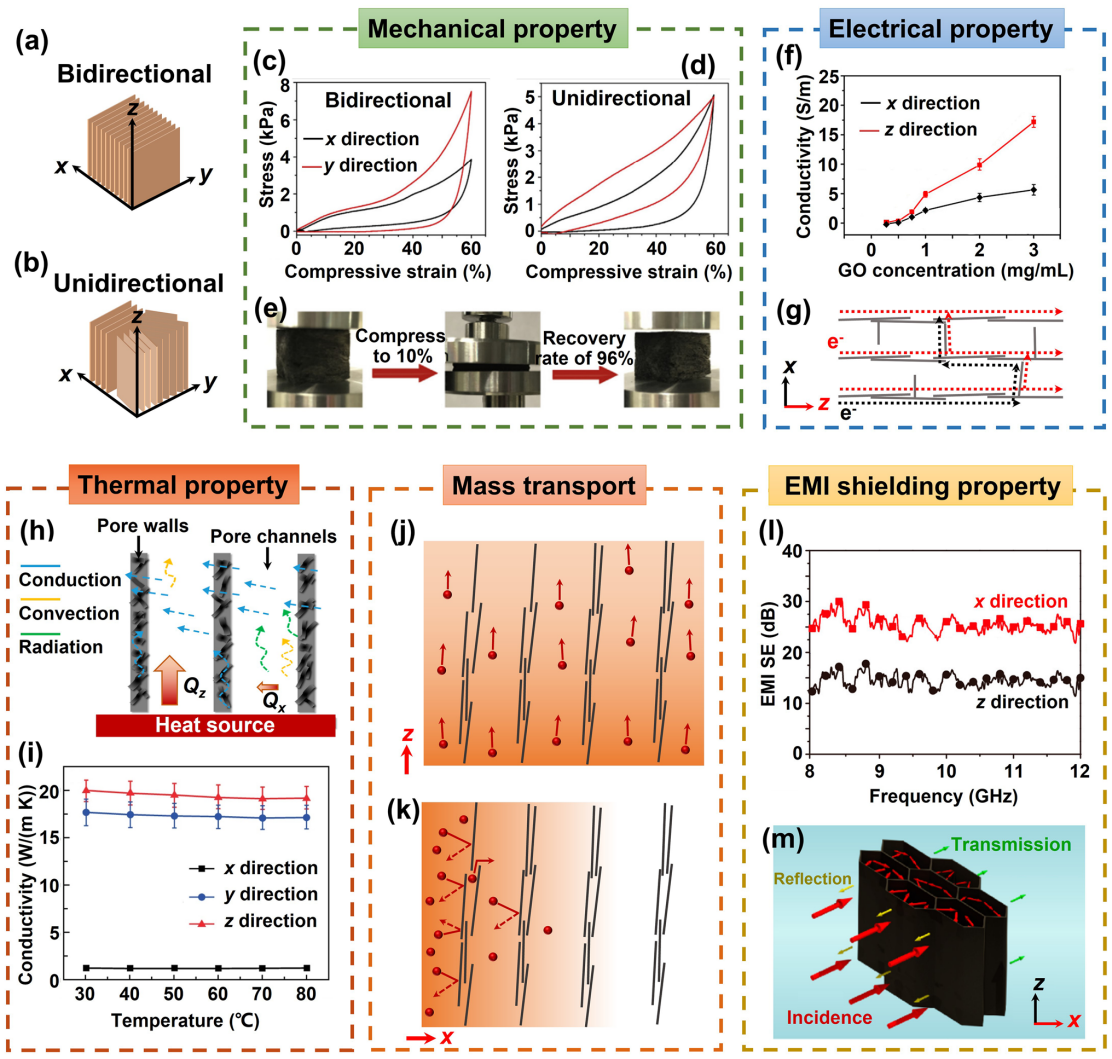
Anisotropic properties of highly aligned graphene aerogels and their composites. **a, b** Illustrations of the coordinate directions. Anisotropic properties and relevant mechanism: **c–e** mechanical. Reproduced with permission [33]. Copyright 2018, Elsevier. **f, g** Electrical. Reproduced with permission [5]. Copyright 2016, American Chemical Society. **h, i** Thermal. Reproduced with permission [34]. Copyright 2022, American Chemical Society. Reproduced under the terms of the Creative Commons CC BY license [166]. Copyright 2020, The Authors, published by Springer Nature. **j, k** Mass transport. **l, m** EMI shielding properties. Reproduced with permission [171]. Copyright 2016, American Chemical Society

The carbon atoms constituting the graphene lattice are intricately linked through robust covalent bonds, forming a resilient 2D structural framework. Due to the atomic structure and bonding, graphene exhibits intrinsically anisotropic properties, which lead to anisotropy in highly aligned graphene aerogels. The directional dependence of graphene's properties extends to the aerogel structure when graphene sheets are aligned within it. This directional dependency inherent to graphene's properties becomes entwined with the aerogel's characteristics, thereby imparting direction-specific qualities. As a consequence of the alignment, the aerogel's mechanical, electrical, thermal, transport, EMI shielding, and other properties are significantly influenced along different orientations. For the sake of clarity in our discussion, the directional aspects of properties are graphically represented in Fig. 13a, b.

The anisotropy of mechanical stiffness and hysteresis of aligned graphene aerogel and their composites have been demonstrated by researchers [58, 89]. In bidirectionally freeze-dried graphene aerogel, the alignment of graphene creates a continuous load-bearing network, enabling efficient stress transfer and resulting in higher stiffness along the aligned direction (the *y* direction), as shown in Fig. 13c [33]. Conversely, the absence of a continuous alignment perpendicular to the aligned direction (the *x* direction) leads to lower stiffness. As a comparison, the unidirectionally freeze-dried graphene aerogels show similar stiffnesses in the *x* and *y* directions (Fig. 13d), both being subordinate to that observed in the *z* direction [33]. This is due to the isotropic porous structure within the *xy* plane and the alignment of skeleton along the *y* direction [159]. The comparison of mechanical properties between bidirectionally and unidirectionally freeze-dried graphene aerogel confirms the contributions of graphene alignment to macroscopic properties. Hysteresis, indicative of energy dissipation during cyclic loading and unloading, generally exhibits lower values perpendicular to the alignment, predominantly because of weaker frictions among graphene sheets and gentler airflow within the porous structure during deformation [37, 58]. Consequently, highly aligned graphene aerogels show super-elasticity exceptional and resilience upon cyclic compression [160–162]. An example is that the bidirectional freeze-casting graphene aerogels a 96% recovery of height after a compression to 90%, as shown in Fig. 13e [33]. The anisotropic mechanical properties of aligned graphene aerogel can be inherited their composites, showing different stress–strain characteristics along different directions [154].

The delocalized π-electrons within the carbon hexagonal lattice enable electrons to move unrestrictedly along the graphene plane, rendering high electrical conductivity along its in-plane direction. This characteristic extends to graphene aerogels and their composites, where aligned graphene conductive frameworks give rise to anisotropic electrical conductivity, marked by enhanced conductivity along the aligned axis and diminished conductivity perpendicular to it [34]. An illustrative example of this phenomenon is observed in the research by Wang et al. who investigated anisotropic electrical conductivities in graphene aerogel freeze-dried from GO suspensions of varying concentrations, as shown in Fig. 13f [5]. The anisotropy of electrical conductivity in graphene aerogels becomes more pronounced with higher concentrations of GO, attributing to increased alignment of graphene sheets induced by the elevated GO concentration. Similarly, Gao et al. reported on highly aligned graphene aerogel/PDMS composites displaying electrical conductivity notably higher by 1–2 orders of magnitude in the *z* direction compared to the *x* direction [154]. The anisotropy in electrical conductivity is attributed to the aligned graphene sheets within the aerogel structure, which establish a continuous conductive network along the alignment direction and an inconsecutive bridging structure in the transverse direction, thus enabling more efficient electron transport and higher conductivity along the direction of alignment (Fig. 13g).

The interplay of convection, conduction, and radiation mechanisms significantly influences the anisotropic thermal conductivity observed in aligned graphene aerogels. The arrangement of graphene sheets within the aerogel creates an uninterrupted conductive network, facilitating efficient electron and phonon transport and consequently enhancing thermal conduction [34], as illustrated by the solid blue arrows in Fig. 13h. Notably, the thermal conduction occurring between the aligned graphene skeletons is markedly higher along the alignment direction [34], owing to the relatively lower thermal conductivity of air present among aligned graphene walls. Additionally, the occurrence of thermal convection perpendicular to the alignment is negligible due to the limited pore size among the graphene walls, which restricts the onset of natural thermal convection [163]. The distinctive anisotropic structure also enhances heat dissipation along the *z* direction, preventing heat accumulation [34]. Consequently, aligned graphene aerogels exhibit anisotropic thermal conductivities, showing remarkably higher thermal conductivity along the alignment direction compared to the perpendicular direction [37, 64, 159]. When graphene aerogel-based composites are formed by infiltrating polymers into the pre-constructed porous graphene architectures, the anisotropic thermal conducting traits of aligned graphene aerogels extend to these composites as well [164, 165]. An illustrative example by Liu et al. involves the fabrication of lamellar-like graphene aerogels through bidirectional freeze-casting, succeeded by compaction perpendicular to the alignment direction and vacuum-assisted impregnation with epoxy to produce composites featuring a high alignment of graphene fillers [166]. These composites demonstrate a notable anisotropy in thermal conductivity, exhibiting around 20 and 17.5 W m^−1^ K^−1^ along the *z*- and *y*- direction, respectively. However, the thermal conductivity decreases substantially to about 1.2 W m^−1^ K^−1^ along the *x*-direction, which is perpendicular to the alignment, as shown in Fig. 13i.

Graphene sheets possess exceptional barrier properties that effectively block the transport of matter through their plane, owing to the tightly packed atomic structure strong bonding energy, and unique electronic configuration of graphene [167]. These factors collectively create a robust barrier that prevents the passage of molecules or atoms through the graphene sheet [168]. Aligned graphene aerogels capitalize on this inherent barrier capability by featuring porous channels oriented along the alignment direction and stacked graphene walls in the transverse orientation. This structural arrangement gives rise to intriguing anisotropic transport characteristics, as illustrated in Fig. 13j, k. The aligned pores establish preferential pathways for the transport of molecules, ions, and substances, offering a conduit for efficient movement. On the contrary, the walls, composed of densely stacked graphene building blocks, exhibit significantly greater resistance, limiting the transport of substances perpendicular to the alignment. This leads to the distinct anisotropic transport properties observed in aligned graphene aerogels, where molecular or atomic movement becomes directionally dependent.

The EMI shielding of materials is attributed to reflection, absorption, and multiple reflection of electromagnetic waves [169]. The reflection of electromagnetic radiation is fundamentally a consequence of the interaction between waves and free charges present on the material's surface, which suggest that the presence of conductive networks characterized by a substantial concentration of charge carriers significantly contributes to the efficacy of reflection-based EMI shielding effectiveness (SE). The SE associated with absorption quantifies a material's capacity to attenuate electromagnetic radiations into thermal or internal energies, which is achieved through processes such as the generation of localized currents within conductive networks, the polarization or relaxation of dipoles and charges, and the charge delocalization [170]. An integral aspect that emerges from these mechanisms is the evident correlation between the EMI shielding properties of materials and their electrical conductivities. Therefore, the anisotropic electrical conductivity intrinsic to aligned graphene and its composites naturally gives rise to anisotropic EMI shielding properties (Fig. 13l) [82, 154, 171]. As electromagnetic radiation penetrates graphene aerogels from a direction perpendicular to the alignment, the waves experience a series of reflections and scatterings at the numerous interfaces presented by the oriented graphene walls. This continuous back-and-forth interaction contributes to the gradual dissipation of electromagnetic waves into thermal energy (Fig. 13m) [171]. But when the waves approach the aerogels parallel to the aligned graphene frameworks, their penetration is comparatively unhindered, leading to a reduction in the dissipation of incident radiations [34, 172].

### Contributions of Alignment to Physical Properties of Composites

#### Electrical Conductivity

**Fig. 14:**
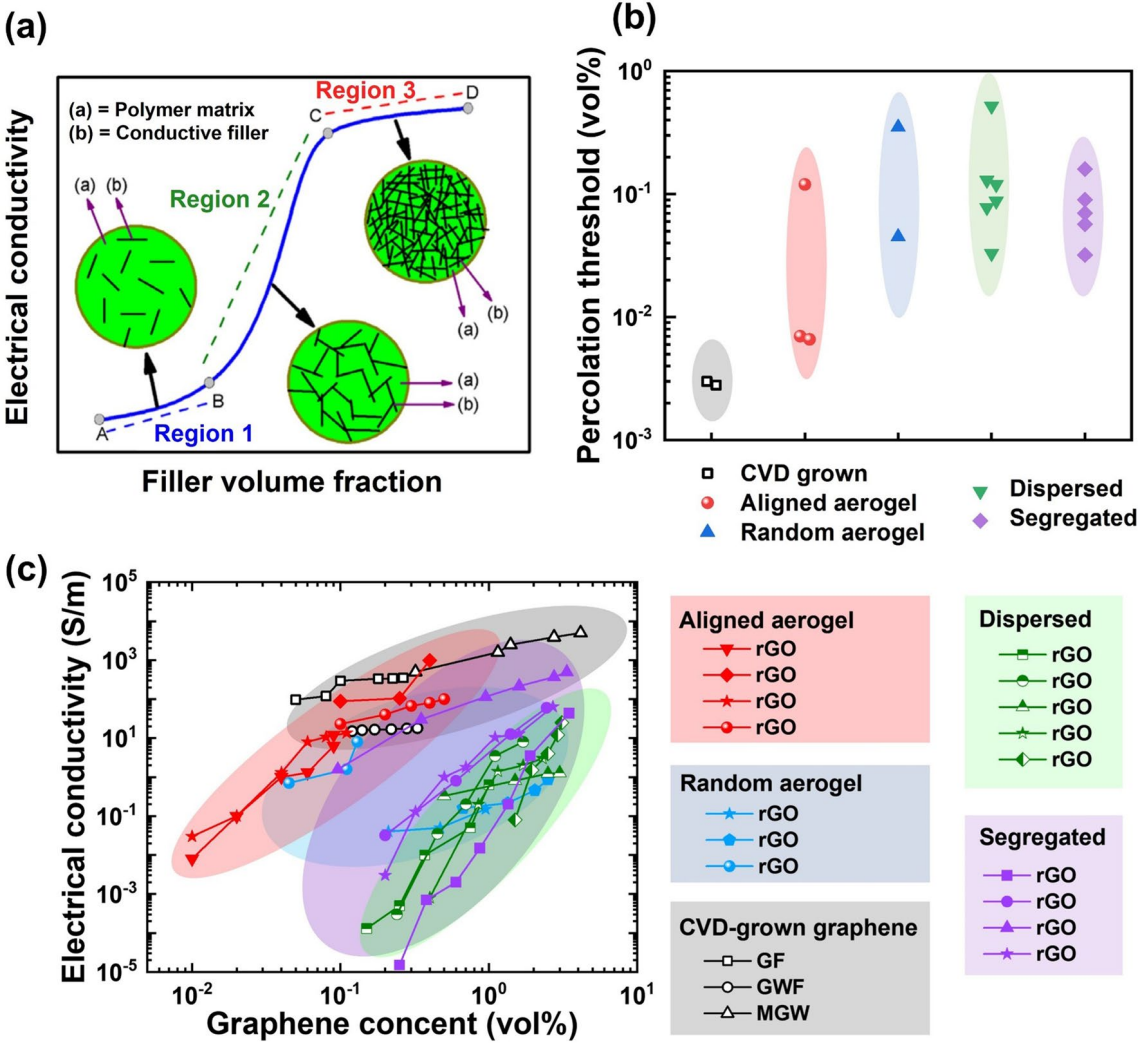
Electrical percolation and conductivity of graphene/polymer composites. **a** Schematic illustrations of the percolation theory. Reproduced with permission [193]. Copyright 2016, Society of Chemical Industry. **b** Percolation threshold of polymer composites reinforced with different fillers: CVD-grown graphene [176, 177], aligned graphene aerogel [5, 11, 65], random graphene aerogel [178, 179], dispersed graphene [180–185], and segregated graphene [186–189]. **c** Electrical conductivity of polymer composites reinforced with different fillers: aligned graphene aerogel [5, 65, 171, 190], random graphene aerogel [178, 191, 192], CVD-grown graphene network [177, 194, 195], dispersed graphene [180, 196–199], and segregated graphene [187–189, 200]

*Percolation Theory* Electrically conductive materials have diverse applications in electronics, energy storage, sensors, EMI shielding, wearables, automotive systems, and aerospace, emphasizing their essential role in modern technology. When conductive additives are introduced into insulating polymers, the electrical conductivity of the resulting composites undergoes a distinct transition from an insulating to a conducting state as the concentration of conductive components or pathways within the material surpasses a specific threshold, which is well-known as the percolation phenomenon [173]. In the region below this threshold, the composite material behaves as an insulator, displaying minimal to negligible electrical conductivity. However, once the threshold is exceeded, the material makes a pivotal shift into a conductive state, with electrical characteristics becoming exceptionally responsive to the concentration and arrangement of the conductive constituents, as shown in Fig. 14a [174].

The transition is often described as a critical connectivity point where a continuous conductive pathway forms across the material, allowing the flow of electric current. The percolation threshold is a specific concentration value above which the material's conductivity increases dramatically, which can be estimated based on the power law equation [175]:

3 $$\sigma \propto {\sigma }_{f}{(V-{V}_{p})}^{a}$$

where *σ* and *σ*_*f*_ are the electrical conductivity of composite and filler, *V* and *V*_*p*_ represent the filler volume fraction and the percolation threshold, and *a* is a critical exponent. In essence, the percolation phenomenon elucidates the intriguing interplay between the arrangement and concentration of conductive elements within a composite material, fundamentally influencing its electrical properties.

The percolation threshold of various graphene/polymer composites containing different networks of graphene are plotted in Fig. 14b. The composites reinforced with large-aspect-ratio chemical vapor deposition (CVD) grown graphene sheets characterized by a high aspect ratio exhibited remarkably low percolation thresholds, attributing to the pristine lattice structure and the extensive aspect ratio of these graphene sheets [176, 177]. Interestingly, pre-fabricated composites incorporating aligned graphene aerogels showcased exceptionally low percolation thresholds of approximately 0.007 vol% [5, 65]. This strikingly low value is primarily a result of the formation of conductive networks within the well-ordered walls of the aerogel architecture, achieved at an impressively modest filler content. However, an aligned graphene aerogel created through a hydrothermal-induced self-assembly process exhibited a comparatively higher percolation threshold of 0.12 vol% [11], resulting from the stacking of GO building blocks during the hydrothermal process. Comparatively, composites containing randomly oriented graphene aerogels exhibited notably higher percolation thresholds than those composed of aligned graphene aerogels [178, 179]. Composites consisting of random graphene aerogels showed relatively lower or comparable percolation thresholds than those with dispersed graphene sheets [180–185]. The segregated graphene reinforced composites [186–189], generally fabricated by hot compression of graphene coated polymer spheres, displayed percolation thresholds comparable to the random graphene aerogel reinforced composites. In a comprehensive assessment of various graphene network types within polymer composites, it is evident that the aligned aerogel architecture emerges as a promising option.

*Electrical Conductivity* Upon surpassing the percolation threshold, the electrical conductivity of graphene/polymer composites exhibited a distinct transition from insulating to conducting, showing a rapid surge in electrical conductivity near the percolation threshold followed by a more gradual increase (Fig. 14a). A comprehensive comparison of graphene/polymer composites featuring different graphene network configurations is illustrated in Fig. 14c. Composites reinforced with graphene aerogel, dispersed graphene and segregated graphene commonly employ GO as the graphene precursor, as the precursor for graphene, owing to its ease of processing and favorable compatibility with polymer matrices. The attainment of electrical conductivity is realized through the partial reduction of the GO precursor, resulting in the effective conducting component of rGO within the composites. According to Fig. 14c, it is evident that aligned graphene aerogels represent the most effective rGO fillers among these categories [5, 65, 171, 190], because the arrangement of interconnected rGO building blocks in the oriented aerogel framework serves as efficient conductive pathways. Noteworthy examples include unidirectional freeze-cast graphene aerogel/epoxy composites, where the oriented aerogel frameworks yielded a commendable conductivity of 13.5 S m^−1^ at a minimal filler content of 0.11 vol% [65]. Furthermore, through a combination of hydrothermal and unidirectional freeze-drying processes, aligned graphene aerogels with enhanced wall thicknesses were achieved, leading to composites exhibiting an electrical conductivity of 980 S/cm at a filler content of approximately 0.4 vol% [171]. In contrast, the randomly oriented graphene aerogel, despite possessing a 3D network similar to that of highly aligned graphene aerogel, exhibited a comparatively less effective conductive behavior due to its more convoluted network structure [178, 191, 192].

Dispersed graphene reinforced polymer composites are commonly prepared using solution processing. With good dispersion and sufficiently large content of graphene, conductive networks can be formed. However, due to the inherent challenge in achieving uniform dispersion of graphene and rGO within polymer matrices, composites containing dispersed graphene sheets often exhibit a certain degree of agglomeration of the filler. This, in turn, results in relatively diminished electrical conductivities, even at higher filler contents [180, 196–199]. In the process of fabricating composites via the hot compression of graphene-coated polymer particles, segregated graphene-rich regions spontaneously form, engendering electrically conductive pathways. The inherent segregation of graphene within the polymer matrix gives rise to continuous three-dimensional networks, fostering electrical conduction and leading to relatively higher electrical conductivity compared to composites with dispersed graphene configurations [187–189, 200]. Composites incorporating networks of graphene grown via CVD, including graphene foam (GF) [177], graphene woven fabric (GWF) [194], and multilayer graphene web (MGW) [195], have demonstrated the most exceptional electrical conductivities within polymer composites. These exceptional conductivities of CVD-grown graphene reinforced composites are attributed to the 3D continuous network and the flawless graphitic structure of the graphene framework.

In summary, from the above comprehensive analysis of the electrical conductivity of graphene/polymer composites featuring different building blocks and structural arrangements, we can conclude that the contribution of these materials to electrical conductivities of composites follows a general trend: CVD-grown graphene network > highly-aligned graphene aerogel > randomly oriented graphene aerogel ≈ segregated graphene > dispersed graphene. Considering that the intrinsic electrical conductivity of rGO building blocks in graphene aerogels is significantly lower than that of graphene building blocks in CVD-grown networks, the pronounced contribution of the aligned structural configuration to enhanced electrical conductivity is evident.

#### Fracture Toughness

**Fig. 15:**
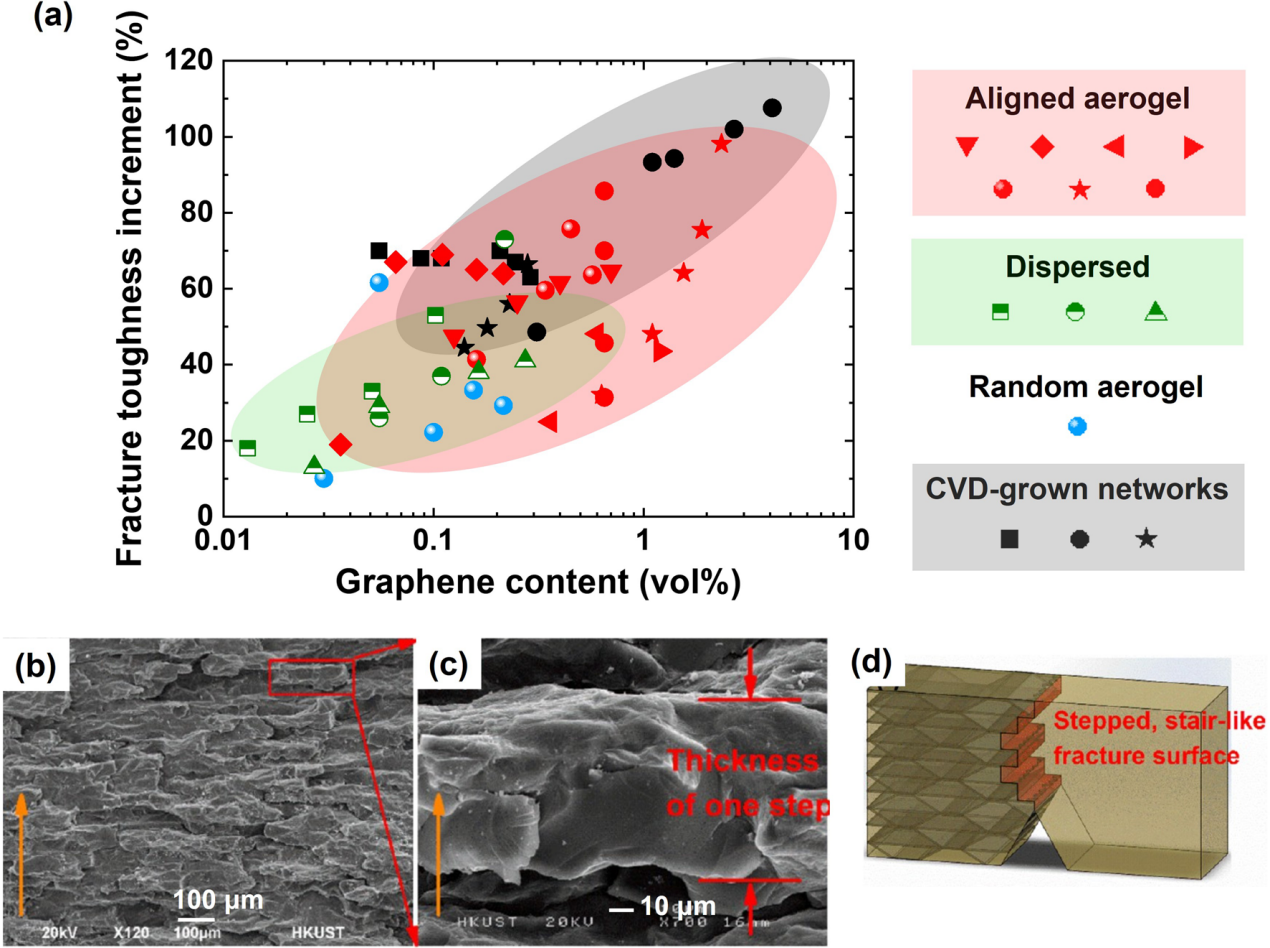
Fracture toughness of graphene/epoxy composites. **a** Increment in fracture toughness of polymer composites reinforced with different fillers: CVD-grown graphene [177, 194, 195], aligned graphene aerogel [11, 42, 65, 143, 166, 201, 202], random graphene aerogel [65] and dispersed graphene [203–205]. **b–d** Fractured cross-sectional SEM images and corresponding schematic crack prorogation process of aligned graphene aerogel/epoxy composite. Reproduced with permission [11]. Copyright 2015, American Chemical Society

Fracture toughness is a material property that quantifies a material's resistance to fracture or failure when subjected to a crack or other forms of stress concentration. It indicates the amount of energy a material can absorb before fracturing, which is a critical parameter for assessing a material's ability to withstand mechanical loads and resist crack propagation. Indeed, fracture resistance holds even greater significance and importance in practical engineering applications compared to modulus and strength. To gain insights into the contribution of highly-aligned graphene aerogels on composite toughness, a comparison of fracture toughness enhancement with the inclusion of various graphene additives is depicted in Fig. 15a.

The utilization of highly aligned graphene aerogels as reinforcement in epoxy composites results in significant enhancements in fracture toughness. An increment of 98% was witnessed in unidirectional freeze-cast graphene aerogel-reinforced epoxy composites, where the filler content was 2.4 vol% [201]. Epoxy composites incorporating highly aligned graphene aerogels, manufactured through the directional casting of large-size GO suspensions, experienced a notable enhancement of approximately 70% at a meager filler content of 0.11 vol% [65]. Another remarkable case is observed in bidirectional freeze-cast graphene aerogel-reinforced epoxy composites, featuring a bio-inspired nacre structure, which achieves a substantial increment of about 85% at a filler content of 0.65 vol% [202].

As comparisons, with equivalent rGO building blocks, it is evident that the aligned graphene aerogel [11, 42, 65, 143, 166, 201, 202] outperforms the randomly oriented graphene aerogel [65] and dispersed graphene [203–205] in terms of fracture toughness enhancement. CVD-grown graphene networks exhibit substantial impacts on the enhancement of the fracture toughness of epoxy-based composites. Notably, an exceptional enhancement of 50–70% can be achieved with a low graphene loading ranging from 0.005 to 0.28 vol% [177, 194]. At higher graphene contents, for instance, 4.1 vol% in the case of MGW, the enhancement reached approximately 108% [195]. With comparable enhancements in fracture toughness between composites reinforced by aligned graphene aerogel and CVD-grown graphene, we can see the significant role of the aligned structure in improving fracture toughness when taking into account the exceptional mechanical properties of nearly flawless and seamlessly continuous CVD-grown graphene.

The distinctive toughening mechanisms responsible for the substantial enhancement of fracture toughness in aligned graphene aerogel/epoxy composites are mainly attributed to the graphene framework induced crack reorientation. As shown in Fig. 15b–d, aligned graphene aerogel/epoxy composites exhibited fracture surfaces with a rough, stair-like appearance, as cracks were deflected by the aligned layers of GO [11]. This deflection resulted in the dissipation of a significant amount of energy, consequently leading to enhanced fracture toughness. The aligned graphene aerogels effectively blunted and diverted crack tips along the interfaces between rGO and epoxy, which effect was visually evident from the fracture surfaces, consisting of smooth epoxy regions measuring around 20–30 µm (subject to the aerogel fabrication process) in size, interspersed by the rGO networks.

#### Thermal Conductivity

**Fig. 16:**
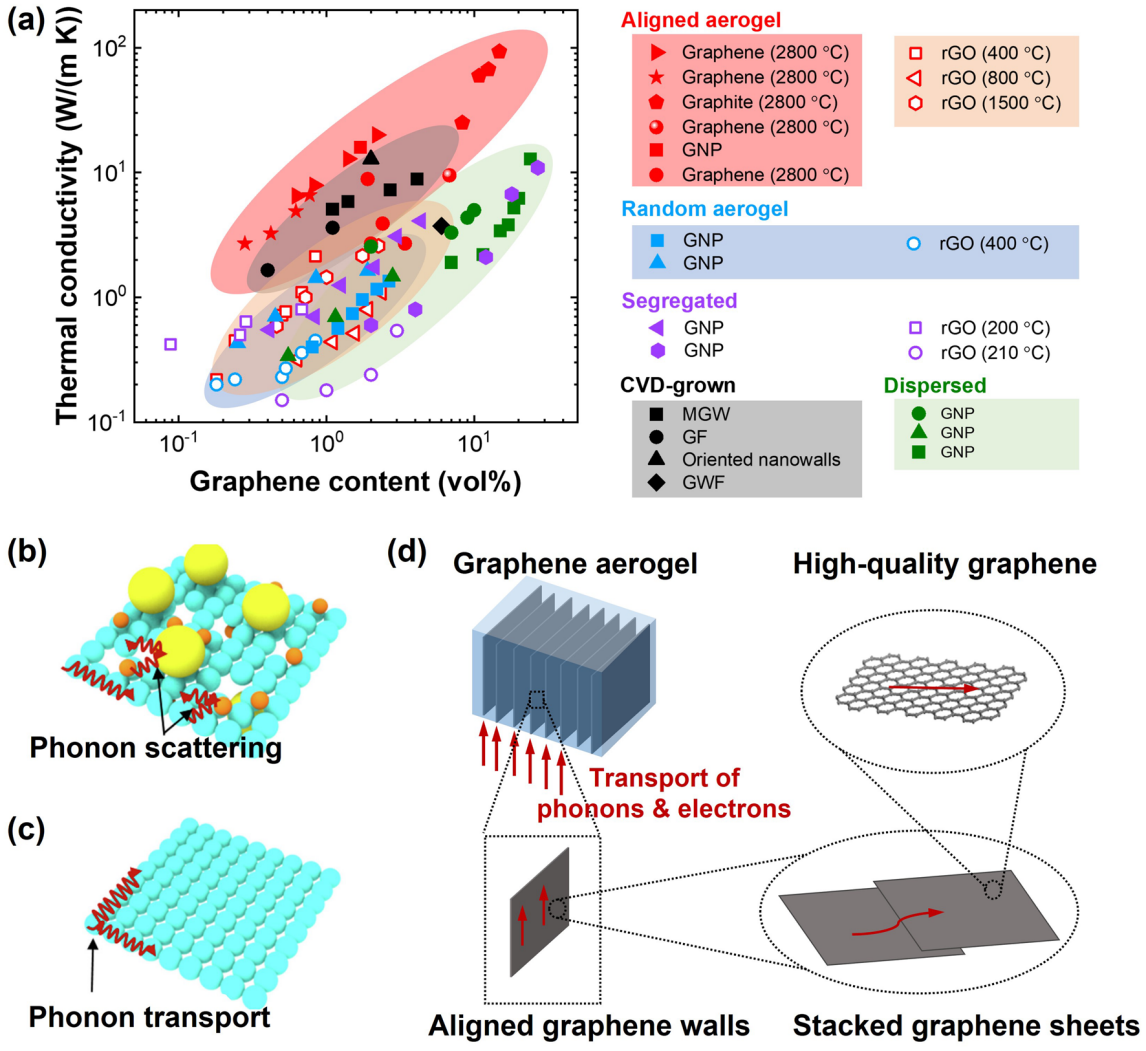
Thermal conductivity of graphene/polymer composites. **a** Thermal conductivity of graphene/polymer composites reinforced with different fillers: aligned graphene aerogel [64, 74, 102, 137, 166, 201, 207, 208, 221], random graphene aerogel [208–210], dispersed graphene [211–213], segregated graphene [214–217], and CVD-grown graphene [195, 218–220]. Schematic illustrations of thermal conduction in **b** defective rGO and **c** well-crystalline graphene sheets. Reproduced with permission [15]. Copyright 2021, Elsevier. **d** Schematic mechanisms of thermal conduction in highly aligned graphene aerogel/polymer composites. The solid and hollow symbols in **a** represent the graphene and rGO building blocks in the thermally conductive graphene network, respectively

Thermally conductive polymer composites enhance thermal management in industries ranging from consumer electronics to automotive and aerospace. They find crucial applications in efficient heat dissipation for high-power electronic devices and batteries, enabling improved device performance and reliability. Graphene, with its exceptionally high in-plane thermal conductivity of 5300 W m^−1^ K^−1^ [206], holds great promise for enhancing the typically low thermal conductivity of polymers of 0.1–0.3 W m^−1^ K^−1^. To maximize the contribution of graphene additives, a high quality of graphene additives is important. Highly-aligned graphene aerogels with well-crystalline structures induced by thermal annealing at 2800 °C (pink area in Fig. 19a) [64, 74, 137, 166, 207] significantly outperform partially reduced and aligned counterparts (orange area) [102, 201, 208]. Composites reinforced with aligned graphene aerogel reduced at 2800 °C achieved a remarkable thermal conductivity of approximately 6.6 W m^−1^ K^−1^ at a mere 0.77 vol% graphene content (solid stars) [64], while a much higher filler loading of around 2.3 vol% rGO was necessary for an even lower thermal conductivity of 2.6 W m^−1^ K^−1^ (red hollow hexagons) [102]. The superior thermal conductivity of high-quality graphene arises from reduced defects and minimized scattering of phonons or electrons, as shown in Fig. 16b, c [15].

The arrangement and alignment of graphene play a pivotal role in enhancing the thermal conductivity of graphene/polymer composites, as illustrated in Fig. 16a. Highly-aligned graphene aerogels, thermally reduced at 2800 °C [64, 74, 137, 166, 207], outperform random graphene aerogels [208–210], dispersed [211–213] and segregated rGO or graphene nanoplatelet (GNP) [214–217] in enhancing thermal conductivities of composites. They can even surpass the thermal conductivity of CVD-grown graphene/polymer composites [195, 218–220]. A polymer composite reinforced with 2800 °C-reduced bidirectionally freeze-cast graphene aerogel, possessing lamellar alignment and high-quality graphene building blocks, achieved an impressive thermal conductivity of approximately 20 W m^−1^ K^−1^ along the alignment direction at a graphene content of 2.2 vol% [166]. A high filler content of 14.8 vol% was achieved by 2800 °C-annealed shear-aligned graphite oxide array, contributing to an exceptionally high thermal conductivity of 93 W m^−1^ K^−1^ along the alignment [137]. In composites reinforced with CVD-grown graphene, the presence of vertically oriented graphene nanowalls [219] results in higher thermal conductivity compared to composites with random graphene networks [195, 218, 220], providing additional evidence for the beneficial impact of alignment.

The strategies devised to achieve elevated thermal conductivities in composites across various length scales are illustrated in Fig. 16d [195]. At the nanoscale, the 2800 °C annealed graphene building blocks showcase almost flawless crystallinity, leading to a scarcity of phonon scattering sites and yielding graphene sheets with remarkably high intrinsic thermal conductivities. On the microscale, the stacking and interconnection of graphene sheets within aerogel walls facilitate efficient phonon transfer across interfaces due to closely aligned phonon vibrational frequencies. On a larger microscopic scale, the organized alignment of graphene sheets constructs aerogel walls that substantially decrease phonon scattering at filler/polymer interfaces, thereby promoting extended-range phonon transport.

## Quantitative Characterization Techniques of the Graphene Alignment

### SEM Image-Based Orientation Distribution Analysis

SEM and transmission electron microscopic observations are a highly visualized techniques for alignment characterization, elucidating micro- and nano-scale morphologies across different orientations, respectively [222]. On the basis of SEM images, the extent of alignment finds quantitative assessment through orientation distribution analysis [223, 224]. This method conventionally involves the selection of a statistically substantial graphene sheet pool from SEM captures. Subsequently, reference lines are inscribed on these images, and the acute angles (*η*) between the reference line and individual graphene sheets are recorded, enabling rigorous quantitative analysis [5]. The orientation distribution function, *N*(*η*), is suitably modeled using the GaussAmp peak function:

4 $$N\left(\eta \right)={N}_{0}+A{e}^{-\frac{{(\eta -{\eta }_{c})}^{2}}{2{w}^{2}}}$$

where *N*_*0*_, *A* and *η*_*c*_ are the baseline height, peak amplitude, and the angle corresponding to the peak center, respectively. The quantification of the angular displacement from the peak center, denoted as *w*, is ascertained through the full width at half maximum (FWHM), as outlined below:

5 $$w=\frac{FWHM}{2\sqrt{ln4}}$$

The degree of graphene alignment is quantitatively assessed using the average orientation angle, ⟨cos^2^ *η*⟩, with respect to the preferred orientation direction. The ⟨cos^2^ *η*⟩ defines the proportion of graphene sheets within the angular element, d*η*, as indicated by [225]:

6 $$\langle {cos}^{2}\eta \rangle =\int_{0}^{\pi /2}N\left(\eta \right){cos}^{2}\eta sin\eta d\eta$$

where *N*(*η*) determines the portion of graphene falling within the angular interval d*η* and satisfies the normalization function:

7 $$\int_{0}^{\pi /2}N\left(\eta \right) sin\eta d\eta =1$$

**Fig. 17:**
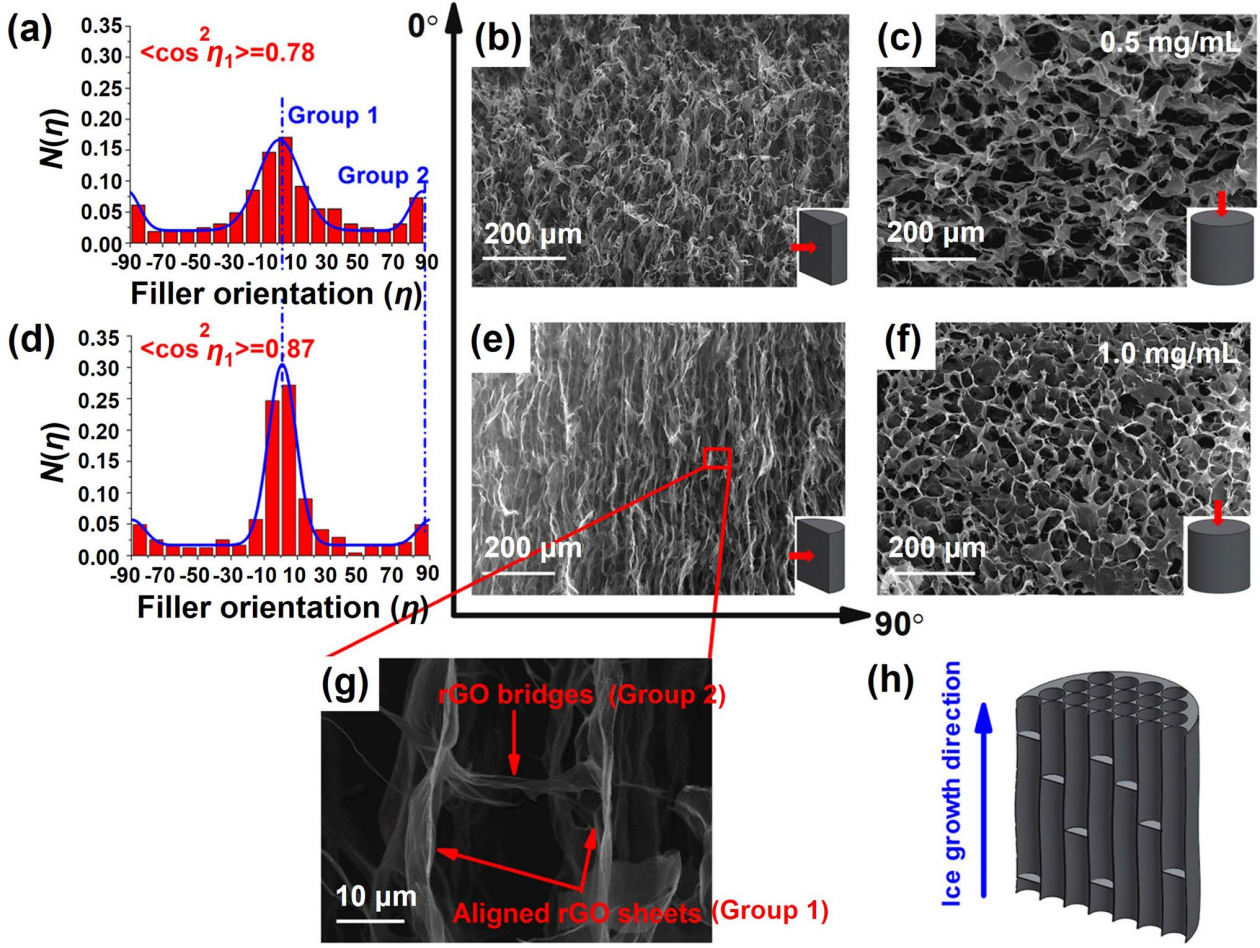
Quantitative analysis of the alignment in graphene aerogels using filler orientation distributions. Reproduced with permission [5]. Copyright 2016, American Chemical Society

Wang et al. quantitatively describe the alignment degree in unidirectionally cast graphene aerogels by the average orientation angle [5], a concept that also extends to the assessment of filler alignment in composites [175]. The parameter ⟨cos^2^ *η*⟩ serves as a metric for the alignment, which effectively characterizes the degree of orientation. The ⟨cos^2^ *η*⟩ values of 0 and 1 indicate elements that are respectively perpendicular to or aligned along the preferred orientation, while a ⟨cos^2^ *η*⟩ value of 1/3 signifies a 3D random distribution of graphene sheets. Application of the orientation distribution analysis to a typical unidirectional freezing graphene aerogel reveals a distribution of graphene sheets spanning from − 90° to 90°, as shown in Fig. 17a, d. This distribution exhibits a pronounced peak at around 0°, signifying the alignment of graphene sheets along the unidirectional casting direction (Group 1), alongside a less prominent peak centered at approximately ± 90°, corresponding to bridging elements amidst alignments (Group 2), as shown in Fig. 17g. Evaluating ⟨cos^2^ *η*_1_⟩, which pertains to the orientation angles of the predominant group (Group 1), elucidates that graphene sheets in aerogels fabricated using 1 mg mL^−1^ GO dispersions (Fig. 17b, c) achieved a higher degree of alignment compared to those prepared with 0.5 mg mL^−1^ GO (Fig. 17e, f).

### Polarized Raman Spectroscopy

**Fig. 18:**
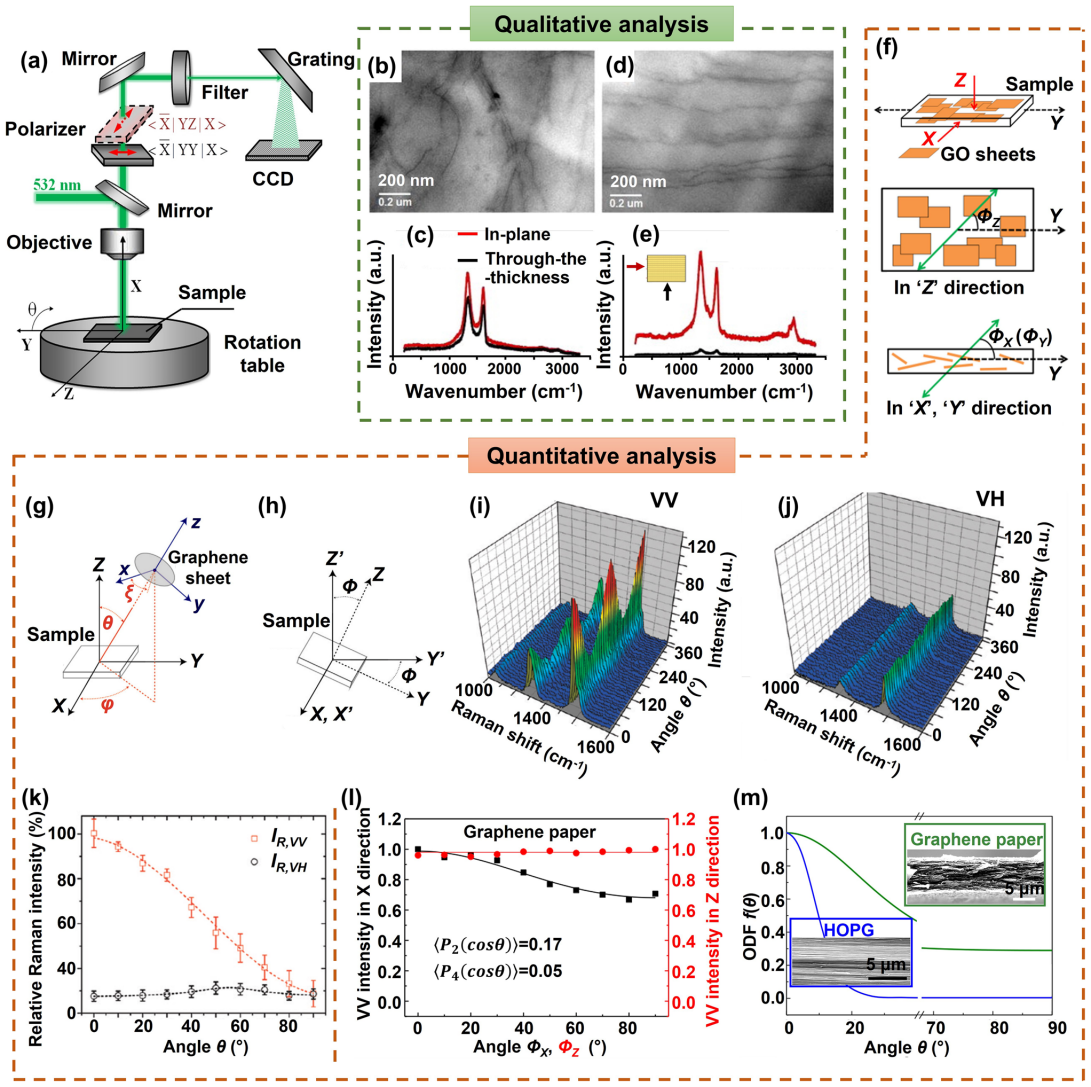
Qualitative and Quantitative analysis of graphene alignment by polarized Raman spectroscopy. **a** Schematic illustration of a typical polarized Raman spectroscopy. Reproduced under the terms of the Creative Commons CC BY license [226]. Copyright 2016, The Authors, published by Springer Nature. **b–e** Qualitative analysis of graphene alignment. Reproduced with permission [180]. Copyright 2013, Elsevier. Quantitative analysis of graphene alignment: **f** The Cartesian coordinate system with the sample geometries. Reproduced under the terms of the Creative Commons CC-BY license [230]. Copyright 2015, The Authors, published by Elsevier. **g** The local orientation of graphene in specimens, **h** coordinates of the specimen relative to the experimental measurement parameters. Reproduced with permission. Reproduced under the terms of the Creative Commons CC-BY license.[231]. Copyright 2015, The Authors, published by Elsevier. 3D Raman intensity for **i** VV and **j** VH polarization configurations, and **k** relative G band intensity. Reproduced with permission [31]. Copyright 2011, American Chemical Society. **l** Intensity variation for laser beam propagation in X and Z directions of graphene paper, and **m** orientation distribution function of HOPG and graphene paper. Reproduced under the terms of the Creative Commons CC-BY license [231]. Copyright 2015, The Authors, published by Elsevier. The designations VV and VH in **i–k** refer to the parallel and the perpendicular polarization of incident and scattered rays, respectively

Polarized Raman spectroscopy is a highly effective and widely embraced technique for characterizing the graphene alignment within diverse materials, which offers an efficient and non-destructive approach for assessing alignment quality, orientation, and the anisotropic attributes of graphene-based materials. A schematic representation of a typical polarized micro-Raman setup is presented in Fig. 18a. This apparatus enables precise control over the polarization of incident laser beams and facilitates the analysis of Raman-scattered light through the utilization of linear polarizers and wave plates [226]. The Raman scattering process within graphene is resonant, rendering it indifferent to the energy of the excitation laser. A wide spectrum of laser wavelengths, ranging from the infrared to the near ultraviolet, can be employed. Through an analysis of the intensity variations in the G- or 2D-band within the Raman spectrum, researchers can extract quantitative insights into the alignment characteristics of graphene within diverse materials. This analytical capability empowers researchers to fine-tune and regulate alignment processes, thereby achieving the desired properties tailored for specific applications.

The Raman spectra of graphene and its derivatives typically possess a D-band (~ 1350 cm^−1^) arising from vibrations at defects like vacancies, grain boundaries, and heteroatoms within graphene sheets, a G-band (~ 1580 cm^−1^) associating with in-plane lattice vibration, a shoulder peak D′ (~ 1620 cm^−1^) which signifies the presence of defects within the graphitic carbon–carbon structure, and second-order peaks of 2D (~ 2700 cm^−1^) and 2D′ (~ 3240 cm^−1^) [227, 228]. The absence of the D-band, which denotes the sp^3^ hybridization, is a common characteristic in chemically vapor deposition-grown or mechanically exfoliated graphene sheets due to the near-flawless nature of the graphitic structure with little defect [170].

The principles underlying Polarized Raman spectroscopy for assessing graphene alignment stem from the intricate interplay between incident light and the lattice structure of graphene, coupled with the resulting vibrational modes [222]. The intensity of Raman signals from graphene is notably influenced by the angle of polarization, demonstrating a robust Raman resonance along the alignment direction parallel to the polarization vector [229]. Polarized Raman spectroscopy enables the excitation of diverse polarization modes of incident light, encompassing those that are parallel (longitudinal) and perpendicular (transverse) to the alignment direction. The interplay between the light polarization and the oriented graphene lattice significantly influences the Raman scattering efficiency, leading to discernible changes in the Raman spectrum. By assessing the Raman signal under various polarization configurations, researchers can extract valuable information about the alignment direction and assess the extent of alignment. Notably, the G-band within the Raman spectrum of graphene serves as a pivotal marker, resonating with the in-plane vibrational motion of carbon atoms in the hexagonal lattice. As graphene sheets align, distinct alterations in the characteristics of the G-band peak can occur, thereby providing valuable information about the alignment state [180]. As an example, the alignment of graphene sheets in their polymer-based composites was qualitatively investigated by Kim and coworkers using polarized Raman spectroscopy [181, 222]. The Raman spectra acquired along different directions showed slight differences in composites characterized by randomly dispersed graphene, as shown in Fig. 18b, c. However, significantly higher G-band intensities were observed when the incident laser aligned with the preferred orientation compared to the configuration perpendicular to the alignment (Fig. 18d, e).

Polarized Raman spectroscopy is also an effective tool for the quantitative assessment of graphene's spatial orientation, as shown in Fig. 18f–m. In Fig. 18f, a Cartesian coordinate system is established, with the *X*, *Y*, and *Z* axes correspondingly defined with respect to the specimen [230]. The laser beam operates along the *Z*-axis (perpendicular to the specimen's top surface) as well as the *X* and *Y* axes (parallel to the surface). Employing fixed polarization configurations, spectra are commonly acquired by systematically rotating specimens to various angles, namely *Φ*_*X*_, *Φ*_*Y*_, and *Φ*_*Z*_, to align the laser beams parallel to the corresponding axis. The local direction of graphene sheets within a specimen is defined by denoting the surface normal vector as the *z* direction and the surface as *xy* plane, as shown in Fig. 18g [231]. The interrelation between the sample coordinates (*X*, *Y*, *Z*) and the graphene coordinates (*x*, *y*, *z*) is articulated through the Euler angles (*θ*, *φ*, *ξ*), where *θ* and *φ* represent the polar angles of the graphene surface normal vector in the sample Cartesian coordinates, and *ξ* designates the rotation angle of graphene. Illustrating the laboratory coordinates, Fig. 18h elucidates the manner in which specimens are systematically rotated by an angle *Φ* concerning the laser polarization directions [231].

The presented Fig. 18i, j showcases typical three-dimensional polarized Raman spectra of graphene composites, acquired across various *θ* values ranging from 0 to 360° in both VV and VH configurations [31]. The G-band intensity within the VV configuration displays cyclic fluctuations, exhibiting peaks and valleys every 90°, while this periodic trend is less discernible within the VH configuration. Consequently, to assess graphene's orientation, it becomes feasible to scrutinize intensities within a representative span of 0–90°, as shown in Fig. 18k. By using the G-band intensity at *θ* = 0° in the VV configuration as a baseline, the relative Raman intensity (*I*_*R*_) of the G-band within the VV configuration demonstrates a marked reduction as *θ* increases, signifying a prevalent alignment of graphene in composites along the direction of *θ* = 0° [31]. In contrast, the relative G-band intensity within the VH showed slight variations against *θ*, suggesting the absence of preferential orientation.

In cases where the orientation of graphene sheets uniformly changes across a shared plane, the orientation distribution function can be expressed as *f*_N_(*θ*) [230]:

8 $${f}_{N}\left(\theta \right)=\sum_{i=0}^{\infty }\frac{2i+1}{2}\langle {P}_{i}\left(cos\theta \right)\rangle {P}_{i}\left(cos\theta \right)$$

where P_i_(cos*θ*) represents the Legendre polynomial of the degree i and $\langle {P}_{i}({\text{cos}}\theta )\rangle$ denotes the mean value which can be expressed as follows:

9 $$\langle {P}_{i}\left(cos\theta \right)\rangle =\frac{\int_{\theta =0}^{\theta =\pi }{P}_{i}\left(cos\theta \right){f}_{N}\left(\theta \right)sin\theta d\theta }{\int_{\theta =0}^{\theta =\pi }{f}_{N}\left(\theta \right)sin\theta d\theta }$$

The Raman scattering intensity of the sample, *I*_sample_(*Φ*), can be determined by the polarization angle *Φ* in relation to the specimen. This is under the premise of a uniform distribution of the surface normal vectors around the *Z*-axis:

10 $${I}_{{\text{sample}}}\left(\Phi \right)={I}_{0}\left\{\frac{8}{15}+\langle {P}_{2}\left(cos\theta \right)\rangle \left(-\frac{16}{21}+{\frac{8}{7}{\text{cos}}}^{2}\Phi \right)+\langle {P}_{4}\left(cos\theta \right)\rangle \left(\frac{8}{35}-\frac{8}{7}{{\text{cos}}}^{2}\Phi {+{\text{cos}}}^{4}\Phi \right)\right\}$$

where *I*_*0*_ is the amplitude of incident light.For graphene, a non-polar material, the $\langle {P}_{i}({\text{cos}}\theta )\rangle$ is nonzero only for even I and the polarized Raman spectroscopy can determine $\langle {P}_{2}({\text{cos}}\theta )\rangle$ and $\langle {P}_{4}({\text{cos}}\theta )\rangle$. Despite the presence of oxygen-containing groups, the crystal structure of GO and rGO remains largely similar to that of graphene, resulting in analogous vibration modes and Raman spectra [232]. Therefore, the Eq. (9) can also be extended to quantitatively analyze the spatial orientation of both GO and rGO sheets. By fitting the experimental Ramana spectra to Eq. (10), $\langle {P}_{2}({\text{cos}}\theta )\rangle$ and $\langle {P}_{4}({\text{cos}}\theta )\rangle$ values can be obtained to quantificationally describe the spatial alignment of graphene in aerogels or composites. It is worth noting that the Eq. (10) is applicable to both the G band and the 2D band of the Raman spectra of materials, yielding consistent outcomes regardless of the polarizability tensors used in the computation [231]. The $\langle {P}_{2}({\text{cos}}\theta )\rangle$ serves as the primary parameter characterizing the average orientation angle of graphene, while $\langle {P}_{4}({\text{cos}}\theta )\rangle$ holds less significance [233, 234]. Generally, larger $\langle {P}_{2}({\text{cos}}\theta )\rangle$ values indicate better alignment of graphene flakes. For instance, $\langle {P}_{2}({\text{cos}}\theta )\rangle =1$ means a perfect alignment, while $\langle {P}_{2}({\text{cos}}\theta )\rangle =(3\langle {cos}^{2}\theta \rangle -1)/2=0$ indicates randomly oriented graphene sheets, except in an extremely unique scenario where all graphene sheets are aligned along the *Z* axis at an angle *θ* that gives $\langle {{\text{cos}}}^{2}\theta \rangle =1/3$. In this case, consideration of $\langle {P}_{4}({\text{cos}}\theta )\rangle$ becomes relevant. To illustrate, a $\langle {P}_{2}({\text{cos}}\theta )\rangle$ = 0.17 and $\langle {P}_{4}({\text{cos}}\theta )\rangle$ = 0.05 is reported for graphene paper (Fig. 18l) [231], indicating a subtle long-range nanoscale ordering in graphene paper from a quantitative standpoint.

Utilizing the values of $\langle {P}_{2}({\text{cos}}\theta )\rangle$ and $\langle {P}_{4}({\text{cos}}\theta )\rangle$ values obtained using experimental polarized Raman spectra, the orientation distribution function, *f*_*N*_, can be mathematically expressed as follows [235]:

11 $${f}_{N}\left(\theta \right)=Aexp\left[-\left({\lambda }_{2}{P}_{2}\left(cos\theta \right)+{\lambda }_{4}{P}_{4}\left(cos\theta \right)\right)\right]$$

where the coefficients A, λ_2_ and λ_4_ within the equation can be determined through numerical solutions [235]. A consistent *f*_*N*_(*θ*) suggests an absence of specific orientation in the graphene structure, while it rapidly diminishes in cases of significant alignment. Applying this principle, Li et al. studied the alignment of carbon flakes in investigated the alignment of carbon flakes in materials like graphene paper and highly-ordered pyrolytic graphite (HOPG), as shown in Fig. 18m [231]. The *f*_*N*_(*θ*) of HOPG shows a more pronounced decrease with increasing *θ* compared to that of graphene paper, indicating a more effective alignment of graphene sheets in HOPG. This alignment is further corroborated by SEM observations, as shown in the insets of Fig. 18m [231].

### X-ray Scattering

**Fig. 19:**
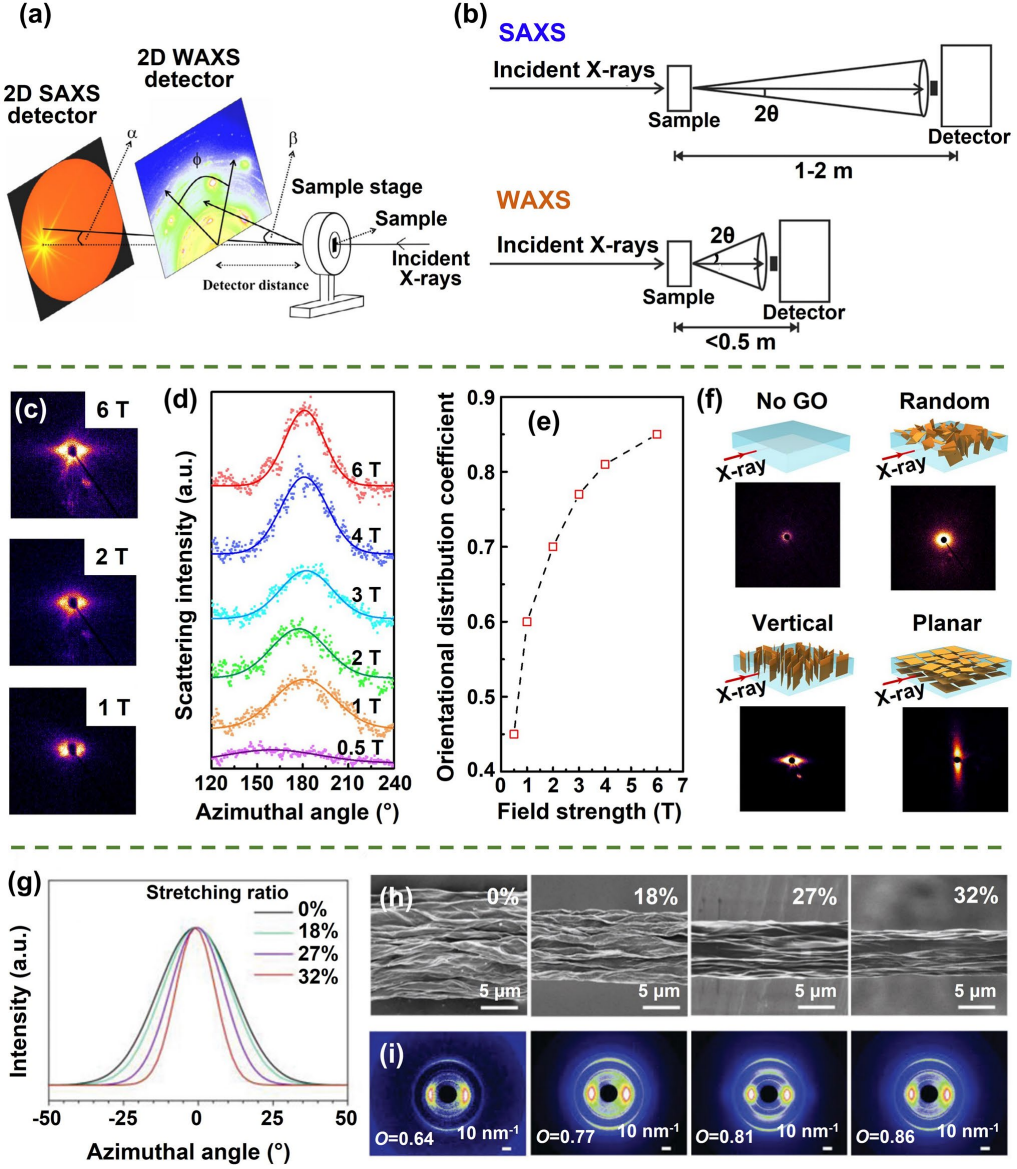
**a, b** Schematic illustrations of SAXS and WAXS techniques. Reproduced with permission [236, 237]. Copyright 2013, Elsevier. Copyright 2016, AIP publishing. Assessment of graphene alignment through SAXS: **c** 2D SAXS patterns, **d** the azimuthal dependence of scattering intensity, **e** orientational distribution coefficient of graphene-based materials fabricated under different magnetic field strengths; and **f** SAXS analysis of composites featuring diverse graphene alignment. Reproduced under the terms of the PNAS License [245]. Copyright 2017, National Academy of Sciences. Evaluation of graphene alignment using WAXS: **g** azimuthal scan profiles, **h** SEM images, and **i** 2D WAXS patterns of graphene fibers with different stretching ratios. Reproduced with permission [32]. Copyright 2020, Wiley–VCH

The X-ray scattering embodies the phenomenon arising from the interaction of X-ray photons with matter, giving rise to the scattering of these photons (Fig. 19a, b) [236, 237]. This process finds its explanation in two fundamental mechanisms: elastic scattering (Rayleigh scattering) and inelastic scattering (Compton scattering and X-ray fluorescence). Elastic scattering, also known as Rayleigh scattering, occurs when X-ray photons interact with the electrons of the atoms in the material without transferring any energy to them. This type of scattering preserves the energy and wavelength of the incident X-rays, but changes their direction. In the realm of elastic X-ray scattering, two distinct techniques stand out: small-angle X-ray scattering (SAXS) and wide-angle X-ray scattering (WAXS). The SAXS examines the scattering of X-rays at small angles (typically 0.1° to 10°) and offers insights into the nanoscale structure, including particle size, shape, and molecular arrangement. WAXS focuses on the scattering of X-rays at larger angles (generally larger than 10°) and provides detailed information about the atomic and molecular arrangement in crystalline materials [238]. The X-rays are scattered in all directions due to the presence of nano structures, resulting in a scattered X-ray pattern. This pattern, a consequence of the interference between scattered X-rays, engenders constructive and destructive interference, thus generating a diffraction pattern amenable to excavate valuable insights regarding the dimensions, geometry, and layout of nanoscale structures within the material [239].

In the case of graphene, SAXS is effective in deciphering the alignment of graphene sheets within the material. By analyzing the scattering patterns at small angles, SAXS can reveal the degree of ordering and orientation of graphene sheets, indicating the presence of aligned structures [240, 241]. Although graphene is not a traditional crystal, it possesses a highly ordered arrangement of carbon atoms within its two-dimensional lattice. On the other hand, WAXS can provide information about the crystal structure, lattice parameters, and interatomic distances in graphene. By examining the scattering patterns at wider angles, WAXS can reveal the orientation and alignment of graphene layers, providing insights into the crystallographic phases and orientation of graphene-based materials [32]. Overall, SAXS provides information about the overall nanoscale structure and alignment of graphene sheets, while WAXS offers insights into the atomic-level arrangement and orientation of graphene layers [241, 242]. By combining SAXS and WAXS techniques, researchers can gain a comprehensive understanding of the alignment of graphene. Considering the spot size of X-ray scattering, SAXS and WAXS provides average information of samples from macro to nano scale.

The quantitative analysis of graphene alignment is facilitated through the utilization of 2D SAXS and WAXS patterns. The extent of alignment of graphene sheets is precisely assessed by establishing a correlation between the azimuthal dependency of the scattering intensity, denoted as *I*(*θ*), and an orientational distribution coefficient, *O*. This correlation is achieved through the application of Hermans’ distribution function [243]:

12 $$O=\langle \frac{1}{2}\left(3{{\text{cos}}}^{2}\theta -1\right)\rangle$$

where *θ* represents azimuthal angle. The orientation distribution coefficient can be correlated with the *I*(*θ*) by:

13 $$\cos^{2} \theta = \frac{{\mathop \smallint \nolimits_{0}^{\pi /2} I\left( \theta \right)\cos^{2} \theta \sin \theta d\theta }}{{\mathop \smallint \nolimits_{0}^{\pi /2} I\left( \theta \right)\sin \theta d\theta }}$$

By combining Eqs. (12) and (13), the orientational distribution coefficient, *O*, can be quantified. The *O* value is ranging from 0 to 1, where *O* = 1 indicates a perfect alignment of graphene sheets while *O* = 0 corresponds to a completely random orientation [244].

Lu and coworkers effectively demonstrated the manipulation of graphene alignment through the influence of an external magnetic field, as substantiated by comprehensive SAXS characterizations [245]. At a relatively modest external magnetic field strength of 1 T, the 2D SAXS pattern showcased an elliptical diffusive configuration, while the eccentricity of this elliptical pattern progressively increased at higher field strengths, as shown in Fig. 19c. To quantify this alignment, the orientational distribution coefficient was deduced through a Gaussian approximation fitting of the scattering intensity against azimuthal angle (Fig. 19d). Remarkably, the obtained results in Fig. 19e deliver a consistent raising of the orientational distribution coefficient, evolving from 0.45 at 0.5 T to a heightened value of 0.85 at 6 T. This unequivocally underscores the magnetic field's significant contribution towards enhancing the degree of graphene alignment. To delve deeper, different alignment characteristics of graphene within composites were further investigated utilizing 2D SAXS, as shown in Fig. 19f [245]. The scattering intensity of the pristine polymer exhibited a negligible profile due to the absence of graphene constituents. Conversely, when randomly dispersed, graphene induced a distinct enhancement in scattering intensity, manifesting as a broad and isotropic halo pattern. The vertical- and planar-graphene show anisotropic equatorial and meridional scattering patterns [246], respectively. These outcomes, accompanied by comparable orientational distribution coefficients approximating 0.85, definitively confirm the specific orientations of the respective graphene entities.

The assessment of graphene alignment in materials can also be carried out through the WAXS techniques. Qian et al. reported an orientational distribution coefficient of *O* = 0.8 in graphene film based on 2D WAXS patterns [9]. Complementing this analytical approach, alignment visualization was further corroborated via SEM characterizations. WAXS studies have also unveiled the alignment tendencies within graphene fibers subjected to varying stretching ratios, as shown in Fig. 19g–i [32]. As the stretching ratio increased, a noticeable reduction in graphene fiber diameter was observed, coinciding with the gradual diminishment of radial wrinkles along the fiber axis (Fig. 19h). Through a comprehensive analysis of the azimuthal angle-dependent scattering intensities in WAXS patterns (Fig. 19g), orientational distribution coefficients, *O*, were accurately calculated. For fibers subjected to the preparation process without stretching, an *O* value of 0.64 was recorded (Fig. 19i). Impressively, this value notably escalated to *O* = 0.86 in graphene fibers characterized by a stretching ratio of 32%. This signifies the contribution of WAXS to the quantitative study of alignment of graphene.

### Comparison of the Characterization Techniques

The three quantitative characterization methods are compared in Table 2. SEM image-based orientation distribution analysis, reliant on SEM images, enables the visual assessment of graphene sheet orientation and alignment. This approach, operating at the microscale, provides local insights into alignment variations within the aerogel but may lack comprehensive coverage. In contrast, polarized Raman spectroscopy, operating at the same scale, offers orientation information through the analysis of anisotropic vibrational modes in graphene layers. However, it also tends to provide localized information. On a broader scale, X-ray scattering techniques, including WAXS and SAXS, emerge as versatile tools capable of quantitatively assessing alignment, crystallinity, and spacing of graphene sheets, spanning from macroscale to nanoscale. The X-ray scattering techniques, which are more comprehensive and quantitative, are highly adaptable, offering insights into structural properties across a wide range of length scales and are thus highly valuable for studying the alignment degree in graphene aerogels in academic research endeavors.

**Table 2:** Comparisons of different quantitative characterization techniques of the alignment degree

Characterization techniques	SEM-based	Polarized Raman spectroscopy	X-ray scattering
Mechanism	SEM image processing and analyzing	Inelastic scattering of photons	Elastic scattering of X-rays
Orientation distribution function	$\langle {cos}^{2}\eta \rangle ={\int }_{0}^{\pi /2}N\left(\eta \right){cos}^{2}\eta sin\eta d\eta$	$\langle {P}_{i}\left(cos\theta \right)\rangle =\frac{{\int }_{\theta =0}^{\theta =\pi }{P}_{i}\left(cos\theta \right){f}_{N}\left(\theta \right)sin\theta d\theta }{{\int }_{\theta =0}^{\theta =\pi }{f}_{N}\left(\theta \right)sin\theta d\theta }$	$O=\langle \frac{1}{2}\left(3{{\text{cos}}}^{2}\theta -1\right)\rangle$
Quantifying criterion	⟨cos^2^ *η*⟩ = 1/3: random	$\langle {P}_{2}({\text{cos}}\theta )\rangle =0$: random	*O* = 0: random
	⟨cos^2^ *η*⟩ = 0 or 1: perfectly aligned	$\langle {P}_{2}({\text{cos}}\theta )\rangle =1$: perfectly aligned	*O* = 1: perfectly aligned
Scale	Microscale	Micro- to nanoscale	Macro- to nanoscale
Information obtained	Alignment; inter-wall spacing	Alignment; structural anisotropy	Alignment; crystallinity; inter-layer spacing
Characteristics	Best for visualizing local variations	Local alignment	Comprehensive information

## Multifunctional Applications

### Energy Conersion and Storage

#### Solar-Thermal Energy Conversion

**Fig. 20:**
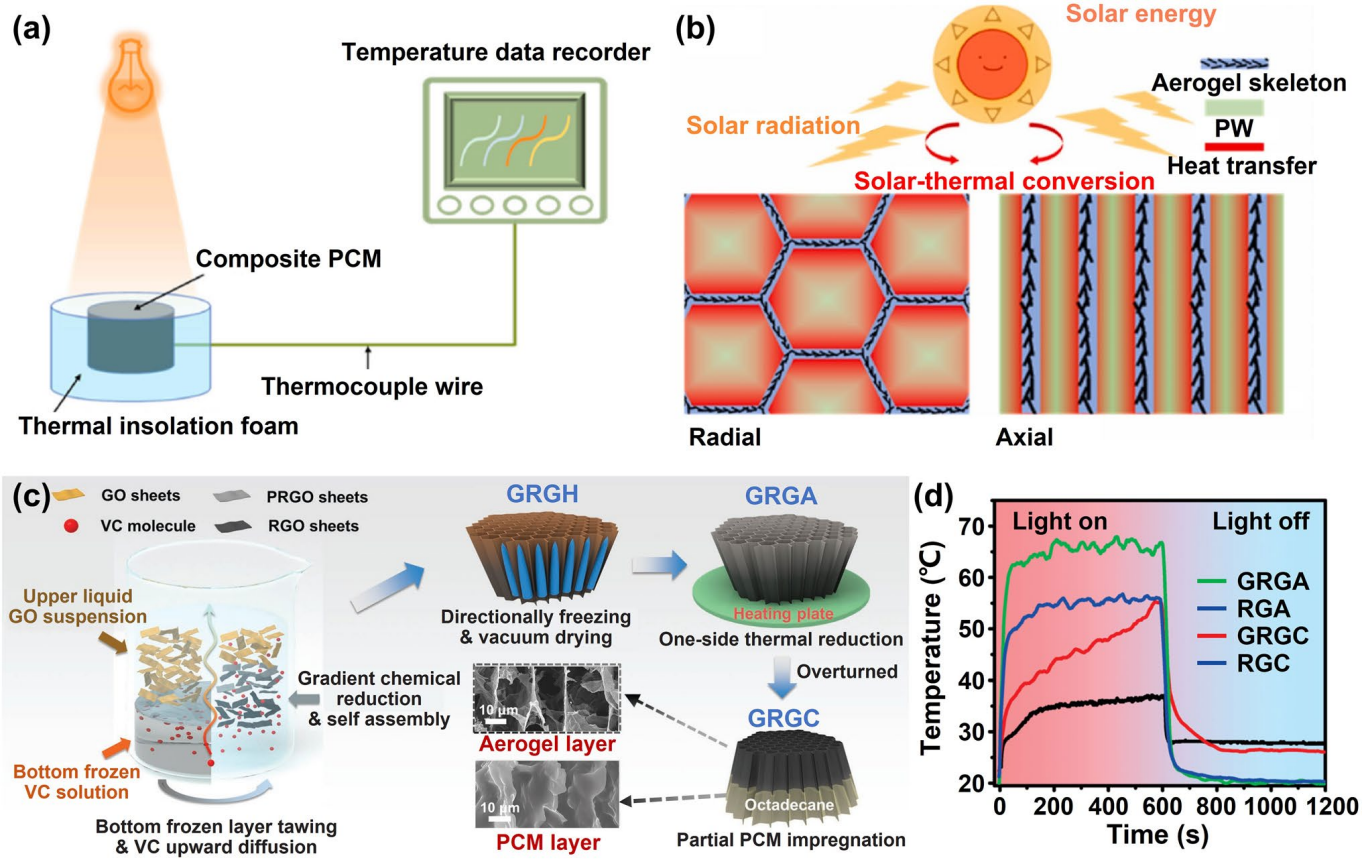
Solar-thermal energy conversion and solar stream of vertically aligned graphene-based aerogels. Schematic illustration of solar-thermal energy conversion **a** device and **b** mechanism. Reproduced with permission [255]. Copyright 2022, Elsevier. **c** Fabrication and **d** the surface temperature against sun radiation time of a gradient aligned graphene aerogel-based composites. Reproduced with permission [253]. Copyright 2023, Wiley–VCH

Solar-thermal energy conversion and storage harness renewable solar energy, addressing temporal, intensity, and spatial discrepancies between thermal energy demand and supply, and offering a potent means for solar energy utilization. Solar-thermal materials absorb energy from sunlight, raising temperatures during light exposure, and release energy gradually upon cessation, thus achieving effective energy storage and release (Fig. 20a) [247, 248]. To enhance solar-thermal energy storage, phase change materials (PCMs) are favored, but their intrinsic limitations such as low thermal conductivity and poor light absorption impede efficient energy conversion/storage [207]. Overcoming these challenges involves incorporating additives with a broadband absorption and thermal conductive nature. Effective strategies for enhancing the performance of solar-thermal materials include efficient sunlight absorption with low transmittance and reflectance, along with the fast transfer of heat induced by solar-thermal energy conversion from additives to thermal-energy-storage PCMs [249]. Large-specific-area aerogels, foams, and sponges with highly thermally conductive building blocks are promising additives for solar-thermal materials, with graphene aerogel standing out due to its broadband absorption, excellent stability, and thermally conductive nature [248, 250, 251]. The preconstructed aligned graphene aerogels show great potentials in achieving high thermal conductivity at relatively low filler loadings (Fig. 16a). The efficient solar absorption and conversion induced heat generation, the high thermal conductivity induced fast heat transfer to PCMs, and the low filler content of aligned graphene aerogels induced high latent heat retention synergistically result in enhanced solar-thermal energy conversion and storage properties of PCMs [7].

The black-colored graphene aerogels, obtained through intense reduction of GO aerogel precursors, exhibit impressive solar light absorption (over 95%), creating a foundation for efficient solar light utilization [252]. The captured solar light/photons are directly converted into heat and induce solar-to-thermal conversion effect, raising materials’ temperatures on surfaces and subsequently in interior structures through thermal conduction [253]. A rapid temperature raise on the top surface of graphene aerogel/PCM composites can be achieved from ~ 30 to > 90 °C upon 1-sun (1 kW m^−2^) radiation for ~ 3 s, inducing temperature gradients for heat transfer within composites [35]. The graphene nanosheets serve a dual role, effectively capturing solar energy to boost absorption and transforming it into thermal energy, while also functioning as heat-conductive additives that facilitate rapid heat transfer from the higher-temperature graphene framework to the lower-temperature PCM for efficient thermal storage, as shown in Fig. 20b [254].

The arrangement of graphene building blocks in PCM composites is highly correlated to the solar-thermal energy conversion. Though 3D disordered thermally conductive backbones can provide continuous heat transport pathway, aligned frameworks capitalize on the high aspect ratio and planar thermal conductivity of 2D materials more effectively, resulting in substantial enhancement of thermal conductivity and energy storage efficiency along the alignment direction (Fig. 20b) [221, 247]. Weaker phonon and electron scattering within the aligned framework in graphene aerogels endows more effective thermal conduction. Additionally, in aligned aerogels, heat transfer and photothermal conversion along the alignment are notably higher due to more effective thermal conduction [254], thus inducing faster temperature increases and higher final temperatures under aligned radiation [247]. An example is the aligned graphene aerogel/paraffin wax composite created via directional freeze-casting, reduction, and wax impregnation, exhibiting anisotropic thermal conductivity of 8.87 W m^−1^ K^−1^ along alignment and 2.68 W m^−1^ K^−1^ transversely, alongside 98.7% latent heat retention [74]. This composite achieves a final temperature of 80 °C, showcasing commendable energy conversion and storage capabilities. Additional to the direct conversion of solar and thermal energy, the aligned graphene-based aerogel/PCM composites can also be integrated into thermoelectric devices to improve the output voltage stability [221].

To further amplify the solar-thermal energy conversion potential of highly aligned graphene aerogel/PCM composites, a gradient structure approach is proposed. This strategy was realized through the creation of a gradient rGO aerogel-based bilayer PCM composite (GRGC) via a process containing gradient vitamin C-reduction of GO dispersion, unidirectional freeze-casting and drying, one-sided thermal reduction, and partial infiltration of an octadecane PCM, as shown in Fig. 20c [252]. This composite featured an upper aerogel layer with more extensively reduced rGO sheets and a lower aerogel/PCM layer with less pronounced reduction. Under 1-sun irradiation, the surface temperature of GRGC reached 55 °C, surpassing even that of fully loaded rGO aerogel composites (RGC), as shown in Fig. 20d. This novel structure not only enhanced heat absorption owing to its effective solar-thermal effect but also curbed heat dissipation to the surroundings due to the presence of the rGO aerogel layer.

#### Battery

Batteries with high-energy–density energy storage are critical for the development of modern electronic technologies, such portable electronics and electric cars. Graphene aerogels exhibit high electrical conductivity, hierarchically porous structure, and large specific surface areas, affording rapid electron/ion transport capacity during the reversible charge and discharge process [23]. The resilient 3D networks of graphene aerogel are both physically and chemically stable during the cyclic ion intercalation and deintercalation process, ensuring prolonged cycle lifetimes of batteries [23]. The highly ordered and interconnected framework, along with the oriented channels of aligned graphene aerogels facilitate the mass transport and ion diffusion along the alignment direction, resulting in improved reaction kinetics, faster charge/discharge rates, and enhanced cycling stability of electrochemical energy storage devices [255]. Aligned structures of graphene aerogel-based electrodes also effectively shorten the ion transport pathways and improve the rate performance of batteries, endowing rechargeable batteries with fast-charging capacities [256].

**Fig. 21:**
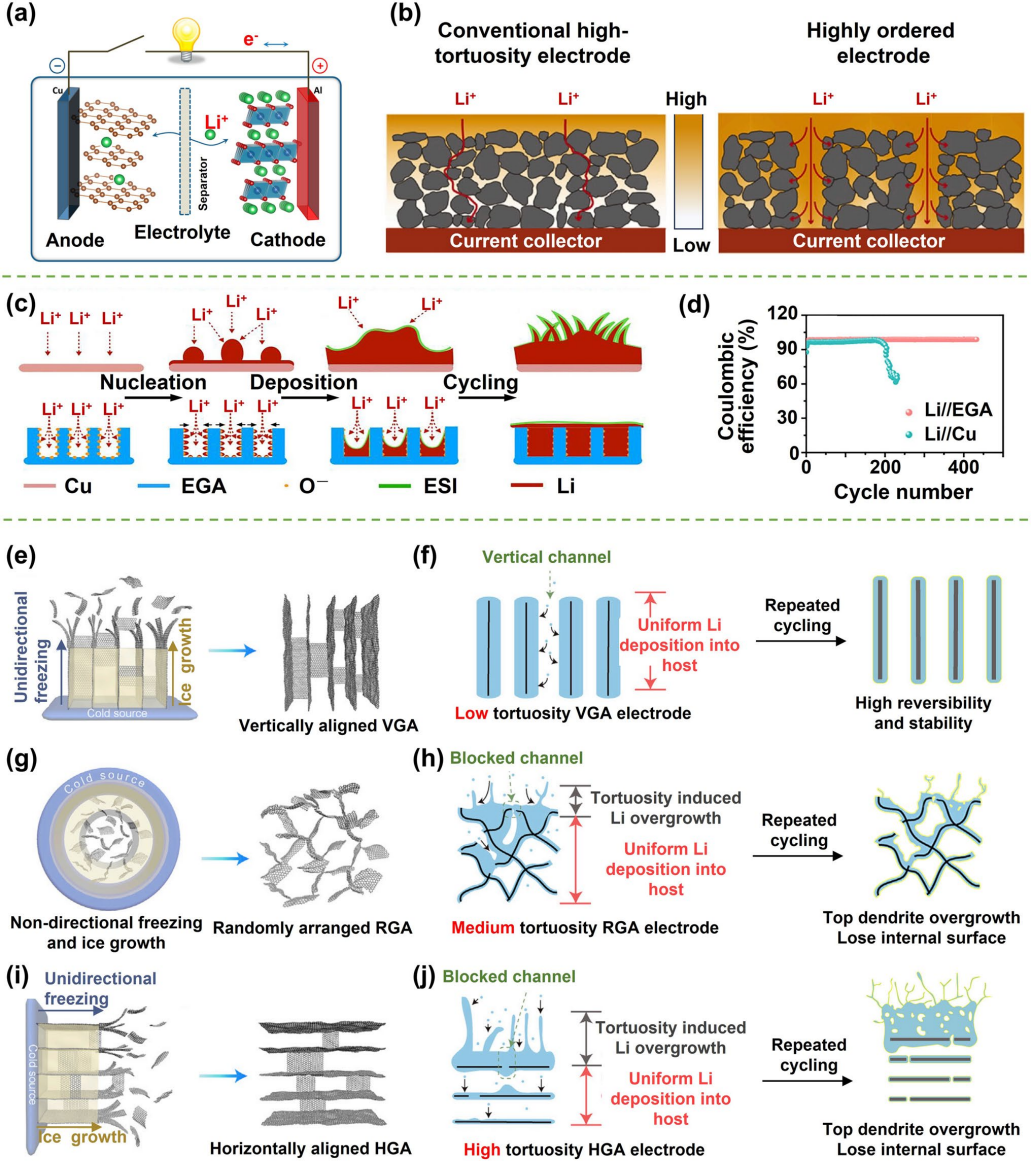
Highly aligned graphene aerogel-based composites for Li-ion batteries. **a** Schematic illustration of the Li-ion battery. Reproduced with permission [259]. Copyright 2013, American Chemical Society. **b** Li ion diffusion in different electrodes. Reproduced with permission [261]. Copyright 2020, Elsevier. **c, d** Thermally annealed aligned graphene aerogel for Li-batteries to hinder the growth of dendrite. Reproduced with permission [263]. Copyright 2021, Elsevier. **e–j** Comparison of the Li ion diffusion and dendrite growth in vertically aligned, horizontally aligned, and randomly arranged graphene aerogels. Reproduced with permission [265]. Copyright 2020, Elsevier

As an outstanding representative, Li-batteries are particularly promising for practical high-energy and high-power-density energy storage applications due to high theoretical capacity and good cyclic performances [257]. The Li ions move from the cathode, passing through the separator and electrolyte to reach the anode during the charging process of a Li-ion battery, and simultaneously produce electrons flowing from the cathode to the anode (Fig. 21a) [258]. According to the charging/discharging mechanism, the charging/discharging rate of a Li-ion battery is dependent on its electrodes, electrolyte, electrolyte/electrode interface and the transport of ions and electrons. Accelerating the transport of ions and electrons by minimizing the pathway is critical to increase the charging speed [259]. Aligned graphene aerogels, which are featured with oriented channels, provide straight ion diffusion paths for fast charging. Figure 21b shows simplified schematic illustrations showing enhanced ion transmission in the vertical pore channels [260]. It is easily seen that the diffusion of ions towards the electrode with highly ordered channels are easier and faster.

The development of Li-batteries faces a significant hurdle due to the uncontrollable growth of Li dendrites, originating from nonuniform plating and stripping of Li upon cycling and aggravating by unstable anode/electrolyte interphase properties electrode/electrolyte interphase properties (such as complex pathways for ion transport, large gradients in Li-ion concentration, significant chemical reactivity, and variable localized current densities) [261]. Taking advantages of highly porous, ultralow-volumetric-density and aligned architecture of oriented graphene aerogel, an electrode was developed by incorporating molten Li into 3D aligned graphene aerogel [50]. This approach effectively reduced local current density, restrained dendrite formation, mitigated volume variation, and promoted the utilization of Li, all of which contributed to the development of an efficient Li metal anode, achieving a lifetime of 5000 cyclic charging and discharging under a current density of 5 mA cm^−2^ in symmetric cells and a 1600-cyclic stability 2 C in full cells. The underlying mechanisms for the inhibition of dendrite growth are shown in Fig. 21c [262]. Traditionally, the nucleation of Li ions onto metallic current collectors shows inhomogeneity, leading to the aggregation of Li-ion nucleation and the subsequent non-uniform deposition of Li. As cycling progresses, this non-uniform nucleation exacerbates, resulting in the formation of dendritic structures with adverse effects, as shown by the schematic illustration of Li deposition on Cu foil in Fig. 21c. In contrast, the abundant and uniform lithophilic oxygen groups on graphene sheets obtained by controlled reduction of GO precursors provide conducive binding sites for uniform nucleation of Li ions (Fig. 21c). Additionally, the vertical microscale channels within the aligned graphene aerogel facilitate rapid transport of Li ions, fostering stable Li deposition and affording ample internal space for accommodating the expansion of the electrode at the cellular level [263]. The electrode surface retains a smooth, compact, and stable solid-electrolyte interphase (SEI), remaining free from dendritic growth over extended cycles. Consequently, stable cyclic performances (Fig. 21d), higher specific capacities with lower charge/discharge overpotentials are obtained.

The alignment contribution of graphene aerogel-based electrodes to Li batteries is further illustrated in Fig. 21e–j [264]. Distinct graphene aerogel—vertically aligned, horizontally aligned, and randomly arranged morphologies—result in different tortuosities for Li ion transmission of 1.25, 4.46, and 1.76, respectively. Larger tortuosities induce higher local current densities, leading to Li overgrowth on electrode surfaces and consequent degradation of cycling performance. The vertically aligned graphene aerogel electrode provide straight and inward channels for Li ion diffusion, allowing for highly reversible and uniform Li deposition within the internal electrode (Fig. 21e, f). Therefore, issues such as lithium overgrowth and dendrite formation are mitigated. In contrast, the tortuous randomly arranged and horizontally aligned graphene aerogel-based electrodes typically witness lithium deposition and stripping at their upper surfaces, hindering Li-ion diffusion into the internal regions and resulting in dendrite formation during cyclic performances (Fig. 21g–j). As a result, the low-tortuosity vertically aligned graphene aerogel-based electrode exhibits a Coulombic efficiency of approximately 99.1% during the high-capacity (5 mAh cm^−2^) and high-current–density (5 mA cm^−2^) charge/discharge cycles, much higher than that of the medium-tortuosity randomly arranged and the high-tortuosity horizontally aligned graphene aerogel electrodes.

#### Supercapacitor and Electrochemical Thermocell

**Fig. 22:**
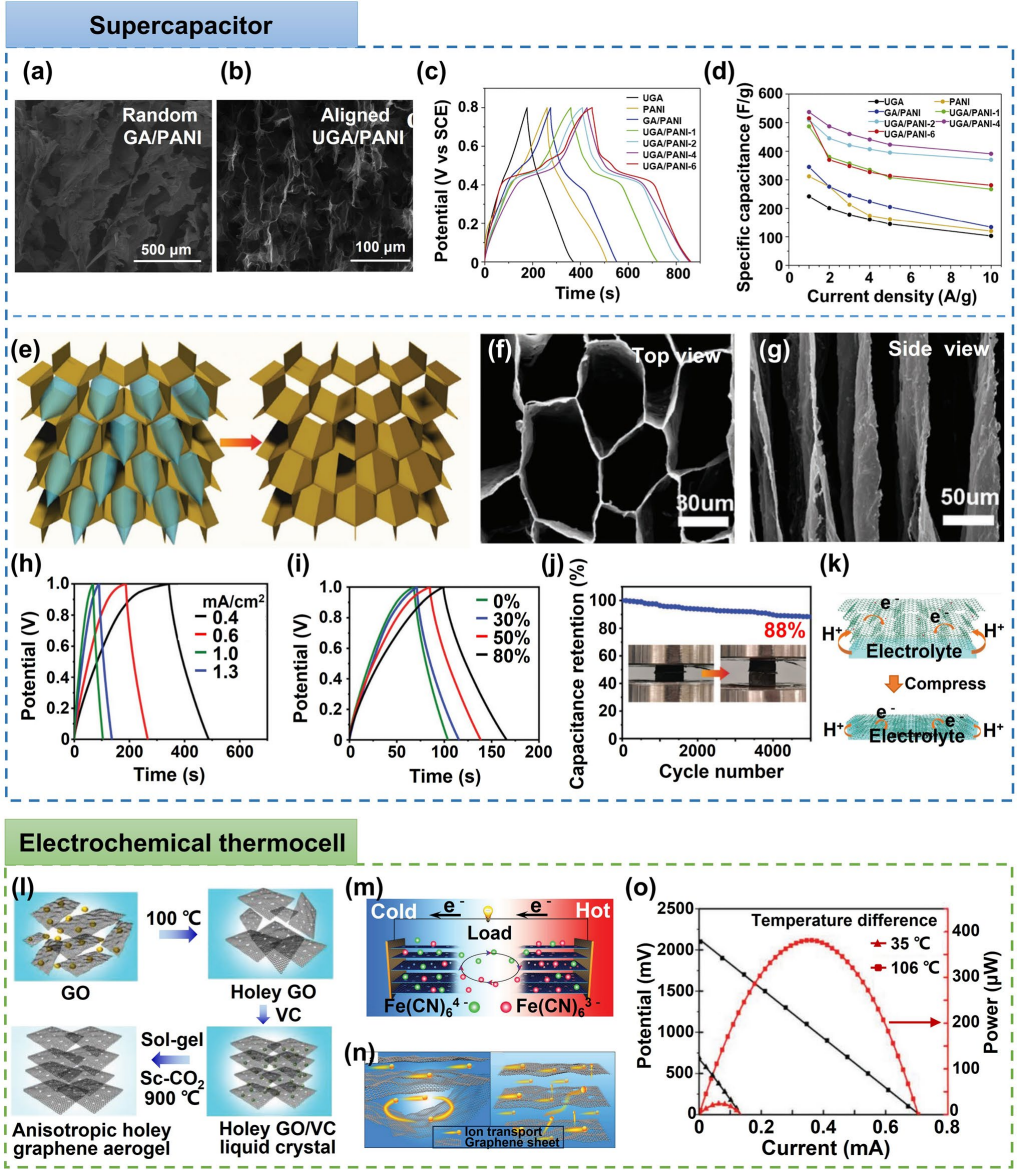
**a–d** Performances of supercapacitors with random and aligned graphene aerogel-based electrodes. Reproduced with permission [256]. Copyright 2018, Elsevier. **e–k** Directional freeze-casted graphene-based composite aerogels for compressible supercapacitors. Reproduced under the terms of the Creative Commons Attribution-NonCommercial License [272]. Copyright 2022, The Authors, published by Wiley–VCH. **l–o** Anisotropic holey graphene aerogel for electrochemical thermocell. Reproduced with permission [274]. Copyright 2019, Wiley–VCH

A supercapacitor, also known as an electrochemical capacitor or ultracapacitor, is an energy storage device that utilizes electrostatic double-layer capacitance between high-specific-surface-area electrodes and electrolyte, and/or pseudocapacitance generated during fast Faradic redox reactions to store and deliver electrical energy. Electrodes with large specific surface area, high electrical conductivity, remarkable porosity, lightweight and fast ion transport capability have been considered as ideal candidates for supercapacitors. Aligned graphene aerogels deliver high electrical conductivities along the alignment, significant structural stability under compression, and porous microstructure, making them highly promising applying as electrostatic double-layer or hybrid supercapacitors (extending even to flexible/compressible supercapacitors) [255, 265, 266]. As discussed above, the vertically oriented channels in aligned graphene aerogels provide straight and fast mass transport pathways, accelerating the ion diffusion process within the electrolyte (Fig. 21f, h, j) [266]. Therefore, contributions of aligned graphene aerogel to supercapacitors can be summarized as: (i) numerous depositive sites for active materials and fast penetration of electrolytes provided by the high specific surface area of aerogels; (ii) fast ion-transmission pathways provided by the straight vertically aligned channels; and (iii) fast electron transfer provided by the interconnected framework [255, 267–269]. With these synergistic effects of the alignment, the performance of aligned graphene-based aerogel electrodes surpasses that of random counterparts [270], mirroring the trend observed in the discussed battery electrodes. In a supercapacitor combining the electric double-layer capacitance of graphene aerogel and the pseudocapacitance of polyaniline (PANI) show broader galvanostatic charge/discharge curves for the aligned graphene/PANI aerogel electrode than that of nonaligned one (Fig. 22a–c) [255]. The specific capacitance of all aligned graphene/PANI aerogel electrodes are much higher than those of neat PANI and the random graphene/PANI aerogel electrode (Fig. 22d).

Aligned graphene aerogels exhibit excellent structural stability under cyclic compressions, making them promising for flexible and compressible energy storage in the field of emerging wearable electronics. Batteries, particularly those with organic electrolytes like Li batteries, pose risks due to toxicity and flammability, while the advent of flexible and compressible supercapacitors has addressed these concerns. A solid-state symmetric supercapacitor constructed using bidirectionally freeze-dried graphene-based aerogels with tubular morphologies (Fig. 22e–g). The nearly symmetrical triangle shapes of this supercapacitor show good capacitive properties, delivering a good area capacitance of 109.4 mF cm^−2^ at 0.4 mA cm^−2^ (Fig. 22h). Owing to the super-elasticity, super-compressibility, and excellent fatigue resistance of the aligned graphene-based aerogel, this electrode exhibits a compressive supercapacitance, maintaining 88% of its original performance after 5000 cyclic compressions to 50% (Fig. 22i–k) [271]. The galvanostatic charge/discharge curves of this compressive supercapacitor expand with increasing compressive strain (Fig. 22i), suggesting enhanced supercapacitance resulted from the intact aerogel framework and better interface contact between electrolyte and electrode under compression (Fig. 22k) [271].

Aligned graphene aerogels have also exhibited their potential in high-efficiency cryo-thermocells, which offer a viable way to continuously recycle waste and low-grade heat by the conversion of temperature-relevant electrochemical redox potentials into usable electricity [272]. An anisotropic graphene aerogel consisting of holey graphene building blocks was developed by self-assembly of holey GO sheets and subsequent thermal annealing (Fig. 22l) for the construction of thermocells [273]. With interpenetrating mesopores (induced by the holes in graphene sheets) and micropores (generated by oriented microchannels), the aligned graphene aerogel delivered enhanced ion transfer through reduced ion transport path and transmission impedance, as shown in Fig. 22m, n. The disparity in the redox potential of the electrolyte between the two aligned holey graphene aerogel electrodes was prompted by the temperature gradient experienced across the cell, driving the generation of electric power [274] (Fig. 22m). An open-circuit voltage output of 2.1 V and an output power of 0.37 mW at a temperature gradient of 106 °C were achieved by 15-thermocells in series, as shown in Fig. 22o.

### Super-Elasticity and Piezoresistive Sensors

The alignment in graphene aerogels and composite aerogels significantly enhances their structural stability, generating super-elasticity (the capacity to undergo large deformations and recover their original shape upon stress release), super-compressibility (the ability to endure significant compression while retaining their structural integrity), and excellent fatigue resistance (the capability to withstand and endure repetitive mechanical loading without experiencing significant degradation or failure) [266, 275].The excellent mechanical stability ensures the long-term durability and reliability of aligned graphene aerogels, making them suitable for applications where materials need to withstand cyclic loading without compromising their mechanical integrity [265].

**Fig. 23:**
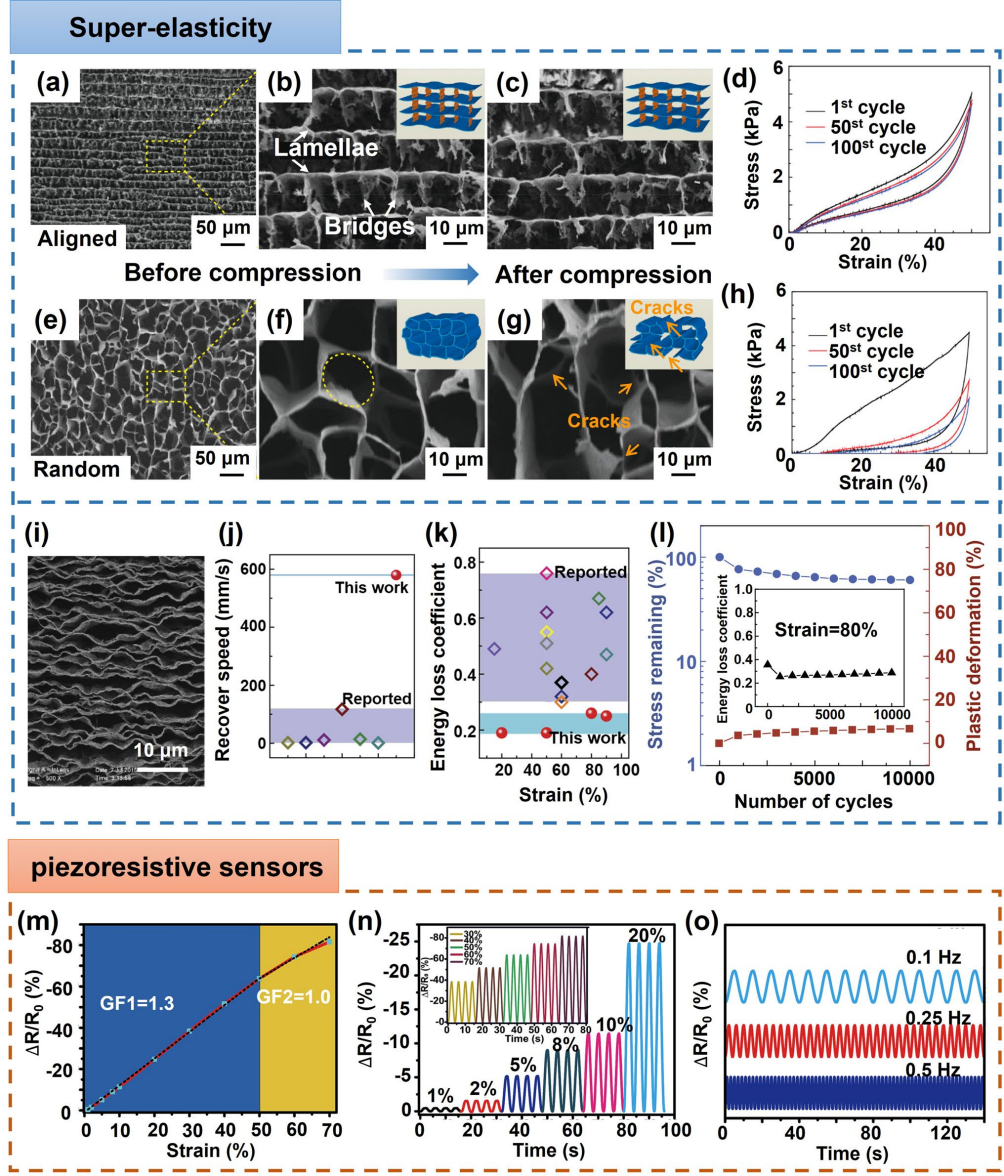
Super-elasticity and piezoresistive sensing of highly aligned graphene aerogels. **a–h** Comparison of the super-elasticity of aligned and random graphene aerogel. Reproduced under the terms of the Creative Commons CC BY-NC license [27]. Copyright 2017, American Chemical Society. **i** SEM morphology, **j, k** compressive elasticity and **l** fatigue resistance of an aligned arc-shaped lamellar graphene aerogel. Reproduced under the terms of the Creative Commons CC BY license [156]. Copyright 2016, The Authors, published by Springer Nature. **m–o** Super-elastic aligned graphene aerogels applied as piezoresistive sensors. Reproduced with permission [35]. Copyright 2021, Elsevier

The remarkable mechanical durability of aligned graphene-based aerogels has been elucidated through theoretical simulations and experimental assessments of both aligned and random architectures before and after compression. While these two distinct structures display similar maximum stresses during compression, the random structure exhibits comparatively less robustness and resilience [162]. In the architecture resembling plant stem-like “lamellar layers with interconnected bridges” (Fig. 23a–c) [27], stress is evenly distributed across the aligned walls, preventing stress concentration and material damage. The interconnected bridges effectively store compressive energy, leading to elastic deformation and impressive recovery. Acting as numerous “springs” between adjacent lamellar layers, these bridges foster dominant elastic deformation during compression cycles [276]. Post-compression, aerogels with highly oriented microscopic structures can almost fully revert to their original forms with minimal damage or deformation (Fig. 23c), which is also reflected by the comparable stress values during the cyclic loading (Fig. 23d) [161, 276]. Conversely, compressive loads on randomly arranged lamellae often create localized stress concentrations, resulting in skeleton cracks and monolith collapse and continuous reduction in compressive stress during the cyclic compression (Fig. 23e–h) [27, 160].

In addition to the plant stem-like structure, the incorporation of arc-shaped lamellae with preferential alignment along the lamella direction, achieved through bidirectional freeze-drying followed by structural shrinkage, also imparts exceptional mechanical stability (Fig. 23i–l) [156]. This arch-shaped cylindrical thin-shell model has the capability to endure substantial geometric deformations without undergoing yielding due to its minimal material strain, promptly restoring its original shape as well. The demonstration of graphene aerogels effectively rebounding a ball approximately 100 times heavier than the aerogels themselves showcases the remarkable structural stability of aligned graphene aerogels [94, 123, 156]. This outstanding structural stability of the highly aligned arc-lamellae structure (Fig. 23i) contributes to the rapid recovery rate of graphene aerogels upon the release of external loading, as shown in Fig. 23j.

The quantification of super-elasticity, super-compressibility, and fatigue resistance can be achieved through stress–strain curves [277]. The stress (or pressure) exhibits a linear-elastic increase during the initial compression stage, followed by a subsequent non-linear densification accompanied by a hysteresis loop (Fig. 23d) [160]. The pressure-strain loops partially overlap at different compressive strains, suggesting minimal structural damage during compression [72]. Energy dissipation reflects permanent failure of the aerogel framework, with greater dissipation (or narrower hysteresis loop) indicating more severe structural damage. When subjected to cyclic loading, highly aligned graphene-based aerogels display remarkable super-elasticity and fatigue resistance, maintaining minimal stress reduction at a maximum compression strain of 90% even after 1000 cycles of loading [276]. The compression and recovery curves coincide at zero point, indicating an excellent elasticity. With rationally designed microstructures, a near 100% height recovery after 50 cyclic compression to 90% can be achieved [123]. A 96.7% of original height is remained after 1000 compressive cycles to strains of 90% for graphene/konjac glucomannan aerogels fabricated by careful bidirectional freeze-casting [276]. The exceptional super-elasticity and robust fatigue resistance of aligned graphene aerogels are attained through the creation of a microstructure-driven hierarchical lamellar architecture containing numerous microscale sub-structures that function as elastic units, exhibiting a minimal energy loss coefficient, limited plastic deformation, and significant stress retention during cyclic compression (Fig. 23l) [156].

The exceptional properties of super-elasticity, super-compressibility, and outstanding fatigue resistance render aligned graphene and graphene-based composite aerogels suitable for piezoelectric sensor applications, where enduring mechanical stability during long-term repeated compression is crucial [76, 271, 278, 279]. These sensors find integration in various electronic systems, including artificial electronic skins, health monitoring, human activity recognition, human–machine interfaces, and wearable entertainment devices [280]. The mechanics underlying the piezo-sensitive electrical attributes of aligned graphene-based aerogels. Under compression, the electrically conductive graphene constituents within the highly porous aerogel scaffold are compacted, leading to larger-area contacts and a reduction in aerogel resistance [281, 282]. Piezoresistive sensor sensitivity hinges on the ratio of normalized resistance change (alteration in resistance relative to the original value) and strain/pressure [283]. Besides the compressive pressure-/strain- dependent resistance variation, variations in currents and capacitances can be employed for sensitivity evaluation [284, 285].

Both aligned neat graphene aerogels and graphene-based composite aerogels have been designed for piezoresistive sensing, where aerogels are electrically connected onto two electrodes with insulating polymer tapes covered outside for deformation detection. A bidirectionally freeze-dried lamellar graphene aerogels showed a high sensitivity of − 3.69 kPa^−1^, an impressive detection limit of 0.15 Pa capable of sensing dynamic force frequency and sound vibrations, and a robust stability over 10,000 cycles [87]. Highly aligned graphene aerogels achieved by calcium ion-assisted directional freeze-casting demonstrated a linear resistance alteration with compression strain, showcasing a sensitivity of ~ 1.3 in the 0–50% range (Fig. 23m) and remarkable reversibility under cyclic compression of various strains (Fig. 23n) and frequencies (Fig. 23o) because of their superb structural stability [35]. The introduction of polymers into aligned graphene aerogels further enhanced their structural stability and piezoresistive sensing properties [27, 265, 276, 286–288]. Silane-crosslinked and modified graphene aerogels exhibited an ultra-high sensitivity of − 67.1 kPa^−1^ in a low-pressure region of 0–5 kPa and a low detection limit below 30 Pa, serving as excellent pressure sensors [286]. By introducing electrically conductive nanofibers/nanotubes/MXene into aligned graphene aerogels serving as additional interconnectors and electrical conducting pathways, remarkable piezoresistive properties can be obtained [271, 279, 281, 288, 289]. For instance, an unidirectionally freeze-casted hybrid aerogel consisting of graphene, carbon nanotubes (CNTs), and cellulose nanofibers displayed exceptional properties including excellent compressibility (> 95%), remarkable elasticity (94.6% height retention over 50,000 cycles), outstanding sensitivity over compressive strain (15 within 5% strain), and a significantly low pressure detection threshold (0.875 Pa) [279]. Based on the reduced resistance under compression, aligned graphene-based aerogels can also be applied as switches, connecting circuit upon certain degree of compaction and cutoff when releasing [27, 282].

### Solar Steam

The efficient utilization of abundant solar energy for purifying contaminated water and desalinating seawater through solar steam generation stands as a sustainable approach to addressing the pressing global water scarcity challenge. The rate of water evaporation is closely tied to factors like the absorption of solar light, the transformation of solar energy into higher-grade thermal and steam energy via photothermal conversion, the water uptake, and the transportation of steam through solar-thermal conversion materials [290]. Building upon these principles, graphene aerogel-based materials hold great promise as solar steam generators due to their remarkable attributes including high specific surface area, exceptional solar light absorptivity, controllable porous frameworks, and remarkable structural integrity [207, 291]. Particularly, the aligned frameworks of graphene aerogels, characterized by meticulously engineered open channels for efficient water transportation and vapor release, surpass randomly oriented aerogels in the realms of water purification and desalination [30, 292].

**Fig. 24:**
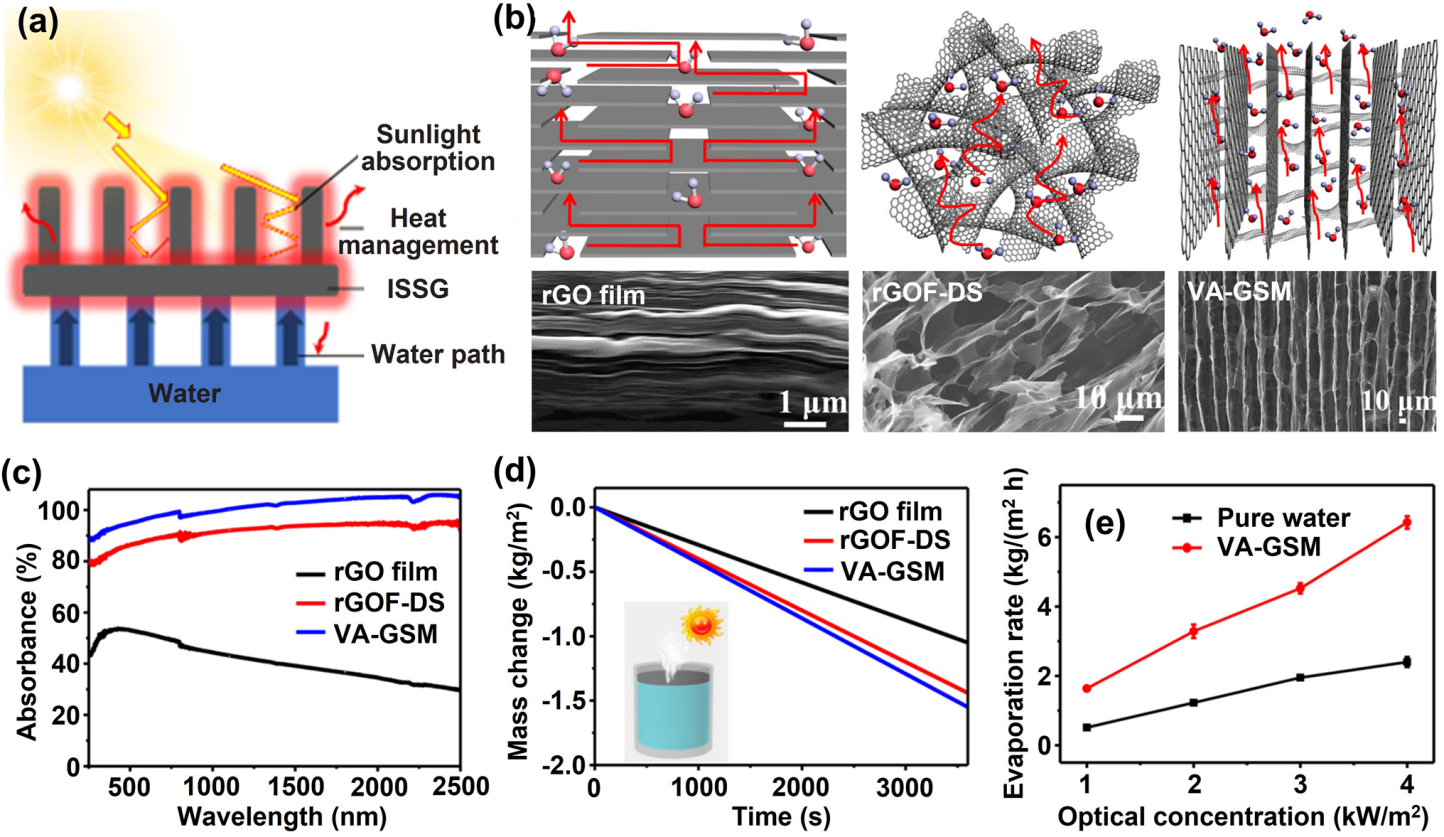
**a** Design idea of graphene based interfacial solar-steam generation system to enhance water evaporation. Reproduced under the terms of the Creative Commons Attribution License [294]. Copyright 2022, The authors, published by Wiley–VCH. **b–e** Solar steam of graphene paper, random and aligned graphene aerogel. Reproduced with permission [30]. Copyright 2017, American Chemical Society

The construction of an interfacial solar-steam generation system aims to concentrate light-induced heat onto steam generating materials while minimizing heat loss to the surrounding water, as shown in Fig. 24a [293]. The evolution of mass over time in the graphene aerogel-based solar stream system typically exhibits a linear pattern, indicating a stable and continuous solar steaming property [121]. Channels aligned vertically within the graphene aerogel can reflect solar incidents multiply to enhance the absorption of solar light and transfer to heats for steam generation, as well as facilitate swift upward water transport to the evaporation surface [294].

In comparison to rGO film featuring bulky aligned rGO sheets and randomly oriented rGO aerogels with a certain disordered structure (rGOF-DS), the highly-aligned rGO aerogels (VA-GSM) with vertically tubular channels exhibited enhanced performance in terms of solar light absorption, steam generation, and water transport, as shown in Fig. 24b–e [30]. The rGO film was characterized by a closely layer-by-layer assembled structure that significantly hampered the diffusion of water molecules in through-the-thickness direction (Fig. 24b). The closed-pore configuration of randomly oriented rGO aerogels (rGOF-DS) increased the transport distance of steam and thereby limited the evaporation. In contrast, vertically aligned rGO aerogel (VA-GSM), distinguished by its highly vertically aligned tubular structure, provided efficient open channels for water movement and steam release [295]. Additionally, the VA-GSM exhibited high solar light absorptivity of 93%, 98% and nearly 100% for the UV, visible, and near-infrared sunlight, respectively, far surpassing the performance of both the rGO film and rGOF-DS, as shown in Fig. 24c. Synergistic effects of the open channel, efficient sunlight absorption and fast water transport contributed to outstanding solar-thermal conversion efficiency of 83.5% under 1-sun radiation and continuous water supply for the generation of steam. Consequently, VA-GSM achieved an average water evaporation rate of up to 1.57 kg m^−2^ h^−1^ under 1 sun (Fig. 24d), and a higher rate of 6.25 kg m^−2^ h^−1^ under 4 suns (Fig. 24e).

The solar steam properties of aligned graphene aerogels can be further enhanced with the incorporation of solar-absorbable material, such as MXene [296], and CNT [297]. The MXene itself is also a promising solar-steam candidate due to its near 100% light-to-heat conversion efficiency [298, 299]. Typical MXene aerogels with different morphologies, such as fibrous [300], vertically aligned Janus [301], sandwich-structured [302], cellular [303] and feather-like aerogels [304], exhibit evaporation rates in the range of 1.2–1.9 kg m^−2^ h^−1^ under 1-sun irradiation. A hybrid structure comprising slightly reduced rGO (incorporating plentiful polar oxygen-containing groups to expedite water evaporation) and MXene (offering near 100% absorption of sunlight and efficient solar-thermal conversion) achieved a water evaporation rate of 2.09 kg m^−2^ h^−1^ under 1-sun exposure and expedited the desalination of seawater, boasting ion rejection rates exceeding 99% for most ions [305]. The simultaneous presence of various water states—bound water (water molecules bonded to polar groups in rGO sheets), free water (in regions distant from hydrophilic surfaces), and intermediate water in between—reduced the vaporization enthalpy and promoted solar steam generating [305]. The rGO/MXene hybrid aerogels with a configuration of "lamellar layers with interconnected bridges" further amplified multiple reflections of lights, consequently enhancing the effective absorption of solar incidence, obtaining an even higher water evaporation rate of 2.84 kg m^−2^ h^−1^ and a 96% of energy transfer under 1-sun irradiation [306], higher than MXene aerogels [300–304]. Similarly, the incorporation of CNTs into graphene aerogels has demonstrated a notable improvement in solar steam generation properties [307], showing great potential to achieve higher evaporation ratio compared to aerogels containing individual CNTs [308–311].

### Thermal Management

Thermally conductive graphene aerogels and their composites hold substantial promise in thermal energy conversion/storage and thermal transport applications. While their role in photothermal conversion and storage has been discussed in the preceding “solar-thermal energy conversion and solar steam” section, this segment focuses on their applications in heat transport. As previously introduced in the “Anisotropic properties” section, the thermal conductivity of highly aligned graphene aerogels and their composites exhibits anisotropy, rendering them ideal candidates for diverse thermal roles, including thermal conductors (owing to high thermal conductivity along the alignment), thermal insulators (due to low thermal conductivity transverse the alignment) or thermal regulators (with the assistance of phase change materials) based on their distinct thermal conduction attributes along different axes [118].

**Fig. 25:**
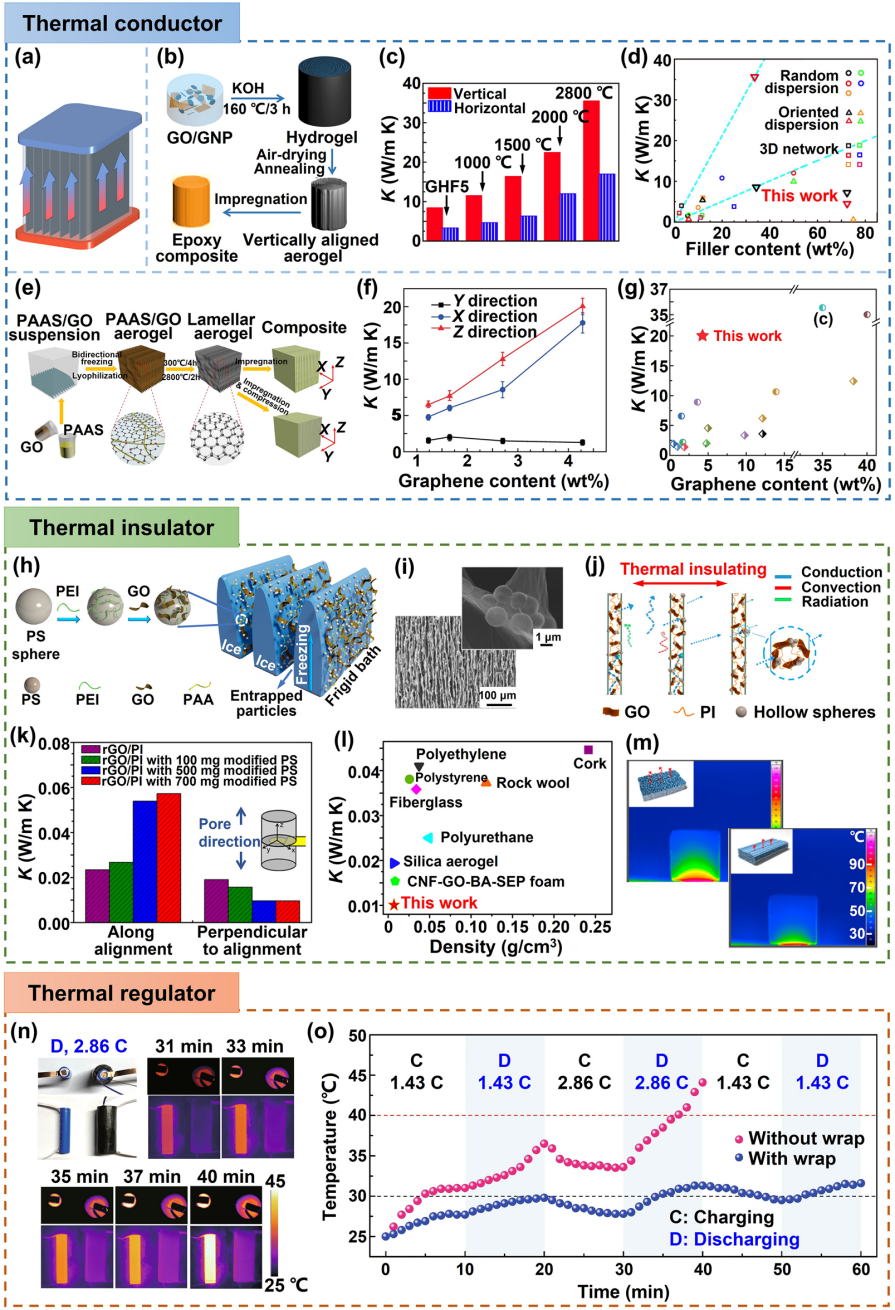
Highly aligned graphene aerogel-based composites applied in thermal management: **a–g** thermal conductors, **h–m** thermal insulators, and **n, o** thermal adjustors. **a** Vertically aligned graphene aerogel networks for TIMs applications. **b–d** Thermally conductive air-dried aligned graphene aerogel/epoxy composites. Reproduced with permission [165]. Copyright 2018, American Chemical Society. **e–g** Lamellar-structured graphene aerogel/epoxy composites for thermal conductors. Reproduced under the terms of the Creative Commons CC BY license [166]. Copyright 2020, The Authors, published by Springer Nature. **h–m** Highly oriented hierarchical graphene-based aerogels as thermal insulators. Reproduced with permission [37]. Copyright 2017, American Chemical Society. **n, o** Graphene aerogel/PCM composites for thermal adjusting. Reproduced under the terms of the Creative Commons CC BY license [102]. Copyright 2021, The Authors, published by Springer Nature

The establishment of pre-constructed 3D continuous networks, which effectively diminish thermal interfacial resistances between graphene and polymers, the preferred alignment enabling directional heat dissipation, and the exceptional thermal conductivity of high-quality graphene, synergistically enable efficient heat management and thermal dissipation in composites based on aligned graphene aerogels [15, 312, 313]. This makes vertically aligned graphene aerogel-based composites particularly suitable for thermal interface materials (TIMs) (Fig. 25a), where substantial thermal conductivities along the vertical direction are crucial for effectively dissipating heat from electronic devices [31, 158, 164, 312]. The air-dried, self-assembled graphene aerogel demonstrated a distinct vertical orientation of graphene building block and a substantial graphene loading of 35 wt% within epoxy composites, as shown in Fig. 25b [165]. Upon subjecting the graphene aerogel to thermal annealing, aimed at reinstating the highly thermally conductive graphitic structure within the building blocks, the thermal conductivities of aligned graphene aerogel/epoxy composites witnessed significant improvement. For composites incorporating 35 wt% (or 19 vol%) of graphene aerogel annealed at 2800 °C, thermal conductivities surged to 35.5 W m^−1^ K^−1^ (Fig. 25c). This remarkable enhancement reflected an efficiency increase of 884%, exhibiting its superiority over a variety of materials (Fig. 25d). Thermally conductive lamellar-structured graphene aerogel/epoxy composites were fabricated by bidirectional freeze-casting, followed by a high-temperature annealing process at 2800 °C and subsequent epoxy impregnation, with the graphene content further improved by compressing the lamellar aerogel after epoxy infiltration (Fig. 25e) [166]. This composite exhibited distinct thermal conductivities across different directions, with the highest conductivity of 20.0 W m^−1^ K^−1^ achieved along the vertical alignment at a graphene content of 4.28 wt% (or 2.3 vol%) (Fig. 25f), which is a striking 100 times greater than that of neat epoxy. The exceptionally high thermal conductivity at a relatively low filler loading underscores the substantial contribution of aligned graphene networks to the composite's thermal conductivity (Fig. 25g).

The anisotropic microstructures within oriented graphene and composite aerogels substantially improve the thermal conducting and dissipating efficiency along the alignment direction. This intricate arrangement effectively mitigates heat localization while reducing the heat transfer across the aligned porous channels in the transverse direction, giving composites low thermal conductivities perpendicular to the alignment [37, 78]. Building on this anisotropy, a further reduction in thermal conductivity of aligned graphene aerogels perpendicular to the alignment can be achieved by introducing scattering interfaces (such as incorporating a second component) [34, 314], and embedding hierarchical microstructures (like hollow spheres on the aerogel framework) [315]. For instance, the directionally freeze-dried rGO/polyimide (PI) composite aerogels delivered a remarkably low thermal conductivity of 0.012 W m^−1^ K^−1^ perpendicular to the alignment, rendering them highly promising for applications in thermal insulation, particularly in aerospace, energy-efficient buildings and wearable devices [163]. Peng et al. fabricated a hierarchical rGO/PI composite aerogel through a multi-step process including directional freeze-casting of a suspension which contained polystyrene (PS) microspheres, poly(ethylenimine) (PEI), GO sheets, and PI precursors, followed by a low-temperature thermal annealing which removed the sacrificial PS and PEI, reduced GO, and facilitated the formation of PI, as shown in Fig. 25h [37]. The elimination of PS microspheres resulted in the creation of hollow microspheres within the aligned rGO/PI frameworks (Fig. 25i), which considerably obstructed thermal transport by enhancing interfacial phonon scattering, as highlighted in the enlarged area of Fig. 25j. The integration of these hollow microspheres onto the aerogel walls significantly reduced the thermal conductivity of the rGO/PI composite aerogel when measured perpendicular to the alignment, decreasing it from 0.019 to as low as 0.009 W m^−1^ K^−1^ (Fig. 25k). This composite presented a notable reduction compared to conventional polymer-based thermal insulators and natural insulating materials (Fig. 25l). As a demonstration, the anisotropic thermally insulating properties of highly oriented composite aerogels can be visualized by thermographic images (Fig. 25m) [37].

Thermal insulation plays an important role in enhancing fire retardancy by impeding flame spread through the reduction of heat conduction, a pivotal mechanism for decelerating fire progression [316]. The prevention of localized overheat by thermal insulating materials significantly reduces ignition risks. The inherent advantages of graphene, with its strong *sp*^*2*^ carbon structure, make it particularly well-suited for fire retardancy by preventing the permeation of oxygen into materials and acting as an anticorrosion and flame-retardant layer [317]. Graphene aerogels, showing the synergistic effects of their 3D porous structure's thermal insulating property and graphene's flame-retardant nature, have demonstrated promising results in fire retardancy [6, 318]. The 3D graphene frameworks effectively increase the limiting oxygen index, representing the oxygen concentration required to sustain material combustion, by impeding the oxygen and heat transfer [319], thereby enhancing the flame-retardant properties. The stability of the strong *sp*^*2*^ carbon bonds and the capacity to confine nonflammable carbon dioxide gas within the pores imparts flame resistance to cellular graphene aerogels in air up to 1500 °C, surpassing that of graphene layers on substrates (~ 550 °C) [320]. Aligned composite aerogels, even with a low GO content of 10 wt%, exhibited notable fire retardancy, suppressing self-propagation of the flame [315]. While aligned graphene aerogels show promise in fire retardancy due to their low thermal conductivity perpendicular to the alignment direction and the significant barrier effect of the aligned walls to oxygen permeation, their full potential remains largely unexplored.

When coupled with PCMs, the aligned graphene aerogels can be applied for thermal adjustment purposes, effectively storing thermal energy at elevated temperatures and releasing it as the ambient temperature drops. These graphene aerogel/PCM composites exhibit the capacities for “energy regulation” and “energy inverse compensation,” leveraging the PCM's temperature regulation capacity [254, 321]. In an example inspired by spider webs, a graphene aerogel/PCM composite demonstrated effective thermal adjustment and management in battery systems, as illustrated in Fig. 25n, o [102]. The heat generated during battery operation was effectively transported to and stored within this phase-changing composite, thus preventing the accumulation of heat within the battery. Temperature raising in batteries during the charging and discharging process was impeded by this composite, thereby significantly decreasing potential safety risks and concurrently extending the operational lifespan of the batteries. A gradient GO aerogel/PCM composite structure with a moderate thermal conductivity of ~ 1 W m^−1^ K^−1^ was proved to be effective in thermal management in different environmental temperatures [252]. This composite exhibited the ability to maintain a higher skin temperature in cold environments and a lower localized surface temperature in hot environments.

### Organic Absorption

Organic absorption holds paramount importance in environmental remediation, aiding in the elimination of organic pollutants from water to safeguard ecosystems and human well-being. Over the past decade, graphene aerogels have emerged as potent organic-absorbing materials for environmental restoration and water treatment due to their exceptional porosity, structural stability, chemical resilience, and absorption capabilities [122, 282]. Taking inspiration from plant stems, materials featuring aligned pores provide efficient pathways for organic solvent and oil absorption, offering a solution for effective organic uptake. Specifically, graphene and graphene-based composite aerogels designed with aligned structures are hydrophobic and organophilic (repelling water and absorbing organic liquids), thereby facilitating the separation of oil and organic solvents from water [51, 71].

**Fig. 26:**
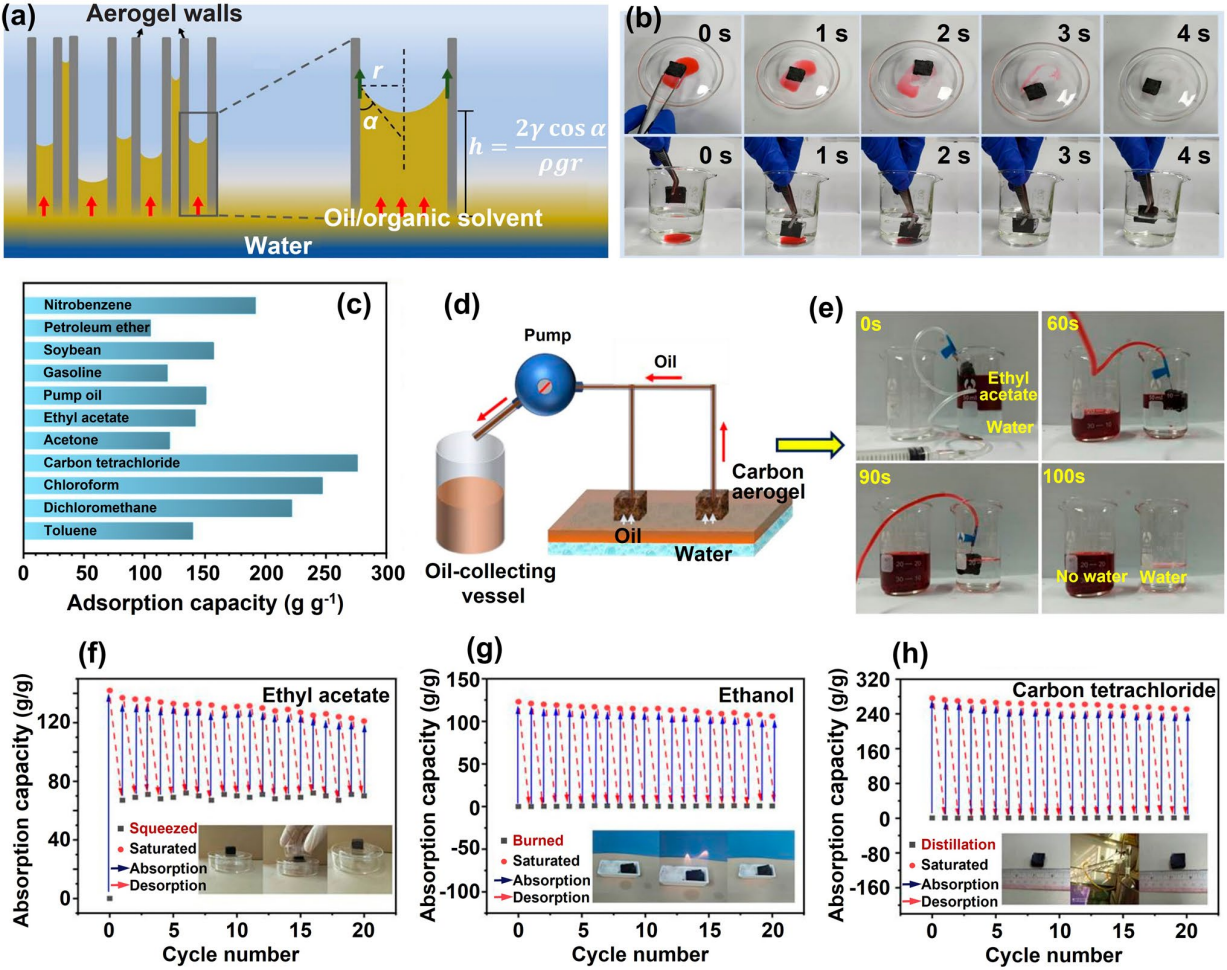
Absorption of oil or organic solvent of aligned graphene aerogel-based materials. **a** Capillary absorption mechanism, **b** absorption process, **c** absorption capacity, **d, e** oil water separation by continuous pumping, and recyclability via **f** squeezing, **g** burning, and **h** distilling. Reproduced with permission [36]. Copyright 2021, American Chemical Society

Organic solvent and oil absorption hinges on capillary forces, denoted as *F*, created when hydrophobic capillary-like porous structures of aligned aerogels come into contact with organic liquids, as shown in Fig. 26a [36]. As per Laplace's Law, the capillary force of the liquid in tubular structure is determined by Eq. (14):

14 $$F=\frac{2\gamma {\text{cos}}\alpha }{r}$$

where *γ* represents the surface tension of the liquid–air interface, *α* signifies the static contact angle, and *r* stands for the radius of the aligned channels [36]. According to this equation, as the channel radius diminishes, the capillary force increases, suggesting that micropores and mesopores contribute to a greater driving force for organic liquid absorption while macropores serve as storage spaces [322]. This capillary effect in aligned graphene and graphene-based composite aerogels equips them with a self-driven liquid absorption capability. The self-moved height, *h*, to which absorbable organic liquids rise is calculated using Eq. (15) [323]:

15 $$h=\frac{2\gamma {\text{cos}}\alpha }{\rho gr}$$

where *ρ* denotes the liquid density and *g* represents the local gravitational acceleration. Therefore, narrower capillary leads to higher automatic absorbing height. Thus, narrower capillaries lead to greater automatic absorbing heights.

The capillarity within aligned graphene aerogels can significantly accelerate absorption rates and achieve rapid absorption within the first 3–5 s and equilibrium within 10 s [277]. In Fig. 26b, a typical absorption process involving vertically aligned graphene-based aerogels is depicted, demonstrating the absorption of ethyl acetate and carbon tetrachloride stained with Sudan red [36]. Despite their varying densities in comparison to water, both organic solvents are efficiently absorbed upon contact with the aligned aerogel within a mere 4 s.

The absorption capacity, which is the weight ratio of material before and after organic absorption, is influenced by the density and viscosity of organic liquids. A hybrid aerogel composed of directionally freeze-cast and dried graphene/cellulose nanocrystals has demonstrated remarkable and liquid-density-dependent absorption capabilities, ranging from 105 to 276 g g^−1^, as shown in Fig. 26c [36]. A typical aligned graphene-based aerogel, structured with “lamellar layers and interconnected bridges,” showcases an absorption capacity of approximately 150 g g^−1^ for oil/organic solvents with relatively low densities, around 210–250 g g^−1^ for higher-density organic liquids and even up to 310 g g^−1^ for the densest oils [277]. While aligned micro-channels among lamellae facilitate swift absorption, the bridges connecting these lamellae enhance organic liquid storage and absorption capacity [276]. The absorption rate for viscous oils increases with temperature due to reduced oil viscosity and enhanced oil movement into the aerogel channels [276].

Potential applications of aligned graphene aerogels in oil/organic absorption were explored, focusing on the continuous separation of water and organic liquids through a systematic pumping system. As shown in Fig. 26d, e, a vertically aligned graphene-based aerogel was positioned on the liquid surface, and a needle was inserted into the aerogel structure. Upon opening of the pump, the resultant pressure differential actively facilitated the segregation of oil or organic solvents from the aqueous phase, which demonstrated the feasibility of employing aligned graphene aerogels for achieving efficient and continuous separation of immiscible liquid phases [277]. Considering the advantages of aligned graphene-based aerogels in effective solar-thermal energy transfer (transferring the solar energy into thermal energy, i.e. increased temperature of aerogels), the graphene aerogel and their composites can be designed with integrated solar-thermal convention to facilitate fast oil absorption [35].

Excellent compressibility and mechanical stability of aligned graphene-based aerogels play crucial roles in their efficient oil recovery and separation, as shown in Fig. 26f–h [36]. Squeezing is a straightforward and effective technique for oil/organic solvent recovery post-absorption, involving separation followed by mechanical compression [324]. Remarkable reusability, with less than a 3% reduction in absorption capacity after 10 cycles of absorption and squeezing, was achieved with bidirectionally freeze-dried, highly compressible graphene composite aerogels [33]. Besides squeezing, burning, and distilling of the saturated graphene aerogel were also demonstrated to be suitable for the reuse of the absorbing aerogels.

Besides the separation of the incompatible system of water and oil/organic solvents, aligned graphene-based aerogels are also effective in removal of soluble organics (such as dyes) from water. The absorption of soluble organics onto graphene-based aerogels can occur via chemical absorption following the pseudo-second-order kinetic equation [94], or physical absorption following the pseudo-first-order kinetic model [71]. The elimination of dyes is primarily driven by electrostatic interactions, wherein negatively charged oxygen-containing groups in partially reduced graphene sheets engage with positively charged dye molecules. Additionally, π-π conjugation occurs between the graphite regions within graphene sheets and the benzene rings present in the dye molecules.

### EMI Shielding

Electromagnetic pollutions generated by electronics are detrimental to human health and normal operation of electronic devices, making EMI shielding materials essential to manage and mitigate the adverse effects of electromagnetic waves. Highly porous graphene aerogels and their composites hold promises in EMI shielding, thanks to their decent electrical conductivities, low mass densities and tunable microstructures [34, 325]. Regulating the phase structure of framework walls and adjusting microstructures of aerogels are two effective methodologies to enhance EMI shielding capabilities of graphene aerogel [326].

The aligned graphene building blocks in anisotropic graphene aerogels and composites contribute to enhanced EMI shielding properties by synergistic effects of several aspects [29]. (i) The anisotropic microstructure of oriented graphene aerogels and composites increases electrical conductivities along the aligning direction, favoring electrical-conductivity-dependent interaction between incident radiations and conductive aerogel walls and thus increasing reflection- and adsorption-induced EMI SEs [171, 172]. (ii) Interacting with the larger effective surface area of cell walls in transversely aligned graphene aerogels undergo multiple wave reflections, enhancing EMI shielding [327, 328]. (iii) The interaction between graphene aerogels (or their composites) and incident electromagnetic radiations introduces dynamic polarization/relaxation in highly directional structures, which causes collective macroscopic arrangement of dipoles and further improves the attenuation of waves [82, 326, 329].

Based on these mechanisms, Zhao et al. developed MXene/graphene/epoxy composites by infiltrating epoxy into the aligned MXene/graphene aerogel, showing a total EMI SE of 56.4 dB for composites with a filler content of 0.74 vol% and a 2 mm thickness [330]. Similarly, a graphene/MXene/Ni composite exhibited substantial wave absorption of 99.999996%, corresponding to a reflection loss of − 72.5 dB at a 2.15 mm thickness [331]. An EMI shielding SE of 112.2 dB, an implication of ~ 99.9999999994% blocked incident radiation, and a specific SE divided by the thickness of 173,243 dB cm^2^ g^−1^ were achieved by graphene aerogels prepared via chemical and thermal expansion of GO films [275]. These advancements all demonstrate the potential aligned graphene-based aerogels for EMI shielding applications. the anisotropic EMI shielding capabilities of highly aligned graphene aerogels enable the transition between EMI shielding (perpendicular to the alignment) and transmission (along the alignment), expanding their potential applications in various industries, such as wireless communication devices and aerospace.

## Conclusions and Perspectives

In conclusion, this review highlights the recent advances in highly aligned graphene aerogels and their composites, emphasizing their unique structural characteristics, characterizations, properties, and potential applications. With the assistance of directional freeze casting technique, the self-assembly of GO LC precursors and the mechanical shearing, graphene aerogels with highly oriented walls and porous channels are fabricated. The alignment of graphene building blocks can be characterized both qualitatively and quantitatively using SEM observation, orientation distribution analysis of SEM images, Polarized Raman spectroscopy and X-ray scattering. Aligned graphene aerogels take full advantages of the remarkable in-plane mechanical, electrical, and thermal properties of the 2D graphene sheets, showing anisotropic characteristics in mechanical, electrical, thermal transport and EMI shielding properties (better performances along one particular direction at the sacrifice of transverse directions). Super-elasticity, super-compressibility and fatigue resistance are endowed by the aligned scaffolds, making aligned graphene aerogels ideal for piezoelectric sensing of pressure and compressive strain. The anisotropic thermal conductivities of graphene aerogels and their composites make them possible in various thermal-related applications, such as solar thermal energy transfer/storage, solar steam, thermal conductors, and thermal insulators. The capillary effect of oriented porous channels in highly aligned graphene-based aerogels makes them promising in organic solvent/oil absorption. Besides, they also exhibit advantages in applications like electrochemical devices and EMI shielding. The main challenges and possible solutions are proposed as follows:

(i) One of the primary challenges in the field of highly aligned graphene aerogels is the development of scalable, controllable, and cost-effective fabrication techniques that can reliably achieve and maintain the desired level of alignment. While there have been significant advancements in the fabrication of aligned structures, many existing techniques are limited in terms of scalability and cost-efficiency, hindering their practical implementation on a larger scale. Continuous research efforts focusing on the optimization of fabrication process and the exploration of innovation approaches such as multi-step processes or hybrid techniques that combine different alignment methods are contributive. Implementing robotic systems and automated quality control for precise handling, alignment, and assembly processes can also enhance efficiency, reduce labor costs, and improve reproducibility.
(ii) GO suspensions are currently used as precursors for the fabrication of graphene aerogels, but reduction process that often involves harsh chemical treatments or high-temperature annealing is necessary for partial restoration of the exceptional graphene properties. This limits the overall quality and large-scale fabrication of graphene aerogel-based products. Developing high-quality, large-size graphene as building blocks and effective functionalization techniques to ensure good dispersity and compatibility hold significant promise for advancing the aligned graphene aerogels. Advancements in refining exfoliation parameters to minimize defects, maximize lateral sizes and better control over the number of layers may obtain graphene sheets with desired properties. Selectively functionalizing specific regions of graphene sheets with chemical groups or functional moieties (for example, functionalized graphene edges and well-crystalline planes) can improve dispersibility, stability, and compatibility of graphene with various matrices while maintaining the excellent intrinsic properties of graphene at a highest level, benefiting to further enhancement of aligned graphene aerogel-based materials.
(iii) Integrating multifunctionalities of highly aligned graphene aerogel-based materials by integrating multifunctionalities to produce all-in-one devices is one of the effective ways to promote their practical applications. By combining multiple functionalities within a single material or device, a wide range of applications can be addressed more efficiently and effectively. The unique properties of highly aligned graphene aerogels, such as their high surface area, excellent mechanical strength, anisotropic thermal conductivity, and electrical properties, make them ideal candidates for multifunctional integration. For example, integration of sensing and actuation functionalities within the aligned graphene aerogel-based materials. By incorporating sensing elements into the aerogel structure, it can be used for strain or pressure sensing applications. Furthermore, the excellent mechanical properties of aligned graphene aerogels enable them to function as actuator materials in response to external stimuli, such as temperature or humidity changes. These multifunctional materials can find applications in smart structures, robotics, and wearable devices.

## Abbreviations

3D: Three-dimensional
CNT: Carbon nanotube
CVD: Chemical vapor deposition
EMI: Electromagnetic interference
FWHM: Full width at half maximum
GF: Graphene foam
GNP: Graphene nanoplatelet
GO: Graphene oxide
GRGC: Gradient reduced graphene oxide aerogel-based bilayer phase change material composite
GWF: Graphene woven fabric
HOPG: Highly-ordered pyrolytic graphite
KOH: Potassium hydroxide
LC: Liquid crystal
LGO: Large graphene oxide
MGW: Multilayer graphene web
MXene: Transition metal carbides and nitrides
PANI: Polyaniline
PCM: Phase change material
PDMS: Polydimethylsiloxane
PEI: Poly(ethylenimine)
PI: Polyimide
PS: Polystyrene
rGO: Reduced graphene oxide
rGOF-DS: Reduced graphene oxide aerogels with a certain disordered structure
SAXS: Small-angle X-ray scattering
SE: Shielding effectiveness
SEI: Solid-electrolyte interphase
SEM: Scanning electron microscopic
SGO: Small graphene oxide
TIM: Thermal interface material
UV: Ultraviolet
VA-GSM: Vertically aligned reduced graphene oxide aerogels
WAXS: Wide-angle X-ray scattering
